# Novel long non-coding RNAs of relevance for ulcerative colitis pathogenesis

**DOI:** 10.1016/j.ncrna.2022.02.001

**Published:** 2022-02-06

**Authors:** Mithlesh Kumar Ray, Christopher G. Fenton, Ruth H. Paulssen

**Affiliations:** a Clinical Bioinformatics Research Group, Department of Clinical Medicine, UiT-The Arctic University of Norway, Tromsø, Norway; b Genomic Support Centre Tromsø (GSCT), Department of Clinical Medicine, UiT-The Arctic University of Norway, Tromsø, Norway

**Keywords:** Ulcerative colitis, lncRNAs

## Abstract

**Background and aims:**

The study aimed to identify yet unknown and uncharacterized long non-coding RNAs (lncRNAs) in treatment-naïve ulcerative colitis (UC), and to define their possible roles in UC pathogenesis. For that purpose, accurate quantification methods for lncRNA transcript detection, multiple and “stringent” strategies were applied. New insights in the regulation of functional genes and pathways of relevance for UC through expression of lncRNAs are expected.

**Methods:**

The study was based on sequencing data derived from a data set consisting of treatment-naïve UC patients (n = 14) and control subjects (n = 16). Two complementary aligners were used to identify lncRNAs. Several different steps were used to validate differential expression including plotting the reads over the annotation for manual inspection. To help determine potential lncRNA involvement in biological processes, KEGG pathway enrichment was done on protein-coding genes which co-expressed with the lncRNAs.

**Results:**

A total of 99 lncRNAs were identified in UC. The lncRNAs which were not previously characterized (n = 15) in UC or other autoimmune diseases were selected for down-stream analysis. In total, 602 protein-coding genes correlated with the uncharacterized lncRNAs. KEGG pathway enrichment analysis revealed involvement of lncRNAs in two significantly enriched pathways, lipid and atherosclerosis, and T-cell receptor signaling.

**Conclusion:**

This study identified a set of 15 yet uncharacterized lncRNAs which may be of importance for UC pathogenesis. These lncRNAs may serve as potential diagnostic biomarkers and might be of use for the development of UC treatment strategies in the future.

## 1 Background

Ulcerative colitis (UC) is a chronic inflamed condition of the colon and rectum and one of the major phenotypes of inflammatory bowel disease (IBD) [1]. Despite the prevalence of UC, the etiology of UC is poorly understood. The UC pathogenesis is complex and an interplay between environmental factors, intestinal microbiome, nutrition and genetic factors [1]. Although heritability plays a potential role, only a small fraction (7.5–22%) of UC risk can be explained by genetic factors alone [2,3]. Genome-wide association studies (GWAS) found several IBD risk loci on the non-coding region of the genome [4]. LncRNAs have not been thoroughly explored in IBD [5] nor has their contribution to the progression of the disease.

LncRNAs play an important role in tumor development and carcinogenesis and have been suggested to be biomarkers for diagnosis and prognosis [[6], [7], [8]]. A growing body of evidence implies a role for lncRNAs in UC [[9], [10], [11]]. The expression of lncRNAs in UC has previously been reported [9,10,12,13]. They are involved in the modulation of the intestinal barrier function [13,14], regulating expression of inflammatory cytokines [15], and polarization of macrophages [16].

LncRNAs, which are RNAs with a length greater than 200 nucleotides, are poorly conserved [17]. Their roles in gene expression regulation are still not well understood [18]. They may or may not be polyadenylated, and 98% are spliced. At least two different alternatives spliced isoforms have been observed in about 25% of all known lncRNAs [19]. LncRNAs share common features as they are expressed at lower levels, are tissue-specific, and have exonic regions with low levels of interspecies sequence conservation [20]. Weak expression makes accurate quantification of lncRNA transcripts particularly challenging. According to ENCODE's own evaluation, less than 1000 lncRNAs are present at greater than one copy per cell in the typical human tissue culture cell lines [21]. In addition, many lncRNA exons overlap protein-coding exons on the same strand making it difficult to determine the origin of the transcript counts. To ensure the veracity of differentially expressed lncRNAs, several complementary methods need to be employed. Determining lncRNA function is difficult, but protein-coding transcripts that co-express with lncRNA transcripts may offer some insight into lncRNA function. Likewise, pathway enrichment of co-expressed protein-coding genes may offer insight into relevant biological pathways involved in UC pathogenesis.

## 2 Materials and methods

### 2.1 Patient data

Gene expression data of mucosal gene expression were obtained from the Gene expression Omnibus (GEO) GSE128682 and represent sequencing data obtained from mucosal biopsies of treatment-naïve UC patients (n = 14) and normal control subjects (n = 16) [22].

### 2.2 Data analysis

**Fig. 1:**
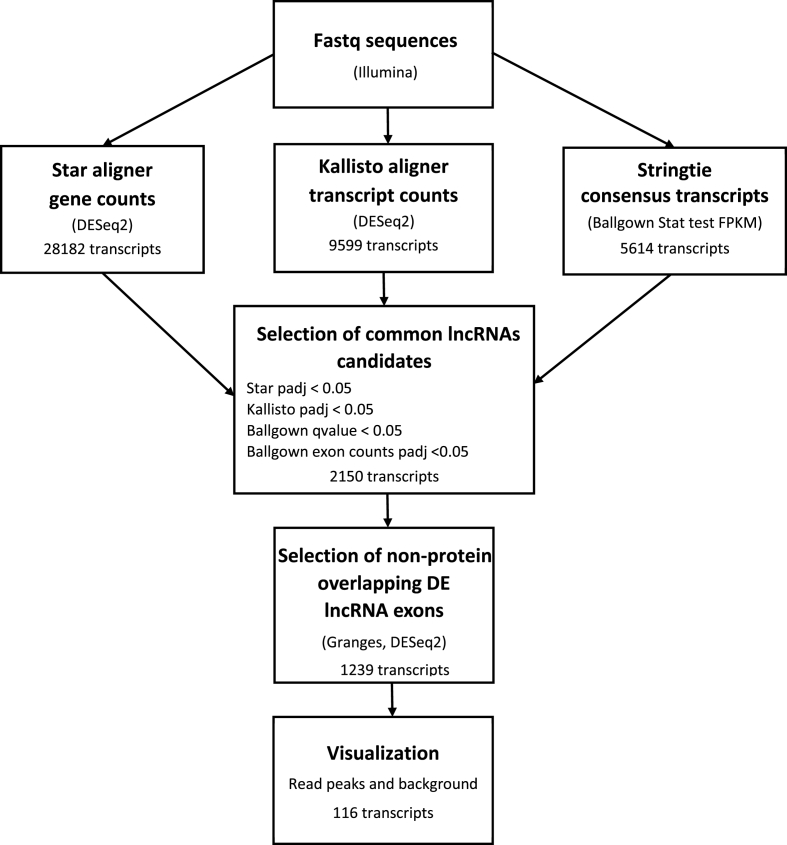
Flow diagram representing the outline of experimental steps. Fastq data was aligned using several methods Star, Kallisto, and Ballgown. Differential expression was estimated by DESeq2 for Star (gene counts) and Kallisto (transcript counts), stattest for Ballgown (FPKM). LncRNA candidates were significantly differentially expressed in all three tests. GRanges was used to find non protein overlapping lncRNA exons. Ballgown unique exon counts and DESeq2 were used to ensure that candidates had at least one differentially expressed non protein overlapping exon. Bam read counts were then plotted over genome annotation to ensure exon read count alignment to annotation and comparison to background noise.

A schematic overview of the data analysis methodological approach is shown in Fig. 1. The Gencode v36 (GRCh38.p13) reference genome (https://www.gencodegenes.org/) [23] was used for all alignments, annotations and visualization methods. All tests for differential expression were between UC and normal samples. Both Star aligner and Kallisto were used to align the Illumina generated fastq sequences. Star was used to generate a gene count matrix. Kallisto was used to create a transcript count matrix. DESeq2 was used to find DE genes and DE transcripts with an adjusted p value less than 0.05. LncRNAs were defined as those with transcript type or gene type equals lncRNA in the annotation gtf file. Stringtie v2.0.3 (https://github.com/gpertea/stringtie/releases) [24] was used to create a consensus set of transcripts from the Star aligned bam files. The Ballgown (https://www.bioconductor.org/packages/release/bioc/html/ballgown.html) [25] stattest using transcript FPKM as a metric was used to generate transcript q values from the set of Stringtie consensus transcripts. Granges is a software package that can identify genomic overlaps (https://bioconductor.org/packages/release/bioc/html/GenomicRanges.html) [26]. Granges was used to isolate lncRNA exons that did not overlap with known protein coding exons on the same strand. A matrix of Ballgown unique exon counts was created from the non-overlapping lncRNA exons. DESeq2 was used to identify differentially expressed exons from the exon matrix, adjusted p value < 0.05. Differentially expressed lncRNA met the following conditions: Star(gene) padj <0.05, Kallisto(transcript) padj <0.05, Ballgown (FPKM) qvalue <0.05, and at least one non protein overlapping lncRNA exon with padj <0.05. Only lncRNA transcripts with an average read count greater than 16 were considered.

**Fig. 2:**
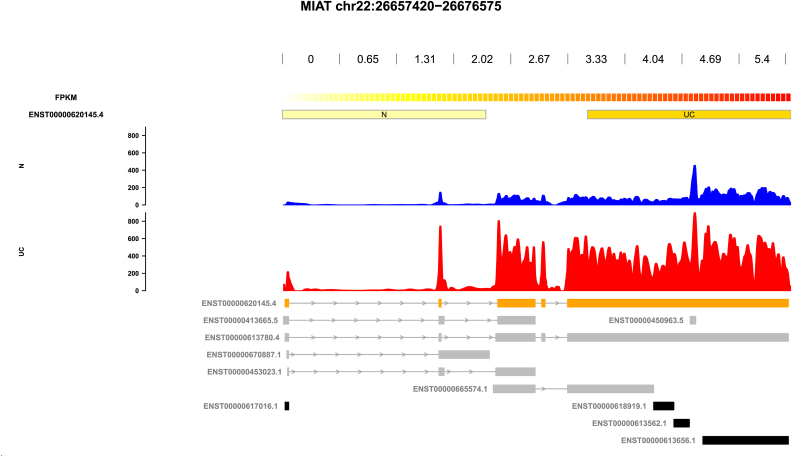
Visualization of lncRNA candidates. LncRNA gene symbol and location are indicated in figure title. The upper part of the figure shows the Ballgown FPKM values for each lncRNA transcript labeled on the left. Low FPKM values are white/light yellow, higher FPKM values are darker orange/red. The lower part of the figure shows the average read counts for UC and normal controls over the genomic annotation. Read counts are shown on the lower panel y axis. Normal controls read counts are indicated in blue (n = 16) and UC read counts are indicated in red (n = 14). The genomic annotation used to align is shown under the read counts. Transcripts are labeled on the left. LncRNA transcripts in orange are considered valid candidates. Transcripts in grey are lncRNA transcripts that were not considered candidates. Transcripts in black are not annotated as lncRNA.

LncRNA annotation is constantly updated, therefore Biomart was used to check that the remaining differentially expressed lncRNA transcript type was currently annotated as lncRNA (https://bioconductor.org/packages/release/bioc/html/biomaRt.html). in latest Ensembl annotation. Finally, each significantly lncRNA was inspected visually (Fig. 2). By using Samtools [27] the read coverage for each candidate lncRNA region was extracted directly from the STAR aligned Bam files. LncRNA transcript read coverage was plotted over the genome reference exon structure using the Gviz [28] package. LncRNAs whose read coverage peaks aligned with reference exons, was greater than the local background, and did not completely overlap non lncRNA reference exons (Fig. 2) were considered as candidates.

Co-expression analysis was used to select potential protein coding target transcripts for the fifteen uncharacterized lncRNAs depicted in Table 1. Transcripts with an absolute Pearson correlation greater than 0.85 were selected. If total lncRNA transcript targets were less than thirty, the top thirty most co-related transcripts were taken into consideration. The R ReactomePA package (https://bioconductor.org/packages/release/bioc/html/ReactomePA.html) [29] was used to find significantly enriched KEGG [30] using gene names of the co-expressed uncharacterized lncRNA transcripts.

**Table 1:** List of uncharacterized lncRNAs in ulcerative colitis (UC).

Transcript ID	Gene_Name	Ballgown (qvalue)	Kallisto (FC)	Kallisto (padj)	Star (baseMean)	Star (padj)	Exon (padj)
ENST00000669835.1	AC110611.2	2.92E-06	1.51	2.38E-15	22.1	1.28E-14	1.33E-24
ENST00000669140.1	AL354743.2	0.002	1.03	3.56E-05	33.01	0	1.12E-09
ENST00000424989.1	LINC01137	0.004	0.56	0.009	86.22	0.004	1.16E-05
ENST00000606723.2	U91328.1	6.39E-05	−0.79	1.21E-06	107.54	5.27E-17	0
ENST00000553330.1	LINC02313	0.04	−0.84	0	20.52	7.32E-07	0.02
ENST00000447171.2	AC007255.1	0.002	−0.93	3.66E-06	155.91	7.06E-10	8.46E-05
ENST00000661542.1	AL353572.4	2.94E-05	−1.11	7.55E-13	35.5	5.29E-18	6.16E-07
ENST00000658026.1	LINC02405	0.018	−1.12	0	82.57	1.76E-08	0.001
ENST00000451240.1	AC005550.2	0.007	−1.26	1.10E-05	170.78	1.18E-08	0
ENST00000432368.2	THRB-AS1	5.95E-05	−1.42	2.73E-11	10.52	1.06E-10	1.19E-08
ENST00000656535.1	AC007114.1	0.033	−1.5	5.19E-05	41.7	0	0
ENST00000416416.1	GORAB-AS1	0.017	−1.53	5.89E-05	45.44	2.71E-09	0
ENST00000553425.5	AL121790.2	0.001	−1.66	1.90E-07	58.31	2.26E-07	2.60E-06
ENST00000512915.5	AC098487.1	0.036	−1.98	5.37E-05	21.08	5.78E-14	1.10E-07
ENST00000664281.1	AC116345.4	0	−2.13	1.37E-09	81.42	2.34E-13	4.18E-09

## 3 Results

### 3.1 Differentially expressed lncRNAs in treatment-naïve ulcerative colitis

DESeq2 on the STAR generated gene count matrix gave a total of 8615 differentially expressed (DE) lncRNA genes (padj <0.05) coding for a total of 28182 lncRNA transcripts (Fig. 1). Ballgown stattest (q value < 0.05) using FPKM values from Stringtie consensus sequences gave a total of 5614 DE lncRNA transcripts belonging to 4254 lncRNA genes. DESeq2 was used to perform differential expression analysis on transcripts obtained from the Kallisto aligner, which gave 9599 DE lncRNAs transcripts (padj <0.05) belonging to 6720 lncRNA genes. DESeq2 on the non-protein overlapping exon unique counts matrix resulted in 4073 lncRNA transcripts with at least one DE non-overlapping exon (padj <0.05). Combining the lncRNA results Ballgown (FPKM) qvalue <0.05, Star (gene) padj <0.05, Kallisto (transcript) padj <0.05, and non-protein overlapping exons (Ballgown unique exon count) padj <0.05 resulted in 2150 lncRNA candidates. Of the 2150 candidates, 1239 candidates were verified as biotype lncRNA in the latest ensemble annotation by a BioMart query (Supplementary Table 1). The entire analysis flowchart is shown in Fig. 1.

**Fig. 3:**
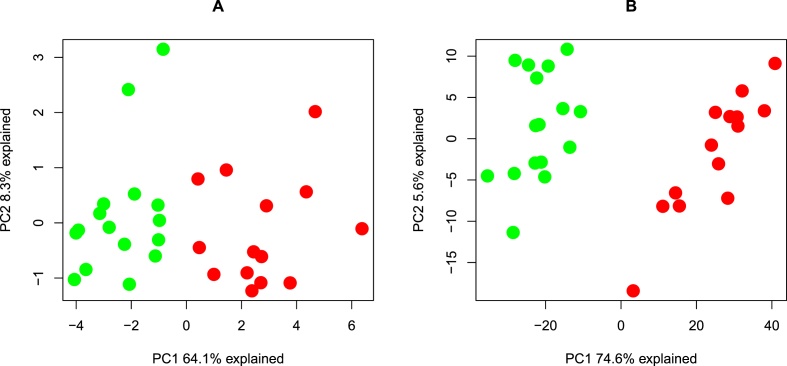
Principal component analysis (PCA). (A) PCA depicting 15 uncharacterized lncRNA transcripts presenting the difference between UC (n = 14; red)) and normal controls (n = 16; green). The first two components explain 64.1% and 8.3% of the variability in the lncRNA expression data. (B) PCA of differentially expressed coding transcripts (n = 686) which correlate to the uncharacterized lncRNAs (n = 15) presenting the difference between UC (n = 14; red) and normal controls (n = 16; green). The first two components explain 74,6% and 5.6% of the variability in the expression data.

Visual inspection of lncRNAs candidates was done by plotting read coverage of lncRNAs over the exon structure defined in the genome reference annotation (Fig. 2). A total of 116 lncRNAs transcripts representing 99 lncRNA candidate genes were selected (Supplementary Fig. 1). Seven of the candidate lncRNAs were previously found to be dysregulated in IBD, fourteen have been observed in colorectal cancer, and six were related to inflammation and infection (Supplementary Table 2). All these 99 significantly differentially expressed lncRNAs are depicted in a heat map (Supplementary Fig. 2). Among these 99, fifteen lncRNAs have not been previously described and characterized in UC (Table 1). Principal component analysis (PCA) using the uncharacterized lncRNAs showed a clear separation between UC samples and normal samples. Principal component 1 (PC1) explained 64.1% of the total variance (Fig. 3A).

### 3.2 Co-expression of lncRNAs with protein-coding genes

The 15 uncharacterized lncRNAs were then subjected to correlation analysis, which resulted in a total of 602 co-expressed protein-coding genes in correlation analysis (coefficient absolute 0.85 with correlation p-value < 0.05) (Supplementary Table 3). In addition, a PCA was performed on the differentially expressed protein-coding transcripts (n = 686) which correlated with the expression of the uncharacterized lncRNAs (Fig. 3B). Here, principal component (PC1) explained 74.6% of the total variance and a clear separation of UC and normal samples was seen. LncRNAs AC110611.2, GOARB-AS1, AC005550.2, and AC116345.4 were co-expressed with 190, 170,112, and 65 protein-coding transcripts, respectively. Correlation analysis showed that multiple protein-coding transcripts can co-express with a single lncRNA transcript and *vice vers*a (Supplemental Table 3). Among the co-expressed transcripts were several protein-coding genes which related to inflammation and UC progression like interleukin 1B (IL-1B) [31], metalloproteinase 3 (MMP3), metalloproteinase 9 (MMP9) [32], and Vav guanine nucleotide exchange factor 3 (VAV3) [33]. Several pro-inflammatory cytokines such as IL-33, TNFSF10 and IL21R co-expressed with AC110611.2.

### 3.3 Pathway enrichment analysis

**Fig. 4:**
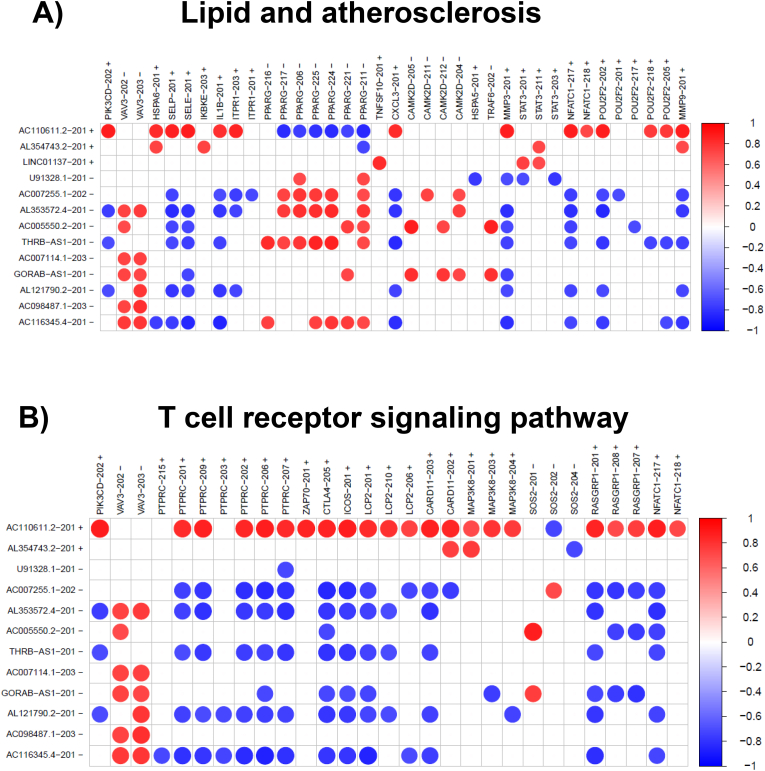
Co-expression plot of KEGG enriched pathways between lncRNA transcripts and their correlated protein coding transcripts. Co-expression plots of lipid and atherosclerosis pathway (A) and T cell receptor signaling (B) are indicated. LncRNA transcripts are listed on the x-axis, correlated protein coding transcripts on the y-axis. Transcript names are followed by a ‘+’ (UC expression greater than N expression) or a ‘-’ (N expression less than UC expression). Red dots indicate lncRNA transcript and protein coding transcript expression are positively correlated. Blue dots indicate where the lncRNA transcript and protein coding transcript expression are negatively correlated. Only correlations with an absolute value greater than 0.85 are shown.

Genes corresponding to the correlated protein-coding transcripts were used for KEGG pathway enrichment. Two significantly enriched pathways with padj and qvalue <0.05 could be identified, the T cell receptor pathway and the lipid and atherosclerosis pathway. Seventeen and twelve genes, which co-expressed with the uncharacterized lncRNAs were found to be enriched in both pathways. Among them VAV3, lymphocyte cytosolic protein 2 (LCP2), and inducible T cell co-stimulator (ICOS), both of which play a role in vascular endothelial cell integrity [34], NK-cell mediated recognition of missing-self targets [35], and effective T-helper-cell responses [36]. To illustrate the correlations an example of a co-expression is shown in Fig. 4.

## 4 Discussion

In this study, differentially expressed lncRNAs in treatment-naïve UC were explored by applying accurate quantification methods for lncRNA transcript detection. This study provides new knowledge of 15 previously uncharacterized lncRNAs which may be involvedin the regulation of the lipid and atherosclerosis and T cell receptor signaling pathways.

Accurate quantification of lncRNA transcripts is challenging. Therefore, several complementary methods along with visual inspection were applied to generate a set of lncRNAs that distinguish between UC samples and controls (Fig. 1). The majority of lncRNA transcripts are expressed at a significantly lower level than protein coding transcripts, making lncRNA transcription levels difficult to distinguish from the background noise [37]. Recent RNAseq studies have shown differences in intra-exonal coverage, which could have aroused from naturally occurring splice variants sharing part of an exon or could have been due to technical errors in library construction or sequencing [38]. In addition, some lncRNA's exons overlap with other non-lncRNA exons, making it difficult to determine the origin of read counts [39]. Lower counts and overlaps present challenges for lncRNA quantification. Therefore, only lncRNAs containing at least one differentially expressed lncRNA exon that did not overlap a protein coding exon were considered for this study. An example is given in Fig. 2, showing the lncRNA myocardial infarction associated transcript (MIAT). The MIAT read counts map well to the MIAT lncRNA exon annotation and aligns to a greater extent than protein-coding exons. This suggests that the majority of read counts come from MIAT exons and not any protein coding exon overlaps. The MIAT read counts in Fig. 2 are greater than the local background. Plotting the read counts over the annotation strengthened the ability to quantify lncRNA accurately.

Initial PCAs of the uncharacterized lncRNAs (Fig. 3A) and the corresponding correlated protein-coding transcripts (Fig. 3B) revealed a clear separation of UC samples from normal samples in both cases. This indicates that the chosen sample size is satisfactory to make assumptions on the significance of the results. KEGG enrichment analysis of uncharacterized lnc transcripts correlating with protein coding genes revealed in only two significantly enriched pathways, the lipid and atherosclerosis pathway, and the T cell receptor signaling pathway. The correlation plots for the two pathways are depicted in Fig. 4.

In the lipid and atherosclerosis pathway, lncRNA AC001611.2 expression correlated positively with four genes (MMP3, MMP9, IL-1 and CXCL3) whereas six other lncRNAs correlated negatively with the same genes (Fig. 4A). Perhaps these lncRNAs are involved in the modulation of inflammatory cytokines production, and immune cells migration during UC by regulating the expression of matrix metallopeptidases. A connection between impaired intestinal integrity, cytokine production, and monocytes migration has been reported to be associated with atherosclerosis [[40], [41], [42]]. A relationship between UC and atherosclerosis has been implicated [[43], [44], [45], [46], [47]]. The reported higher risk of cardiovascular events in UC patients may be pertinent in inflammation-mediated atherosclerosis [[48], [49], [50]] as inflammation and atherosclerosis have been proposed to share similar pathogenesis [51]. Therefore, the identified and previously unknown lncRNAs might qualify for possible new prognostic factors for UC patients with atherosclerosis.

LncRNAs may also play a role in T cell apoptosis during UC. LncRNAs AL354743.2 and LINC0113 correlated positively with the STAT3 transcription factor which induces the transcription of BCL2 and BCL-XL in T cells. The expression of these anti-apoptotic genes can increase the resistance of pathogenic T cells of lamina propria to apoptosis, leading to prolonged inflammation [52].

The T cell receptor-signaling pathway was the second significantly enriched pathway. Several genes involved in this pathway such as PTPRC (CD45), NFATc1, and RASGRP1 were differentially expressed in UC (Fig. 4B). The expression of PTPRC (CD45), a known IBD susceptibility gene, correlated positively with lncRNA AC110611.2 and correlated negatively with six lncRNAs depicted in Fig. 4B. Here, the lncRNAs might contribute to the activation of Cd4+ T cells which are key players in mediating the host protective and homeostatic responses to inflammation [53]. It is interesting to note that these lncRNAs might also play a role in the regulation of the expression of different patterns of alternatively spliced CD45 isoforms that have been shown to be associated with distinct functions [54]. T cell activation of cytokine production is also regulated by the expression of NFATc1 and RASGRP1 both of which correlated positively with lncRNA AC110611.2 and correlated negatively correlated with lncRNAs AC007255.1, AL353572.4, THRB-AS1, AL121790.2, and AC116345. Interestingly, RASGRP1 promotes inflammatory responses by enhancing the production of IL-6 by sponging with miRNA let-7a [55]. IL-6 has been shown to be positively associated with UC development and regulates intestinal barrier function via STAT3 [56].

The expression of lncRNA AC110611.2 correlated with numerous protein-coding transcripts (Supplementary Table 3). Apart from genes involved in the pathways discussed above (Fig. 4), several other genes co-expressed with AC110611.2 including many regulators of inflammatory immune responses such as ICOS, IL-21, Il-21R, and Sema7A [[57], [58], [59]].

Many of the up-regulated lncRNAs found by this methodological approach have been already identified and shown to be associated with IBD pathogenesis (Supplementary Table 2), such as small integral membrane protein 25 (SMIM25) [60], IFNG antisense RNA 1 (IFNG-AS1) [61], and DIO3 opposite strand RNA (DIO3OS) [62]. The observed downregulation of CDKN2B-AS1 is negatively correlated with inflammatory cytokines expression responsible for UC progression [12]. The upregulation of LINC01871 might indicate a dysregulation of T cell inflammatory responses in UC as has been reported for several other autoimmune diseases [63,64]. Overall, our study gives an insight into novel lncRNAs which potentially be involved in intestinal barrier function and immune cell development, activation, and migration. However, loss- and gain-of-function studies are required to verify the biological importance of expression of these lncRNA by *in vitro* and *in vivo* experiments. To what extent the uncharacterized lncRNAs contribute to the regulation of the T-cell receptor signaling pathway during UC progression has to be explored in more depth in the future.

## 5 Conclusion

This study revealed 15 lncRNAs, which have not been functionally annotated previously and which may be involved in the pathogenesis of UC. The applied methodological approaches together with a visual inspection of read counts over the annotation was key to identifying lncRNA's that were differentially regulated. The results may provide new potential diagnostic biomarkers and therapeutic targets for ulcerative colitis which may improve the understanding of the molecular pathogenesis of UC. However, if lncRNAs are going to be of use as future biomarkers for UC, more reliable approaches for lncRNAs profiling and reliable lncRNA quantification methods are required.

## Funding

Not applicable

## Ethical approval and consent to participate

Not applicable.

## Consent for publication

Not applicable.

## Availability of data

All data are within the manuscript and its Supporting Information files.

## CRediT authorship contribution statement

**Mithlesh Kumar Ray:** Formal analysis, Validation, writing, Software, reviewing the final draft. **Christopher G. Fenton:** Data curation, Methodology, Investigation, Visualization, Validation, Software, Writing – review & editing. **Ruth H. Paulssen:** Conceptualization, Investigation, Project administration, Resources, Methodology, Supervision, writing, Writing – original draft.

## Declaration of competing interest

The authors declare no conflict of interests regarding the publication of this manuscript.
