# Supplementary material for: Novel long non-coding RNAs of relevance for ulcerative colitis pathogenesis
Source: Noncoding RNA Res. 2022 Feb 6;7(1):40–7. doi: 10.1016/j.ncrna.2022.02.001 (PMC8844606; doi:10.1016/j.ncrna.2022.02.001)
Supplement: Multimedia component 1 [file mmc1.pdf]

**Supplementary figure 1**. All lncRNAs (99) visually selected by plotting over lncRNA reference annotations.

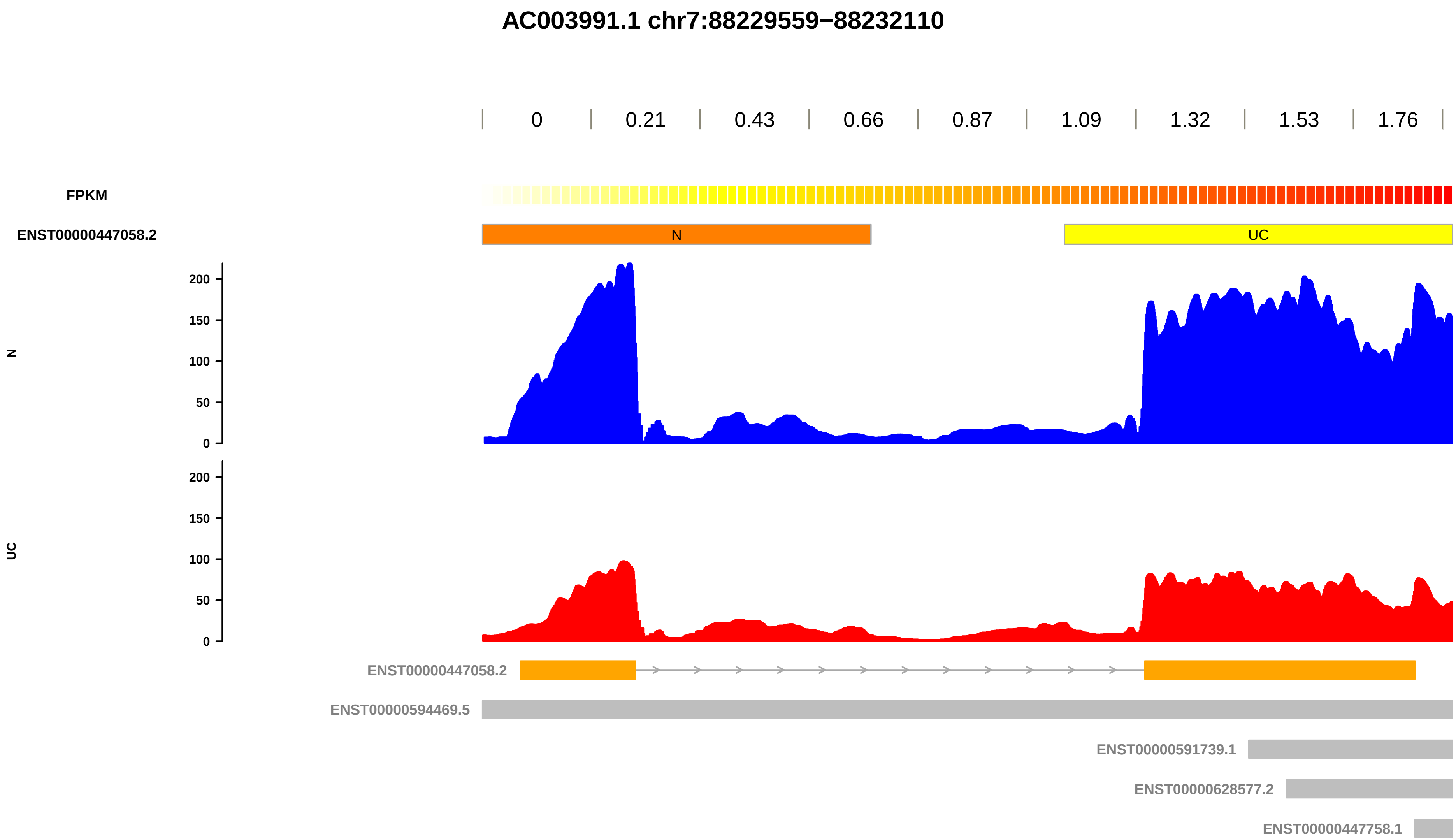

AC003991.1 chr7:88229559−88232110
0
0.21
0.43
0.66
0.87
1.09
1.32
1.53
1.76
FPKM
ENST00000447058.2
N
UC
N
UC
200
150
100
50
0
ENST00000447058.2
ENST00000594469.5
ENST00000591739.1
ENST00000628577.2
ENST00000447758.1

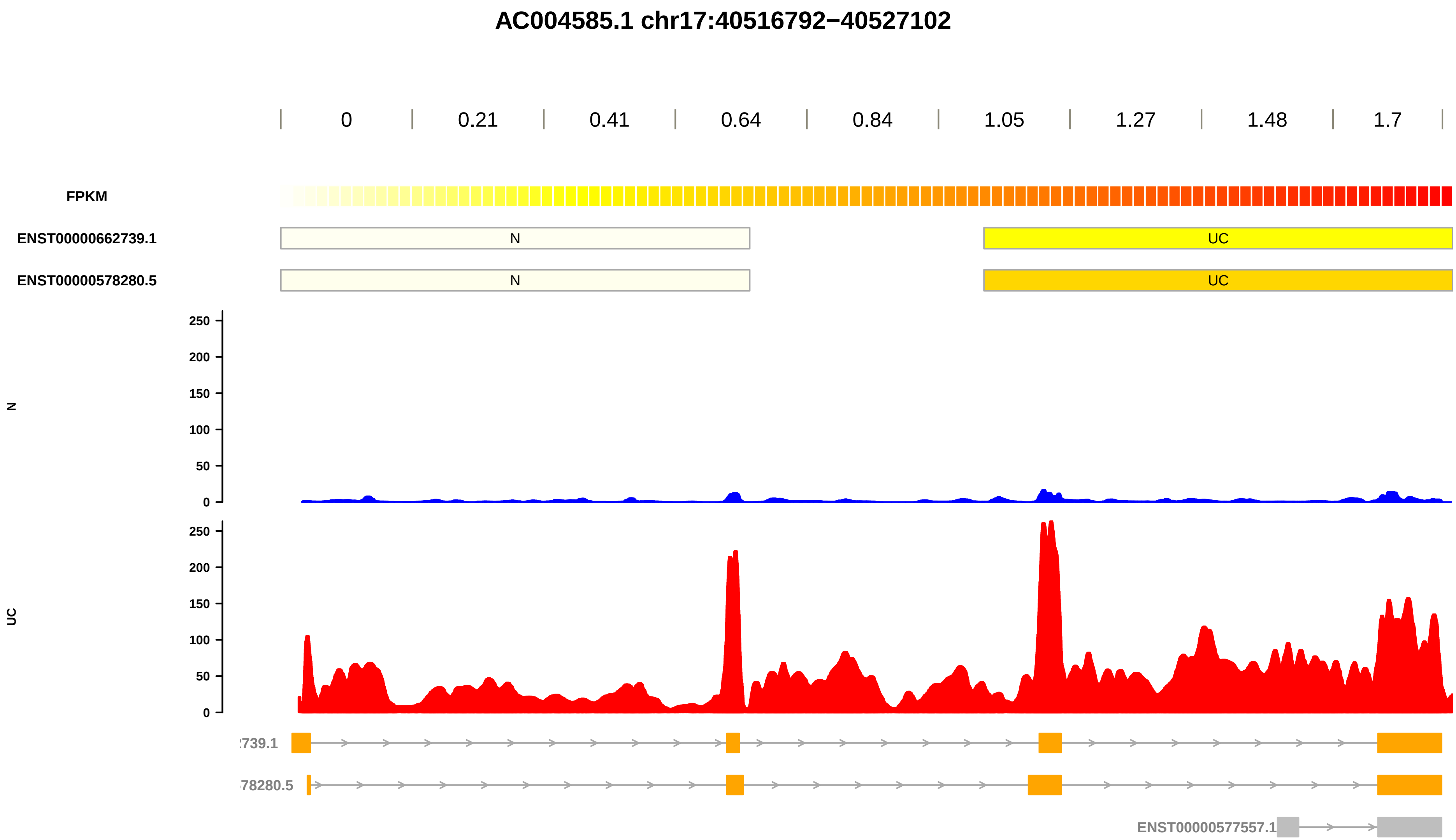

AC004585.1 chr17:40516792−40527102
0
0.21
0.41
0.64
0.84
1.05
1.27
1.48
1.7
FPKM
ENST00000662739.1
N
UC
ENST00000578280.5
N
UC
N
250
200
150
100
50
0
UC
250
200
150
100
50
0
739.1
78280.5
ENST00000577557.1

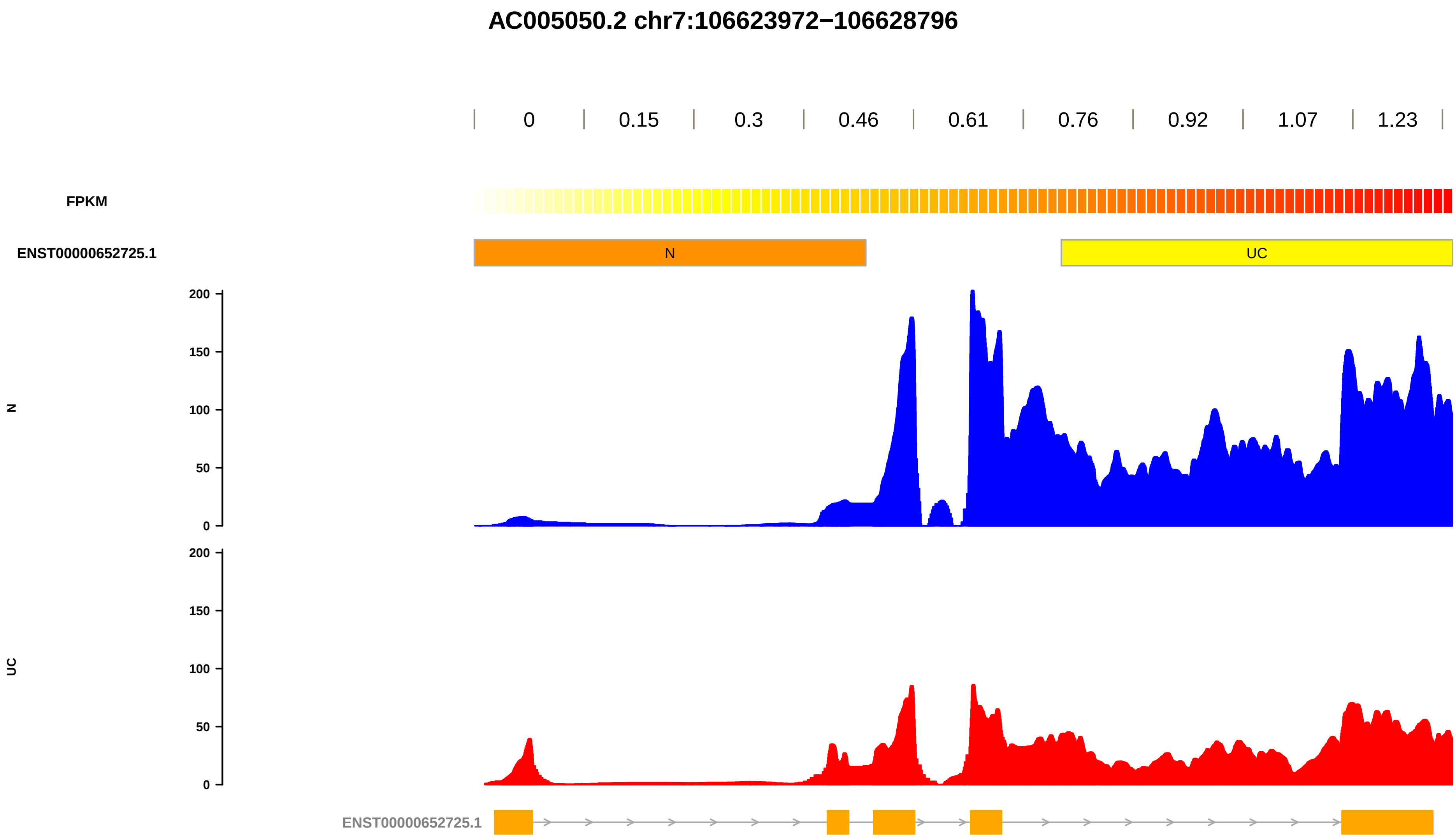

AC005050.2 chr7:106623972−106628796
0
0.15
0.3
0.46
0.61
0.76
0.92
1.07
1.23
FPKM
ENST00000652725.1
N
UC
200
150
100
50
0
N
200
150
100
50
0
UC
ENST00000652725.1

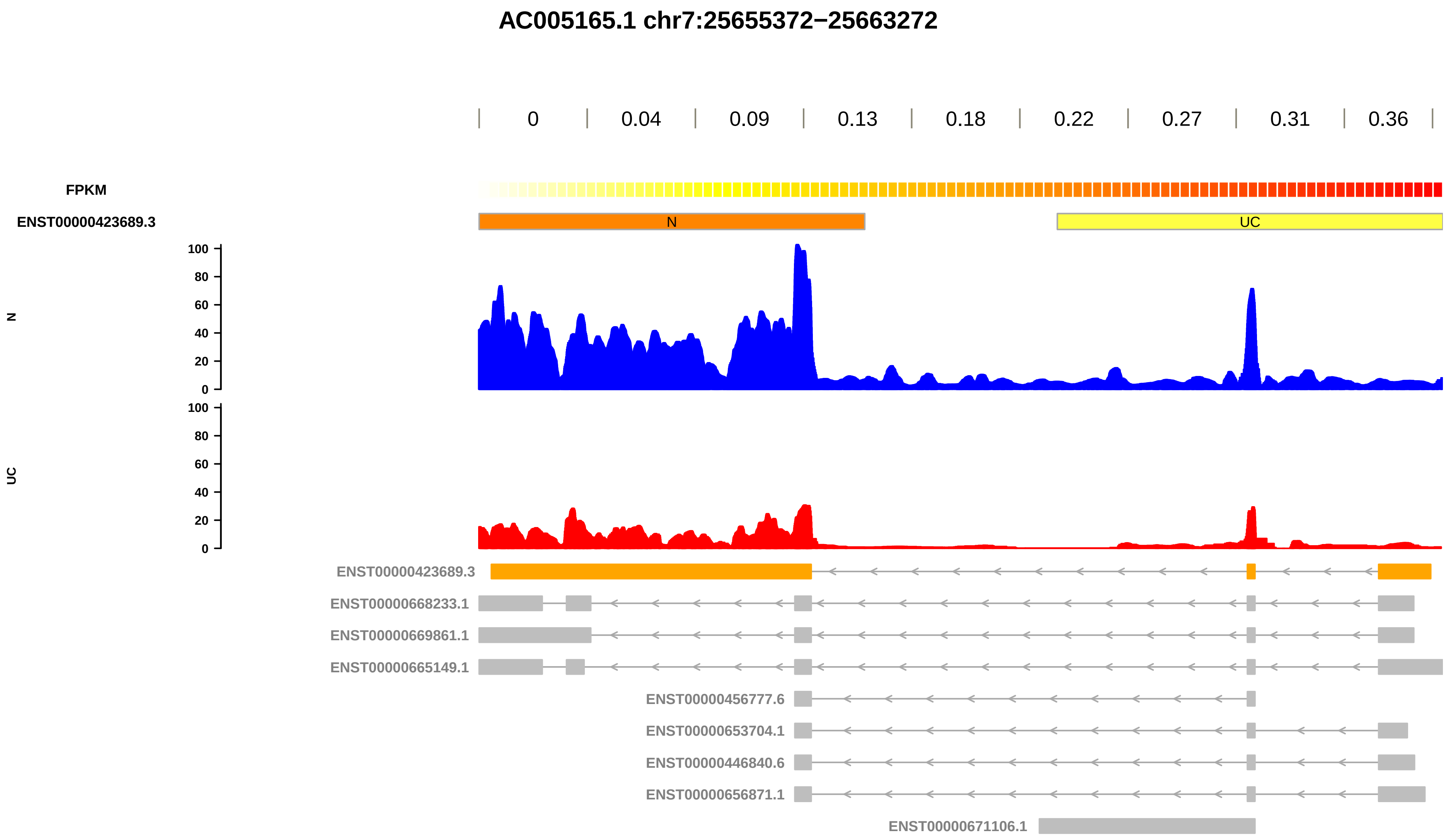
AC005165.1 chr7:25655372−25663272
0
0.04
0.09
0.13
0.18
0.22
0.27
0.31
0.36
FPKM
ENST00000423689.3
N
UC
100
80
60
40
20
0
ENST00000423689.3
ENST00000668233.1
ENST00000669861.1
ENST00000665149.1
ENST00000456777.6
ENST00000653704.1
ENST00000446840.6
ENST00000656871.1
ENST00000671106.1

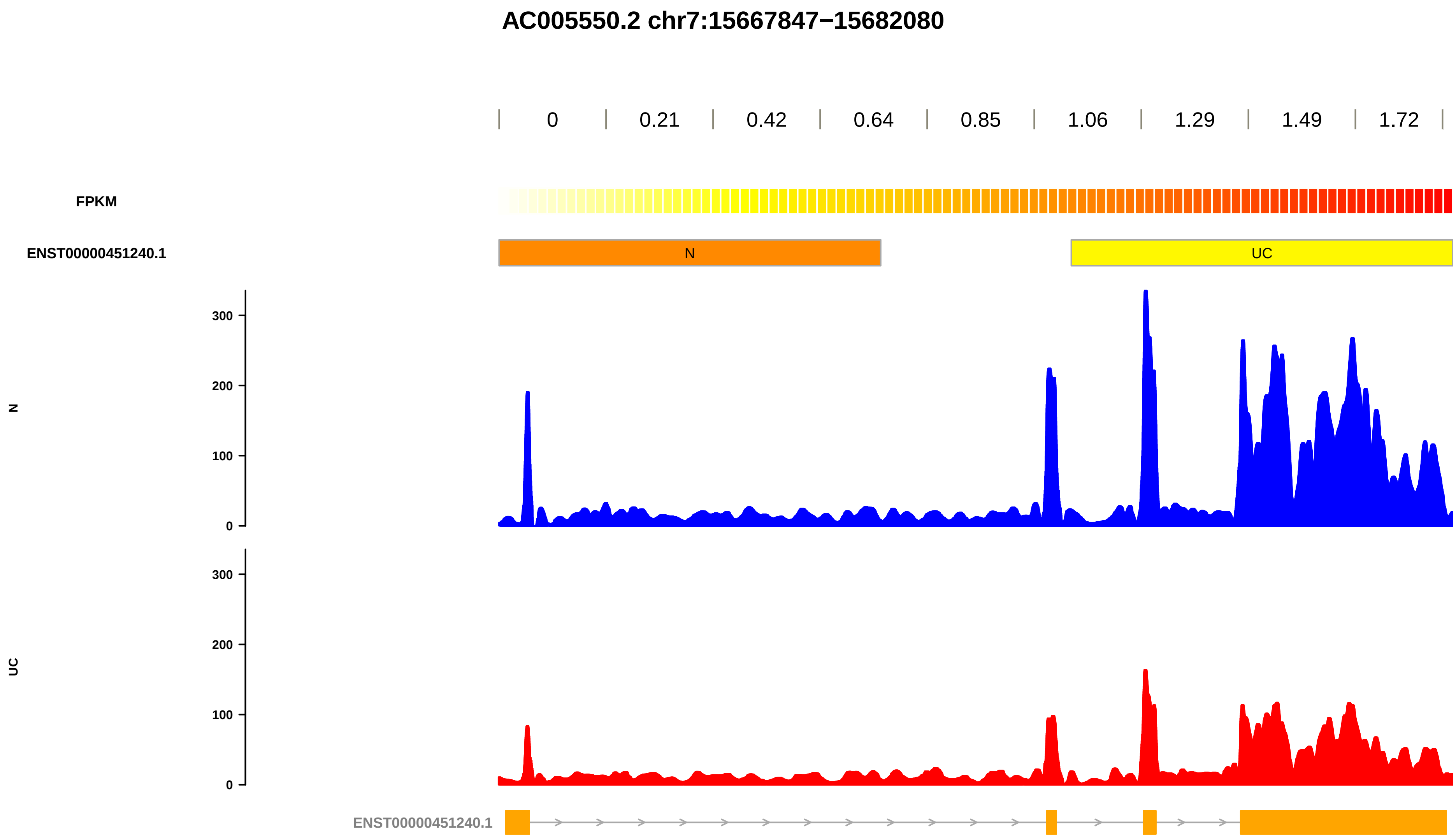
AC005550.2 chr7:15667847−15682080
0
0.21
0.42
0.64
0.85
1.06
1.29
1.49
1.72
FPKM
ENST00000451240.1
N
UC
300
200
100
0
N
300
200
100
0
UC
ENST00000451240.1

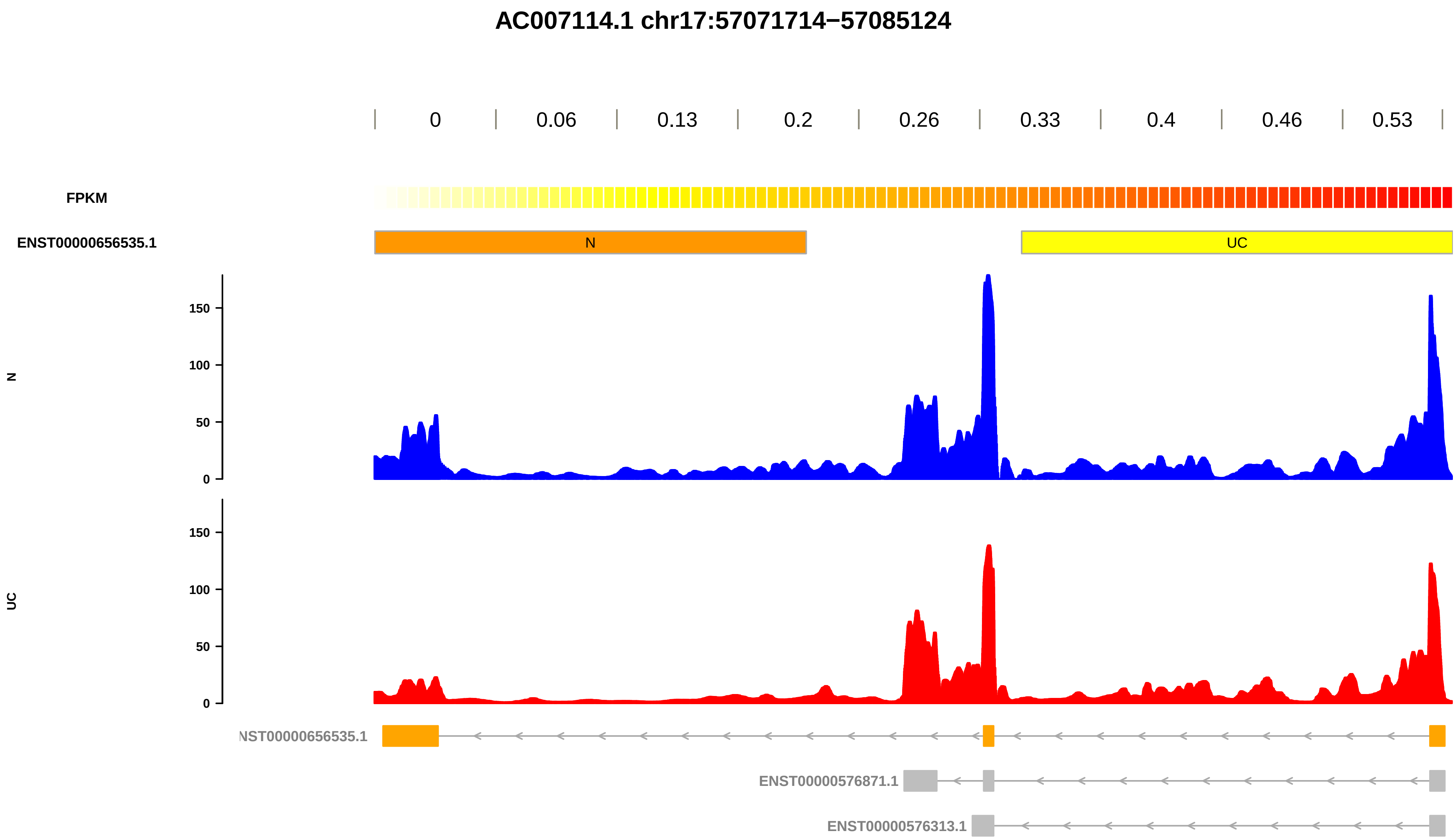

AC007114.1 chr17:57071714−57085124
0
0.06
0.13
0.2
0.26
0.33
0.4
0.46
0.53
FPKM
ENST00000656535.1
N
UC
N
150
100
50
0
UC
150
100
50
0
NST00000656535.1
ENST00000576871.1
ENST00000576313.1

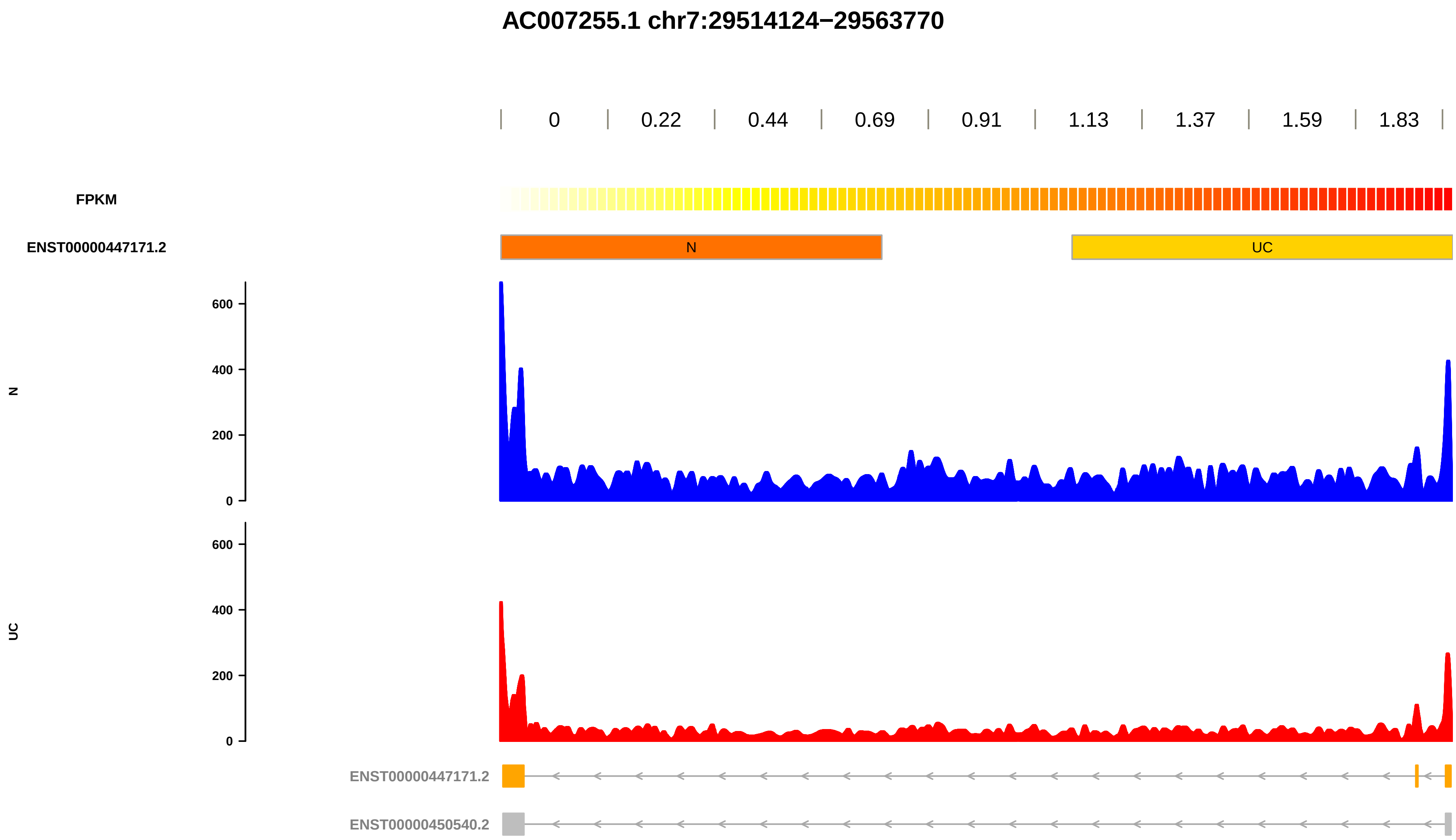
AC007255.1 chr7:29514124−29563770
0
0.22
0.44
0.69
0.91
1.13
1.37
1.59
1.83
FPKM
ENST00000447171.2
N
UC
600
400
200
0
N
600
400
200
0
UC
ENST00000447171.2
ENST00000450540.2

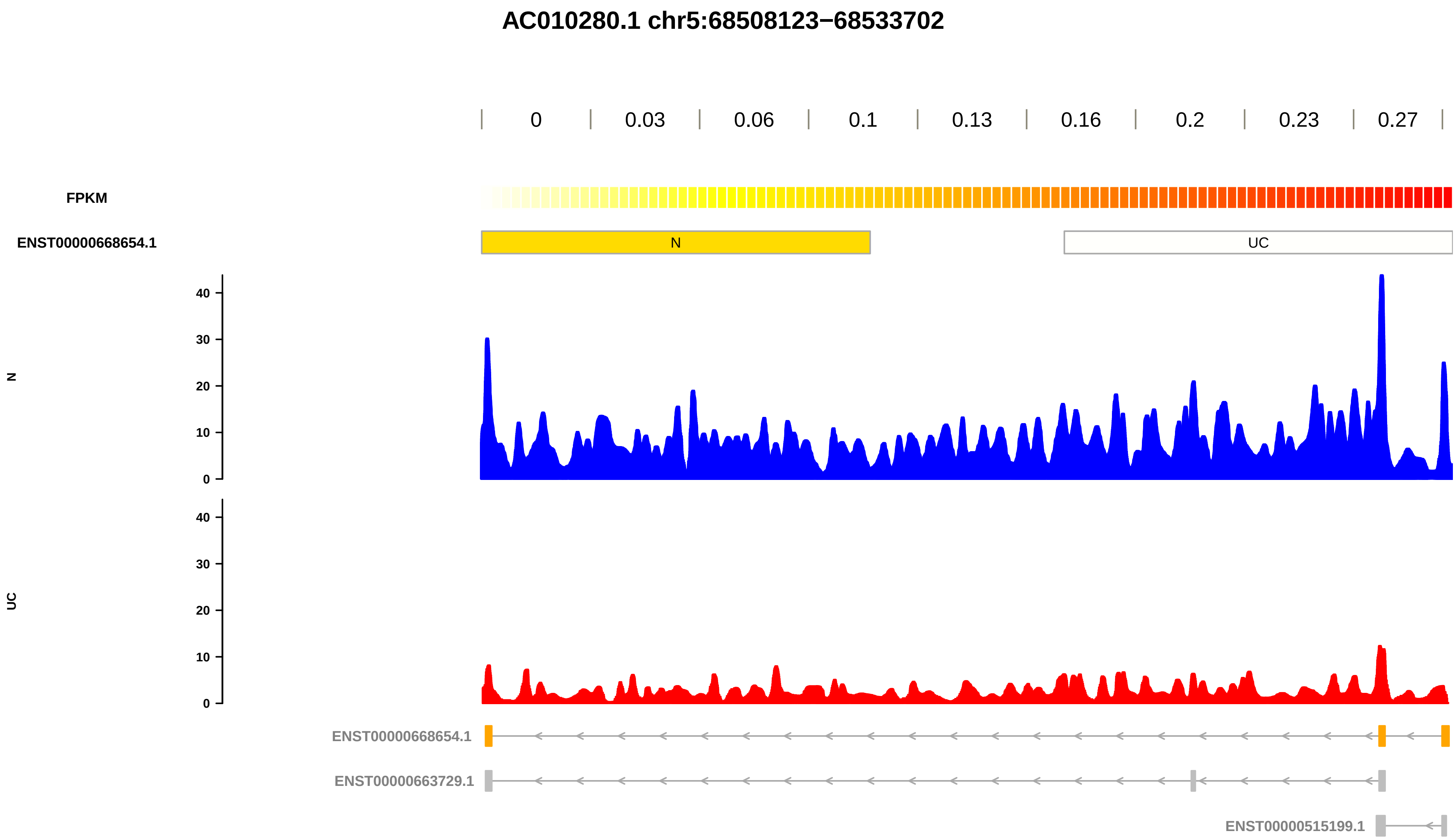
AC010280.1 chr5:68508123−68533702
0
0.03
0.06
0.1
0.13
0.16
0.2
0.23
0.27
FPKM
ENST00000668654.1
N
UC
N
UC
40
30
20
10
0
ENST00000668654.1
ENST00000663729.1
ENST00000515199.1

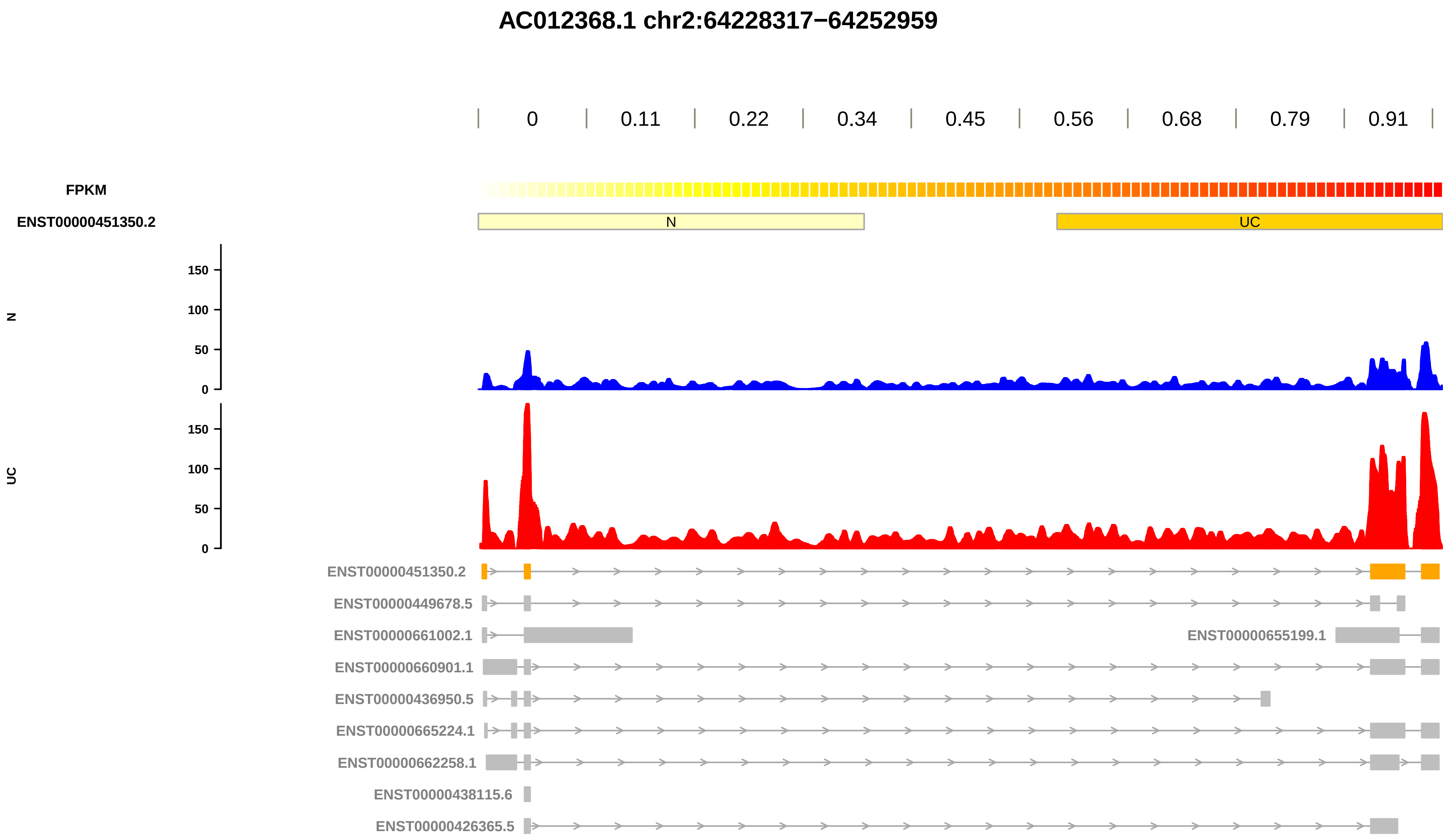

AC012368.1 chr2:64228317−64252959
0
0.11
0.22
0.34
0.45
0.56
0.68
0.79
0.91
FPKM
ENST00000451350.2
N
UC
150
100
50
0
ENST00000451350.2
ENST00000449678.5
ENST00000661002.1
ENST00000655199.1
ENST00000660901.1
ENST00000436950.5
ENST00000665224.1
ENST00000662258.1
ENST00000438115.6
ENST00000426365.5

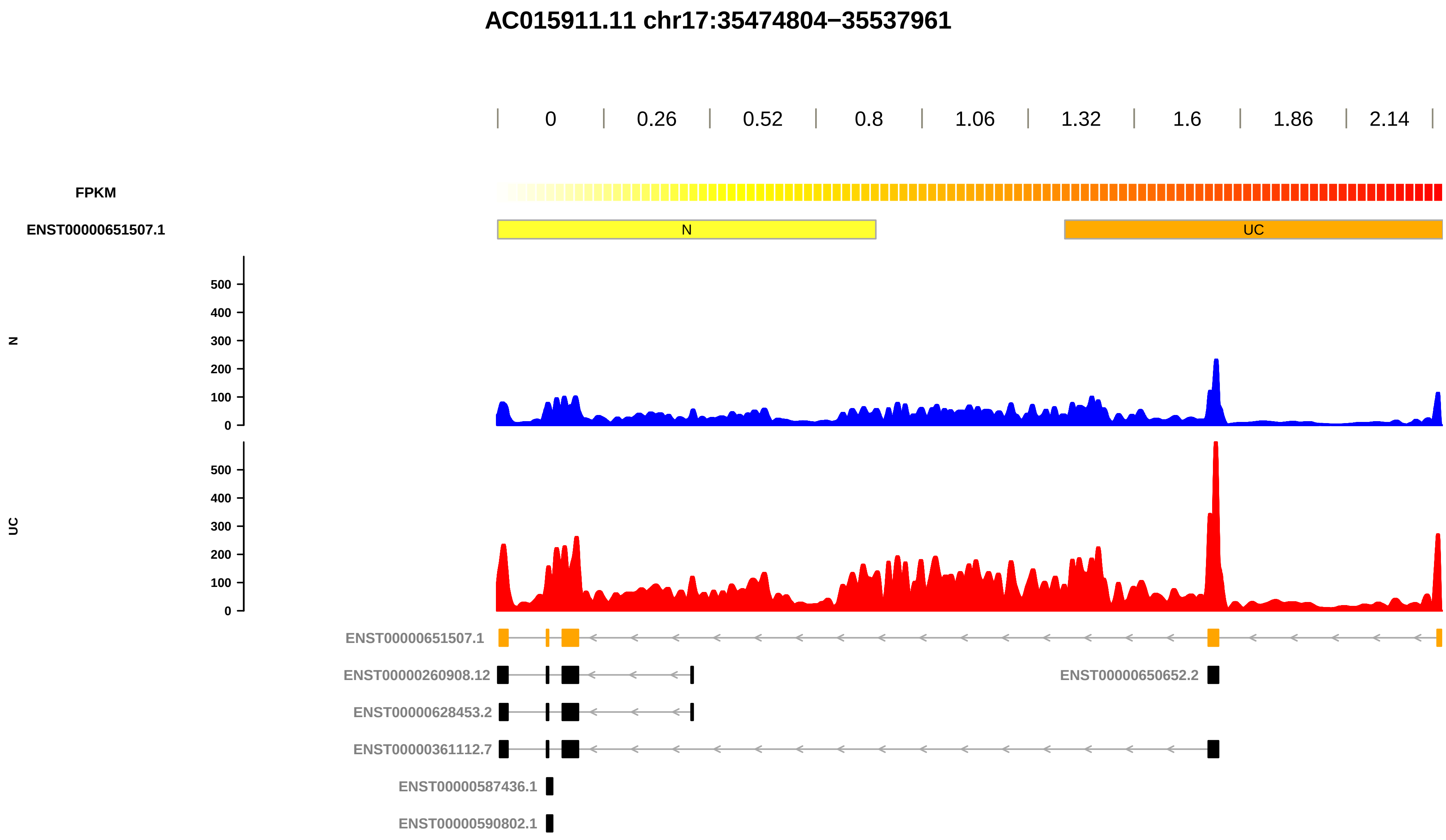
AC015911.11 chr17:35474804−35537961
0
0.26
0.52
0.8
1.06
1.32
1.6
1.86
2.14
FPKM
ENST00000651507.1
N
UC
500
400
300
200
100
0
ENST00000651507.1
ENST00000260908.12
ENST00000650652.2
ENST00000628453.2
ENST00000361112.7
ENST00000587436.1
ENST00000590802.1

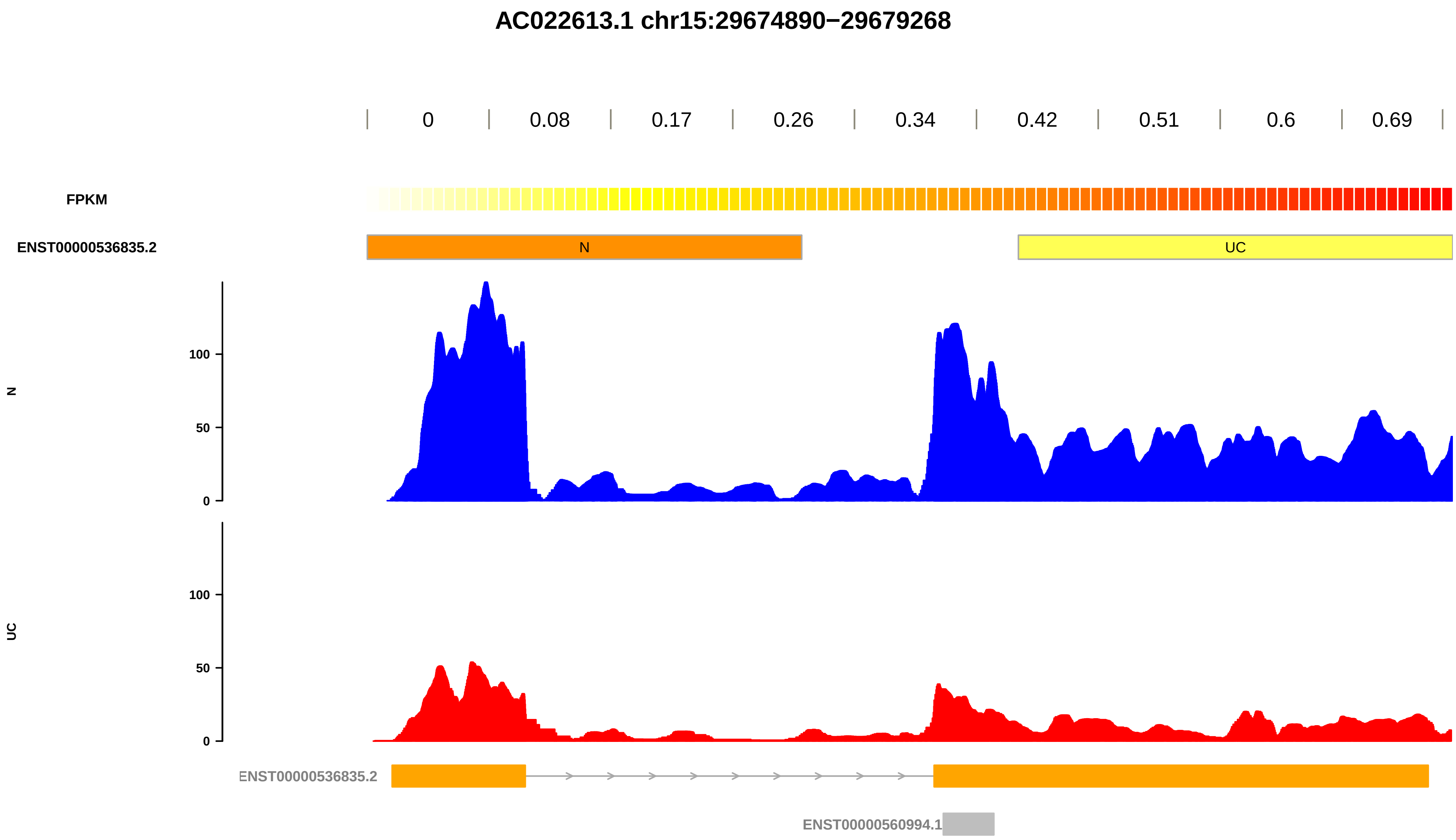
AC022613.1 chr15:29674890−29679268
0
0.08
0.17
0.26
0.34
0.42
0.51
0.6
0.69
FPKM
ENST00000536835.2
N
UC
100
50
0
N
100
50
0
UC
ENST00000536835.2
ENST00000560994.1

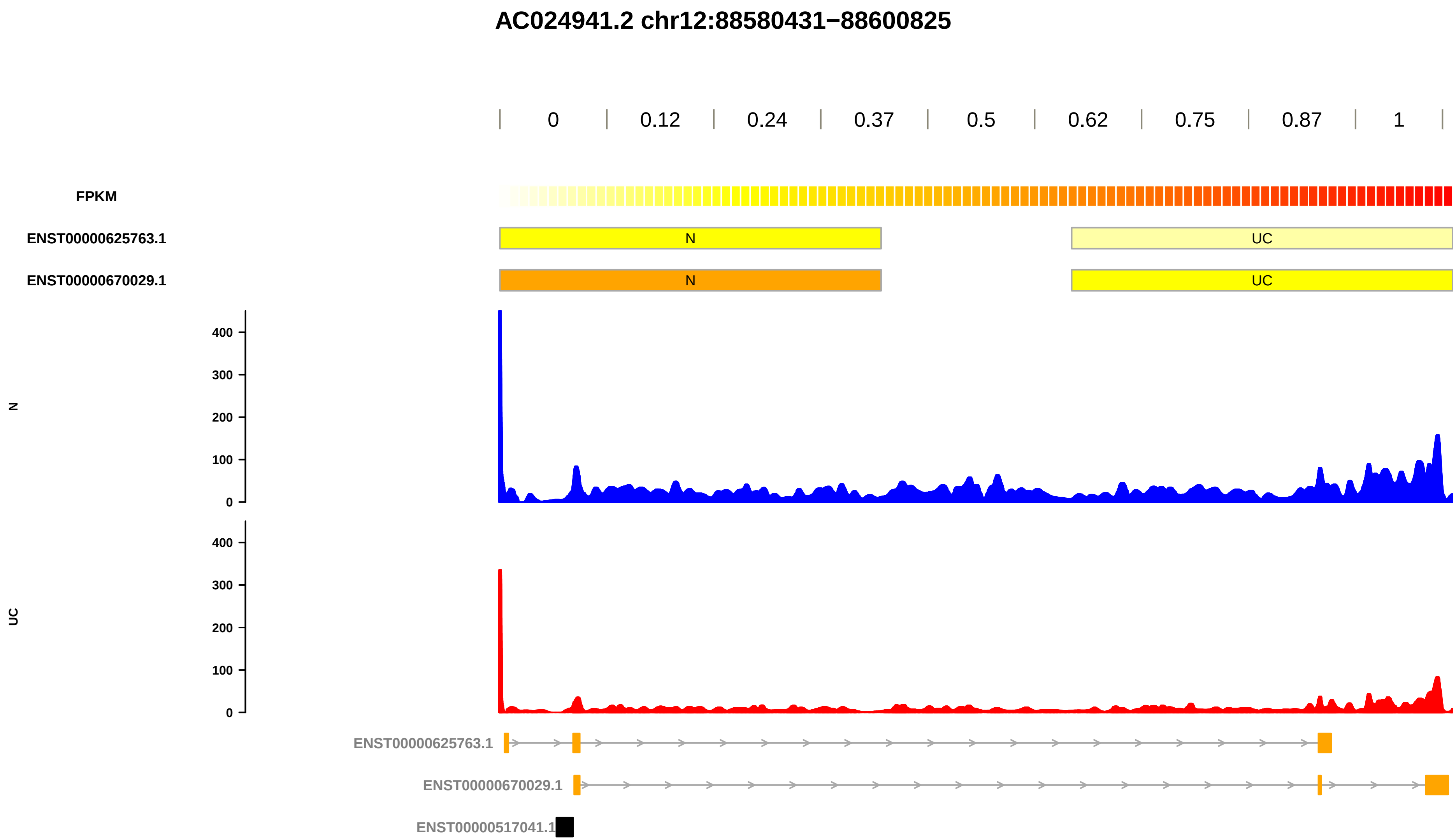
AC024941.2 chr12:88580431−88600825
0
0.12
0.24
0.37
0.5
0.62
0.75
0.87
1
FPKM
ENST00000625763.1
N
UC
ENST00000670029.1
N
UC
N
400
300
200
100
0
UC
400
300
200
100
0
ENST00000625763.1
ENST00000670029.1
ENST00000517041.1

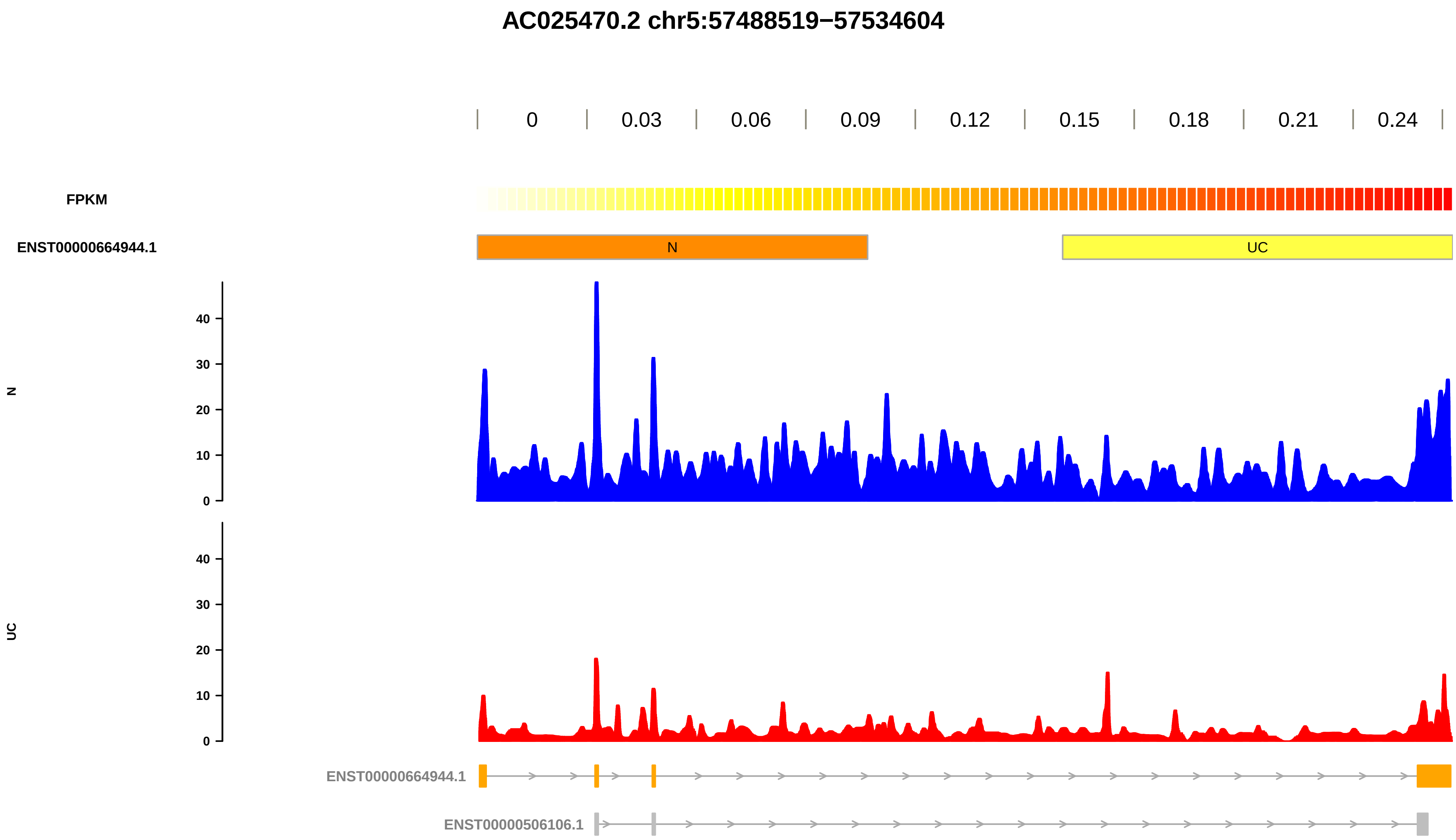

AC025470.2 chr5:57488519−57534604
0
0.03
0.06
0.09
0.12
0.15
0.18
0.21
0.24
FPKM
ENST00000664944.1
N
UC
N
UC
0
10
20
30
40
ENST00000664944.1
ENST00000506106.1

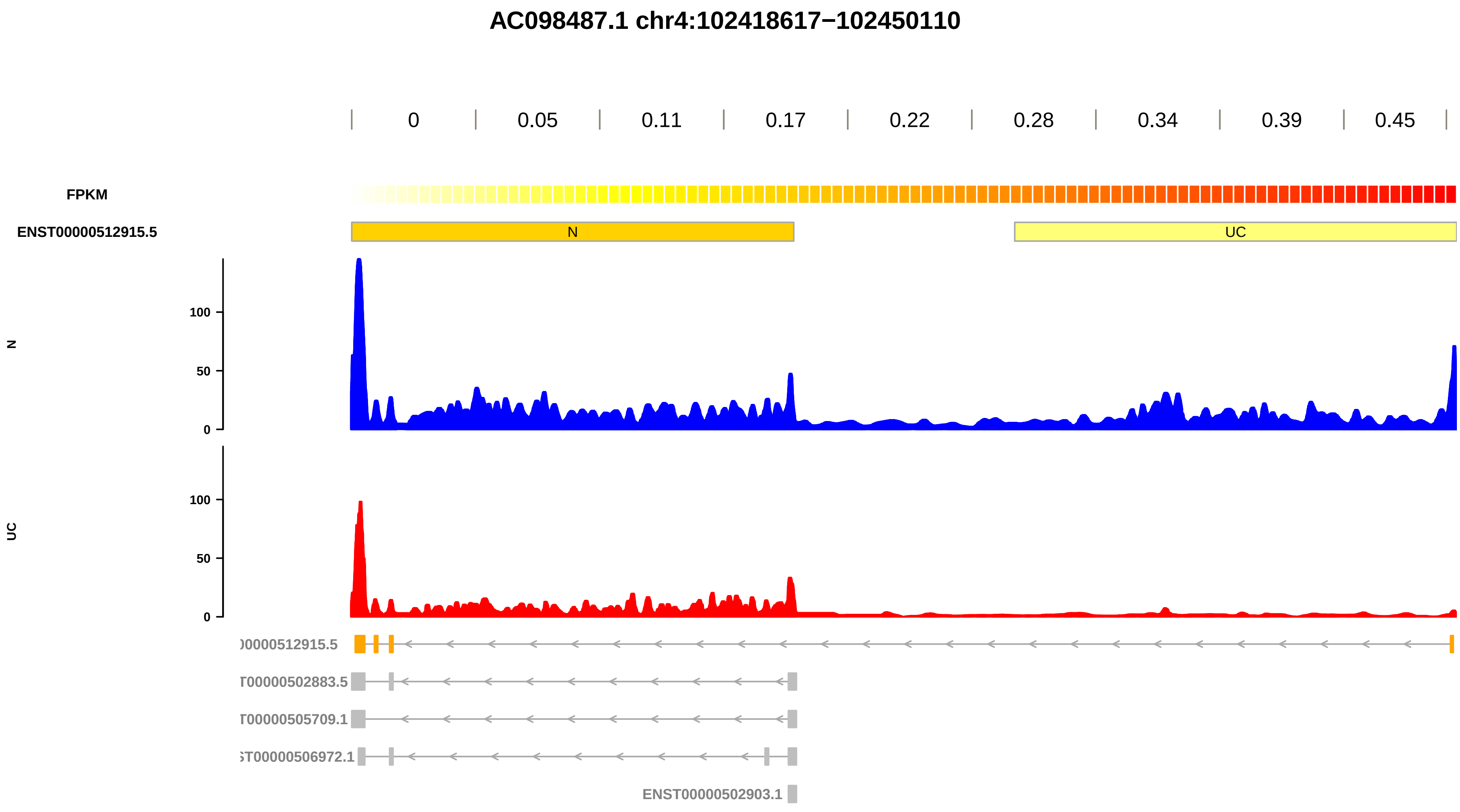

AC098487.1 chr4:102418617−102450110
0
0.05
0.11
0.17
0.22
0.28
0.34
0.39
0.45
FPKM
ENST00000512915.5
N
UC
N
UC
100
50
0
100
50
0
ENST00000502903.1

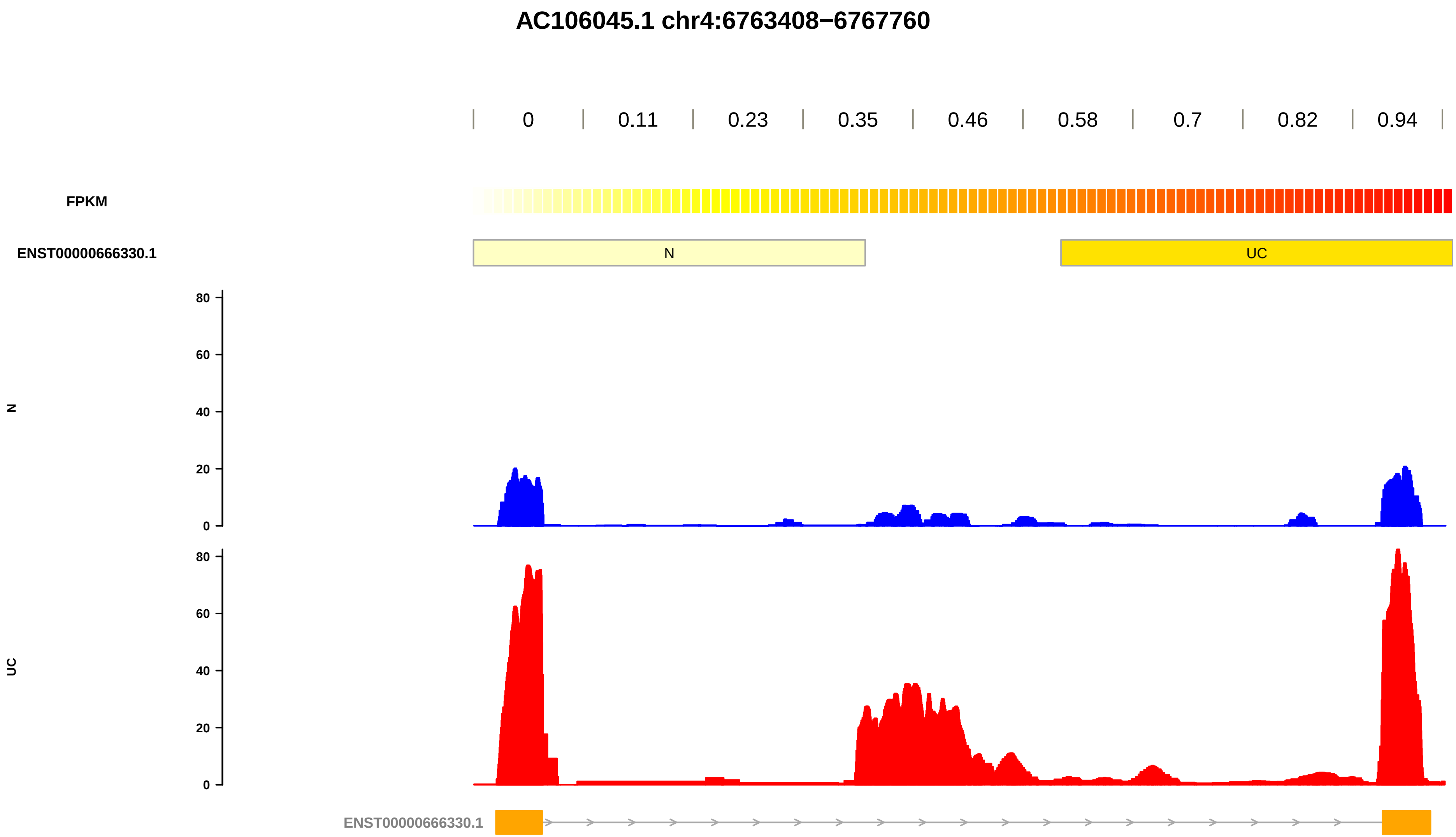

AC106045.1 chr4:6763408−6767760
0
0.11
0.23
0.35
0.46
0.58
0.7
0.82
0.94
FPKM
ENST00000666330.1
N
UC
N
80
60
40
20
0
UC
80
60
40
20
0
ENST00000666330.1

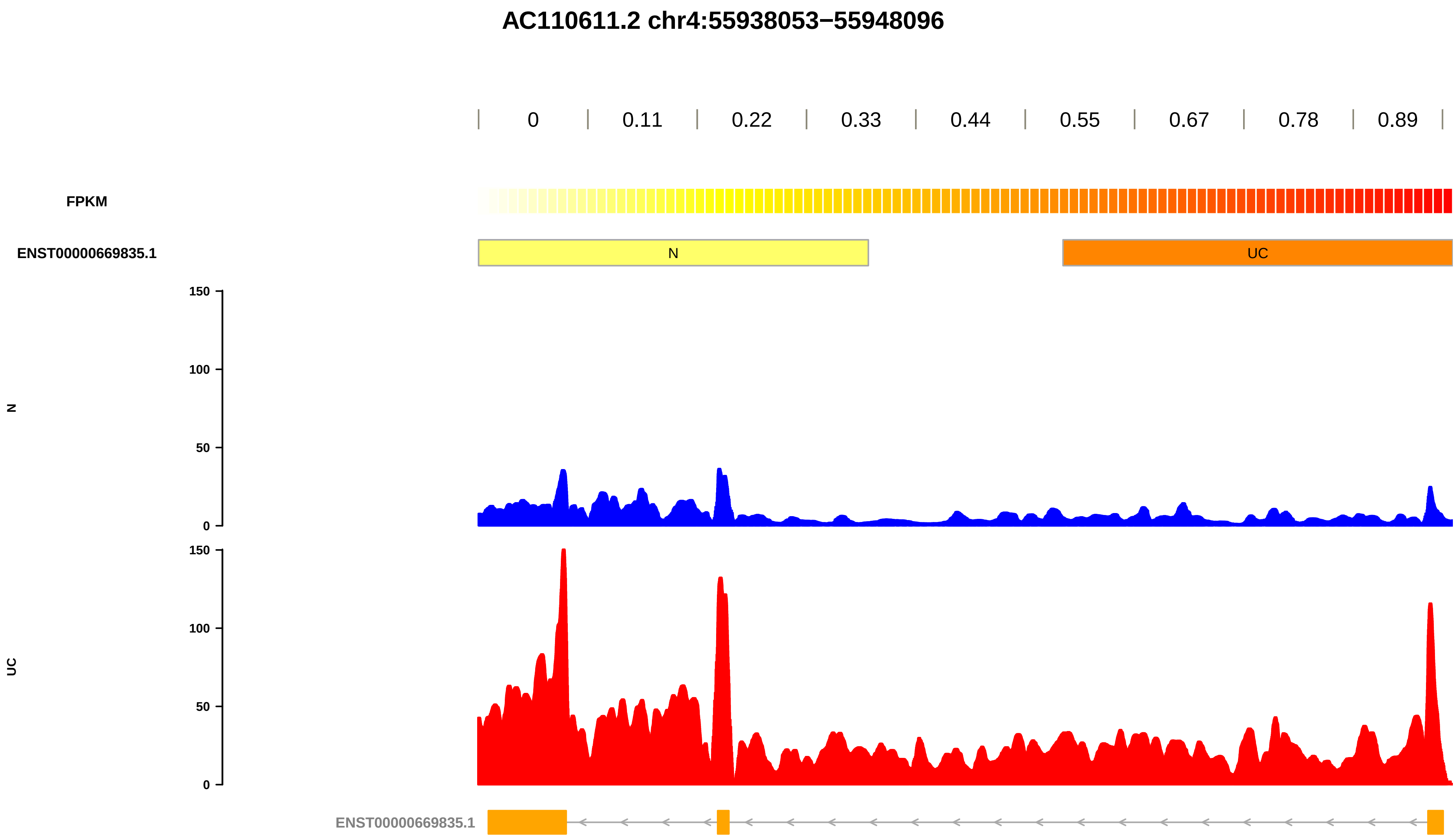

AC110611.2 chr4:55938053−55948096
0
0.11
0.22
0.33
0.44
0.55
0.67
0.78
0.89
FPKM
ENST00000669835.1
N
UC
150
100
50
0
N
150
100
50
0
UC
ENST00000669835.1

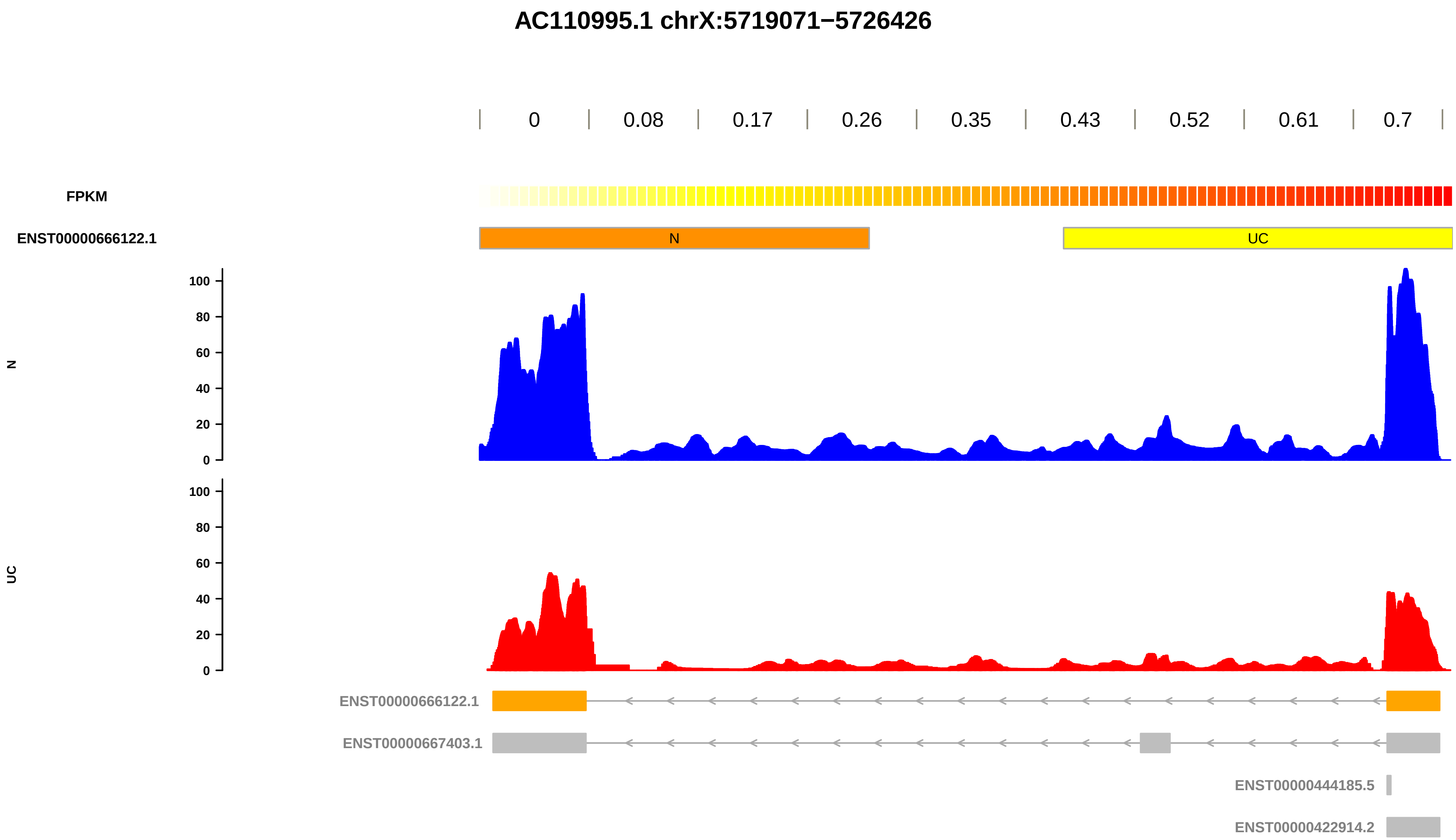
AC110995.1 chrX:5719071−5726426
0
0.08
0.17
0.26
0.35
0.43
0.52
0.61
0.7
FPKM
ENST00000666122.1
N
UC
100
80
60
40
20
0
N
UC
ENST00000666122.1
ENST00000667403.1
ENST00000444185.5
ENST00000422914.2

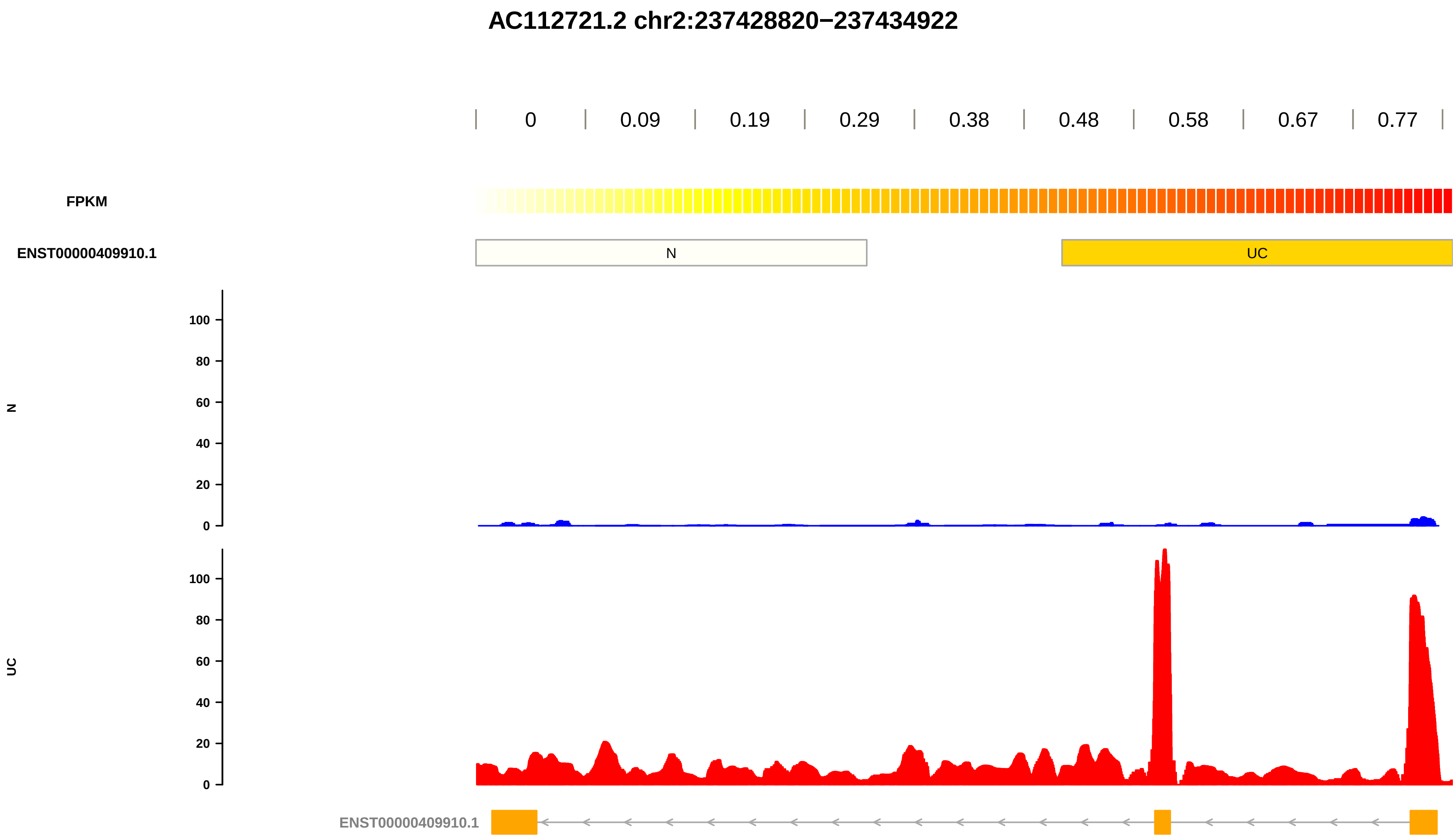

AC112721.2 chr2:237428820−237434922
0
0.09
0.19
0.29
0.38
0.48
0.58
0.67
0.77
FPKM
ENST00000409910.1
N
UC
100
80
60
40
20
0
N
UC
ENST00000409910.1

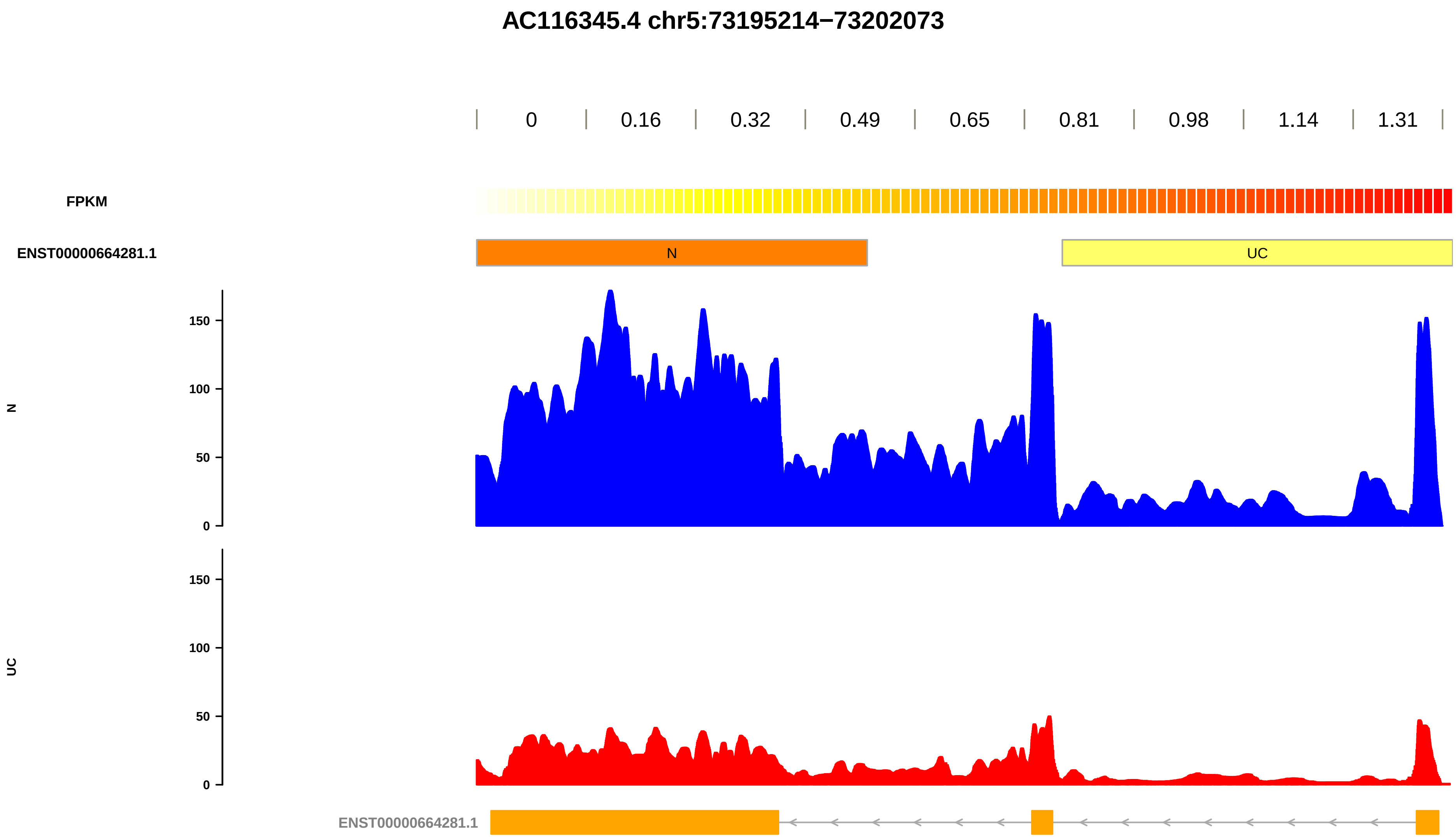
AC116345.4 chr5:73195214−73202073
0
0.16
0.32
0.49
0.65
0.81
0.98
1.14
1.31
FPKM
ENST00000664281.1
N
UC
150
100
50
0
N
UC
ENST00000664281.1

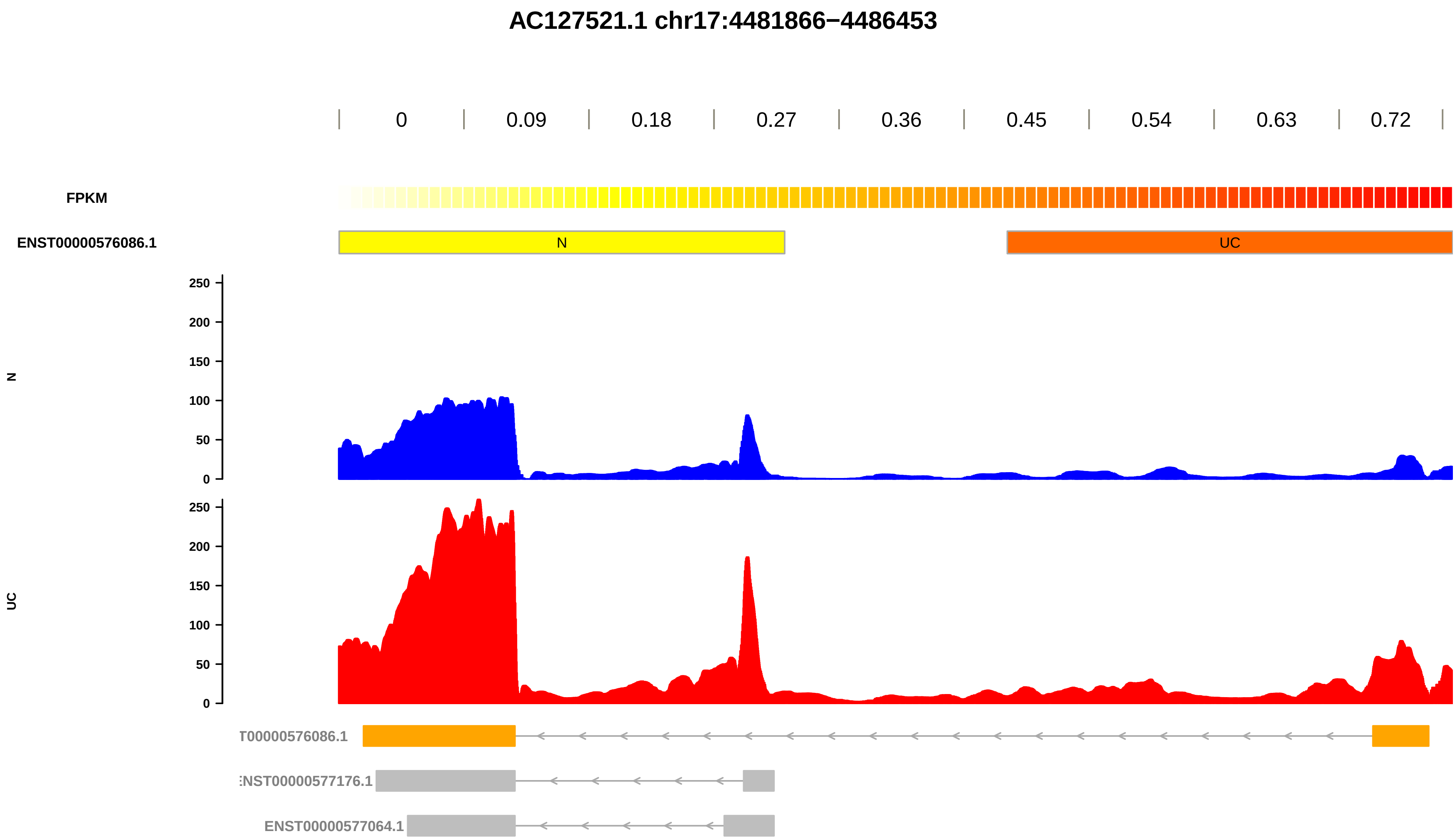

AC127521.1 chr17:4481866−4486453
0
0.09
0.18
0.27
0.36
0.45
0.54
0.63
0.72
FPKM
ENST00000576086.1
N
UC
250
200
150
100
50
0
N
UC
T00000576086.1
:NST00000577176.1
ENST00000577064.1

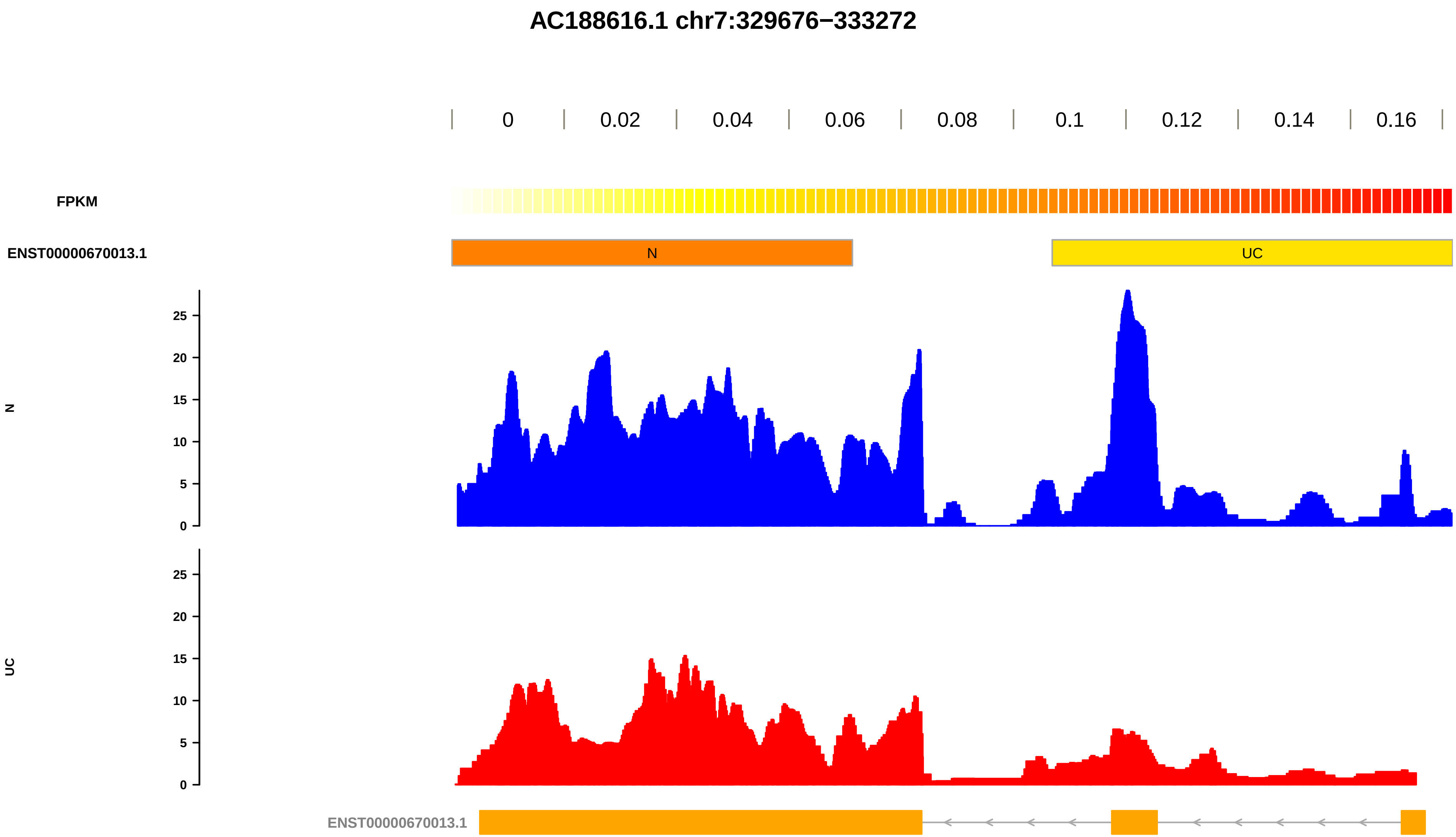
AC188616.1 chr7:329676−333272
0
0.02
0.04
0.06
0.08
0.1
0.12
0.14
0.16
FPKM
ENST00000670013.1
N
UC
N
UC
0
5
10
15
20
25
ENST00000670013.1

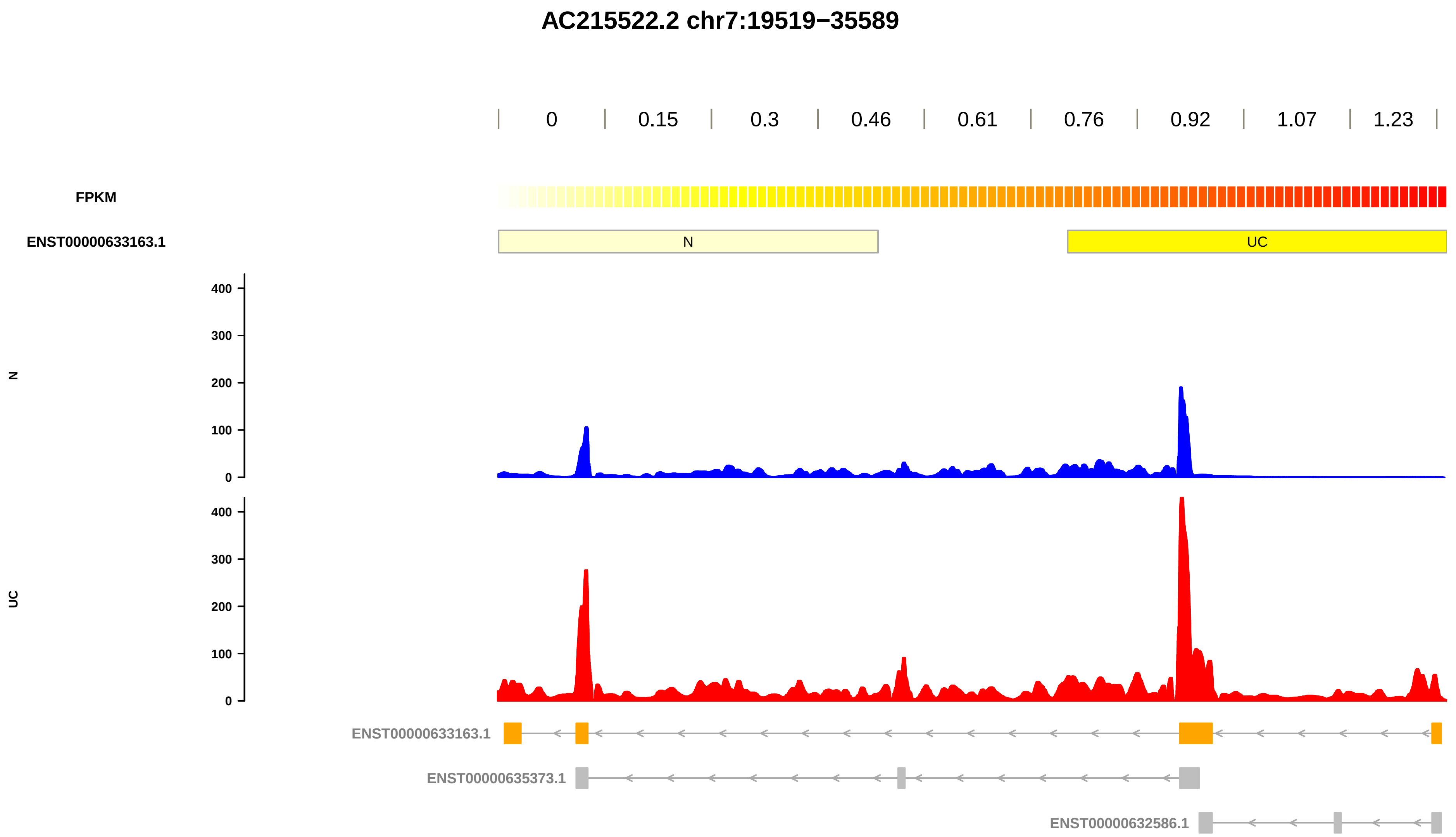

AC215522.2 chr7:19519−35589
0
0.15
0.3
0.46
0.61
0.76
0.92
1.07
1.23
FPKM
ENST00000633163.1
N
UC
400
300
200
100
0
N
400
300
200
100
0
UC
ENST00000633163.1
ENST00000635373.1
ENST00000632586.1

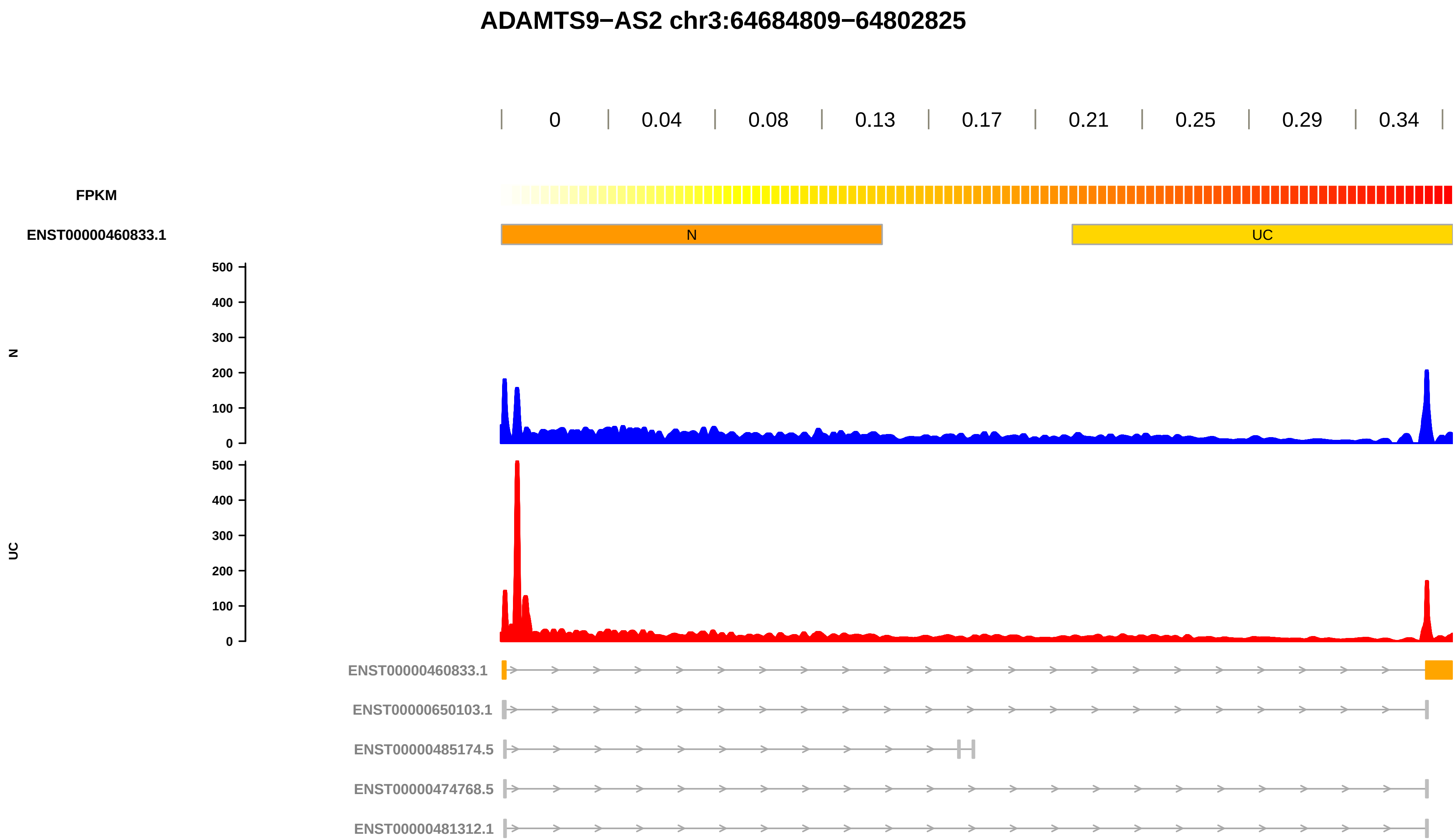
ADAMTS9−AS2 chr3:64684809−64802825
0
0.04
0.08
0.13
0.17
0.21
0.25
0.29
0.34
FPKM
ENST00000460833.1
N
UC
500
400
300
200
100
0
N
UC
ENST00000460833.1
ENST00000650103.1
ENST00000485174.5
ENST00000474768.5
ENST00000481312.1

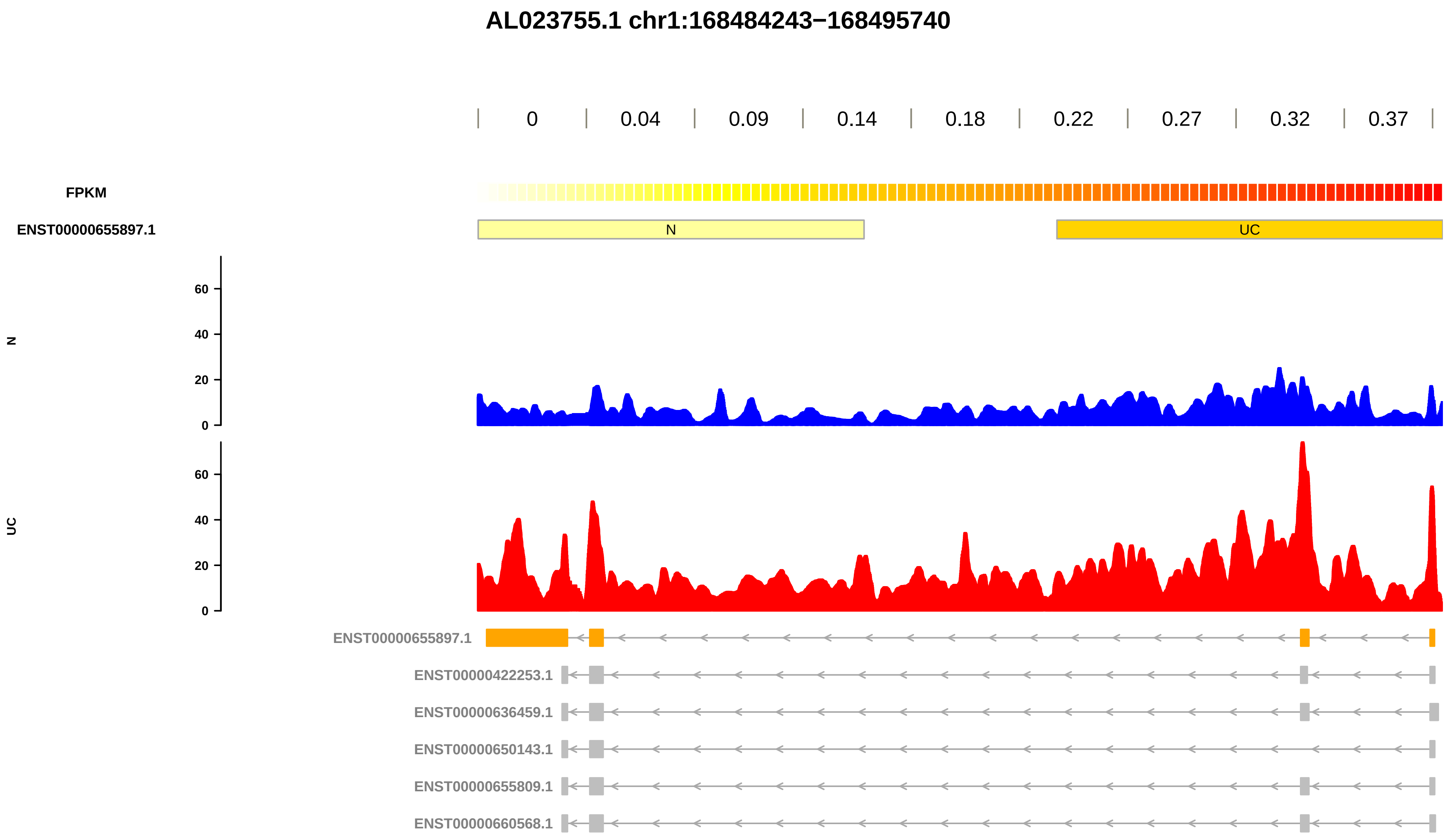

AL023755.1 chr1:168484243−168495740
0
0.04
0.09
0.14
0.18
0.22
0.27
0.32
0.37
FPKM
ENST00000655897.1
N
UC
N
UC
0
20
40
60
ENST00000655897.1
ENST00000422253.1
ENST00000636459.1
ENST00000650143.1
ENST00000655809.1
ENST00000660568.1

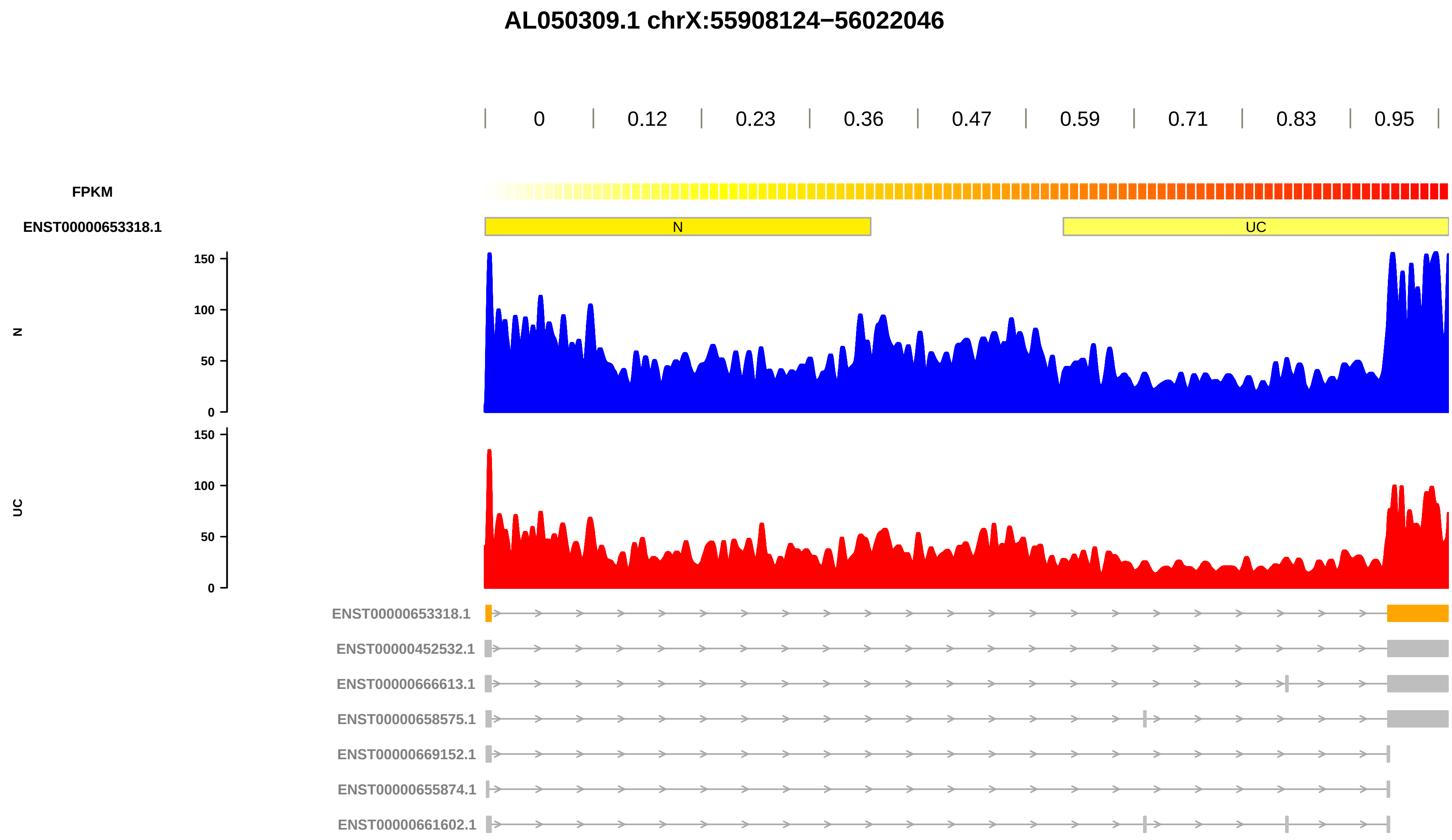
AL050309.1 chrX:55908124−56022046
0
0.12
0.23
0.36
0.47
0.59
0.71
0.83
0.95
FPKM
ENST00000653318.1
N
UC
150
100
50
0
N
150
100
50
0
UC
ENST00000653318.1
ENST00000452532.1
ENST00000666613.1
ENST00000658575.1
ENST00000669152.1
ENST00000655874.1
ENST00000661602.1

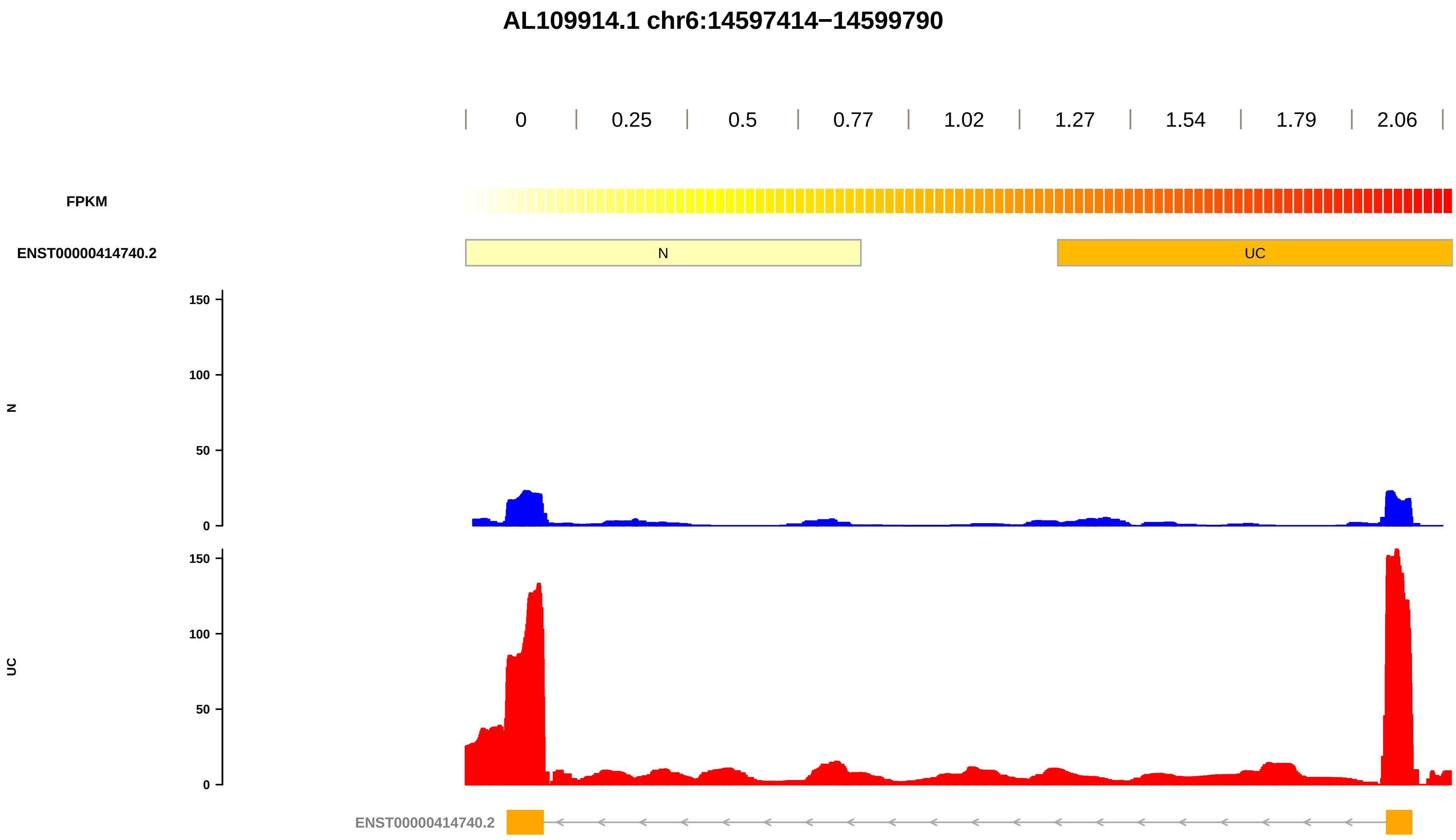
AL109914.1 chr6:14597414−14599790
0
0.25
0.5
0.77
1.02
1.27
1.54
1.79
2.06
FPKM
ENST00000414740.2
N
UC
150
100
50
0
N
150
100
50
0
UC
ENST00000414740.2

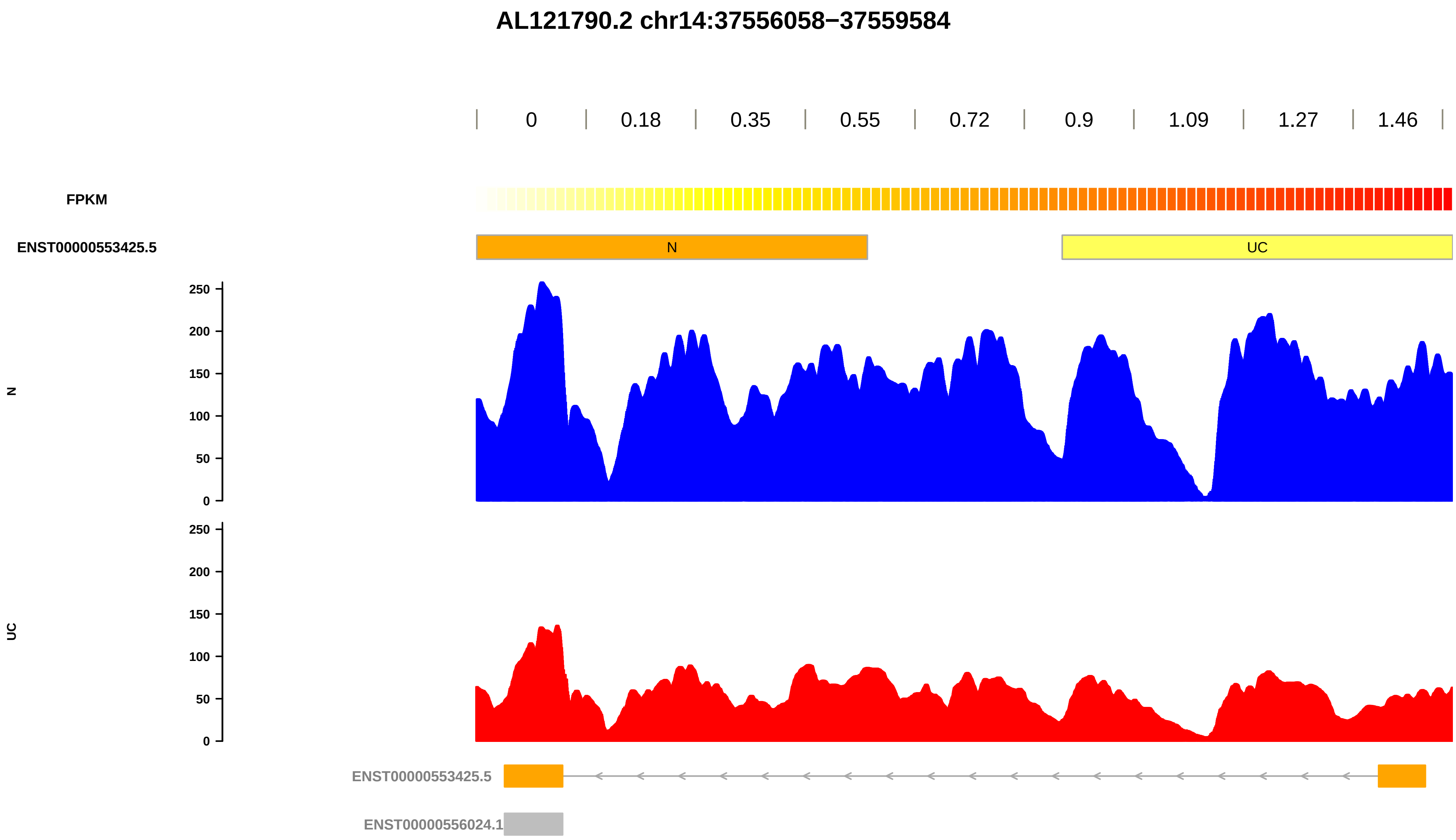
AL121790.2 chr14:37556058−37559584
0
0.18
0.35
0.55
0.72
0.9
1.09
1.27
1.46
FPKM
ENST00000553425.5
N
UC
250
200
150
100
50
0
N
UC
ENST00000553425.5
ENST00000556024.1

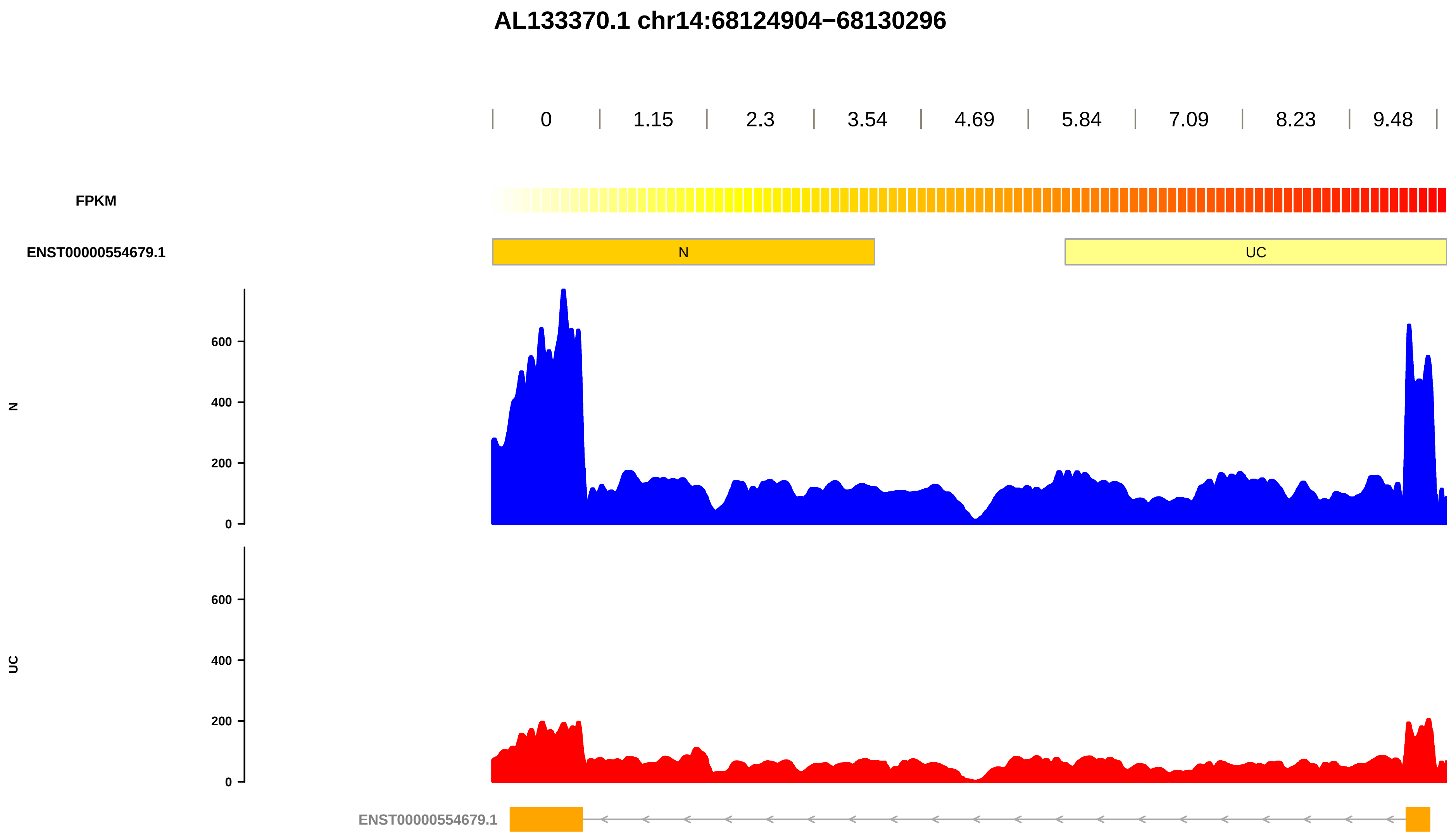
AL133370.1 chr14:68124904−68130296
0
1.15
2.3
3.54
4.69
5.84
7.09
8.23
9.48
FPKM
ENST00000554679.1
N
UC
N
600
400
200
0
UC
600
400
200
0
ENST00000554679.1

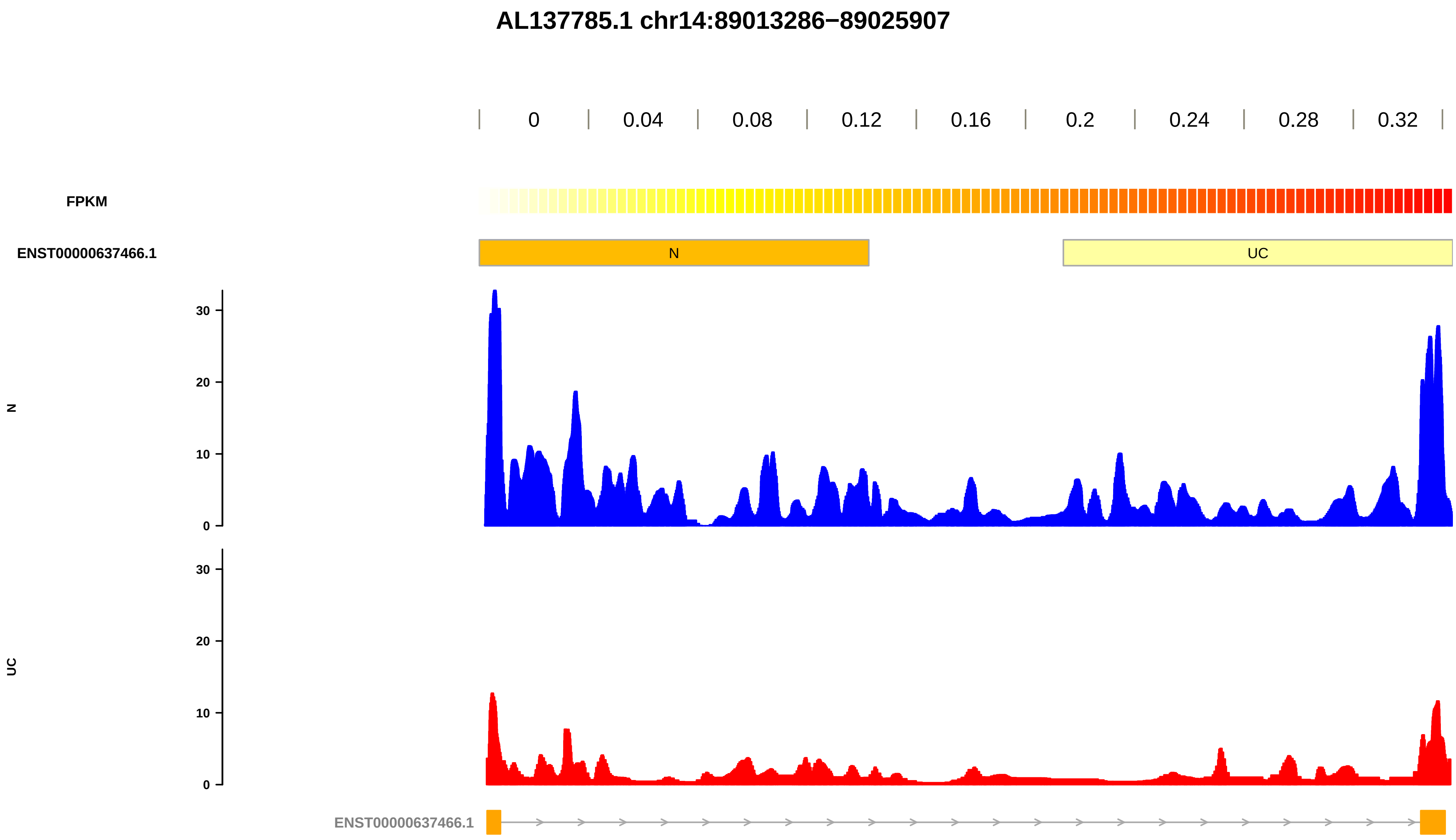

AL137785.1 chr14:89013286−89025907
0
0.04
0.08
0.12
0.16
0.2
0.24
0.28
0.32
FPKM
ENST00000637466.1
N
UC
N
30
20
10
0
UC
30
20
10
0
ENST00000637466.1

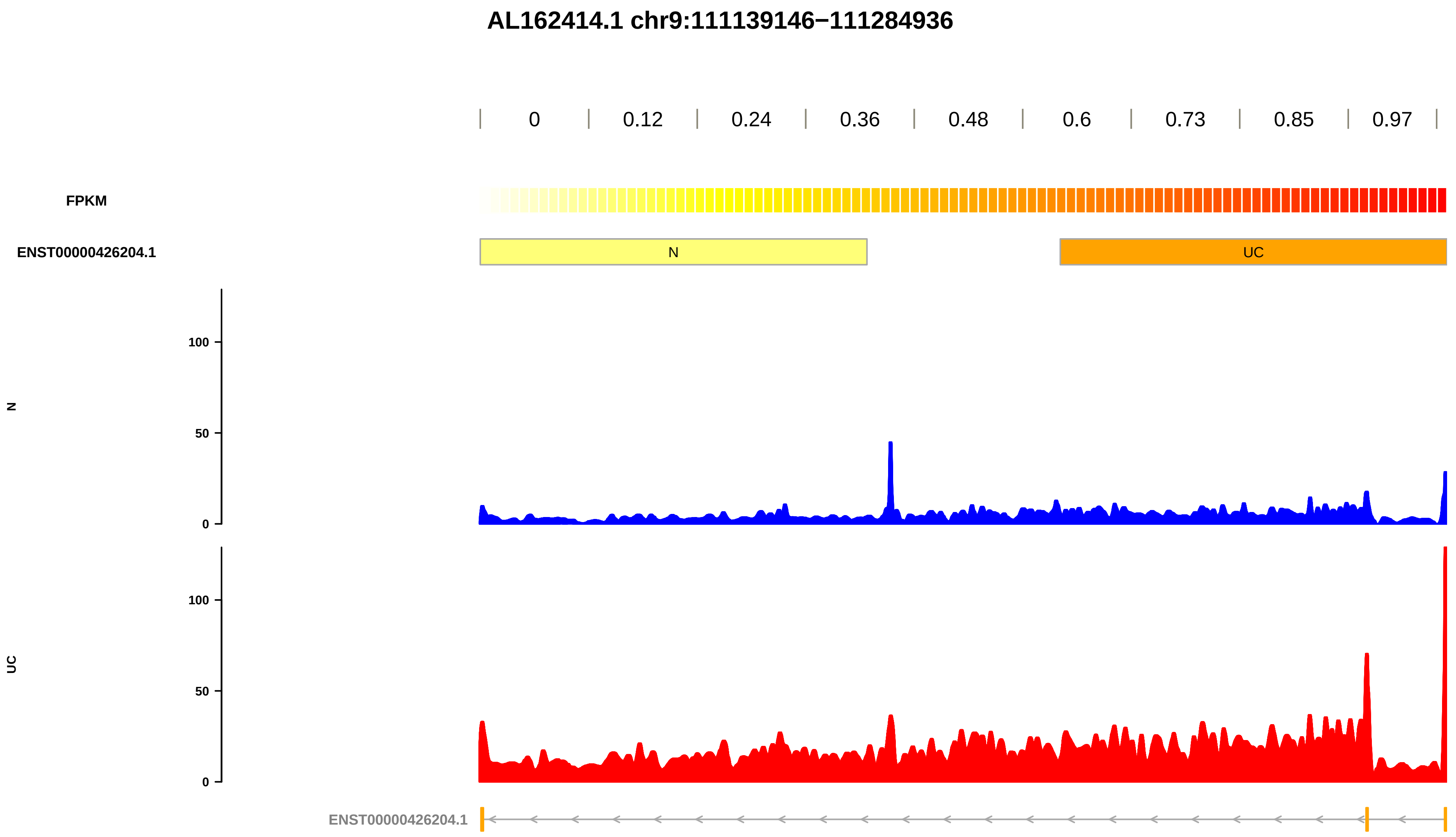
AL162414.1 chr9:111139146−111284936
0
0.12
0.24
0.36
0.48
0.6
0.73
0.85
0.97
FPKM
ENST00000426204.1
N
UC
100
50
0
N
100
50
0
UC
ENST00000426204.1

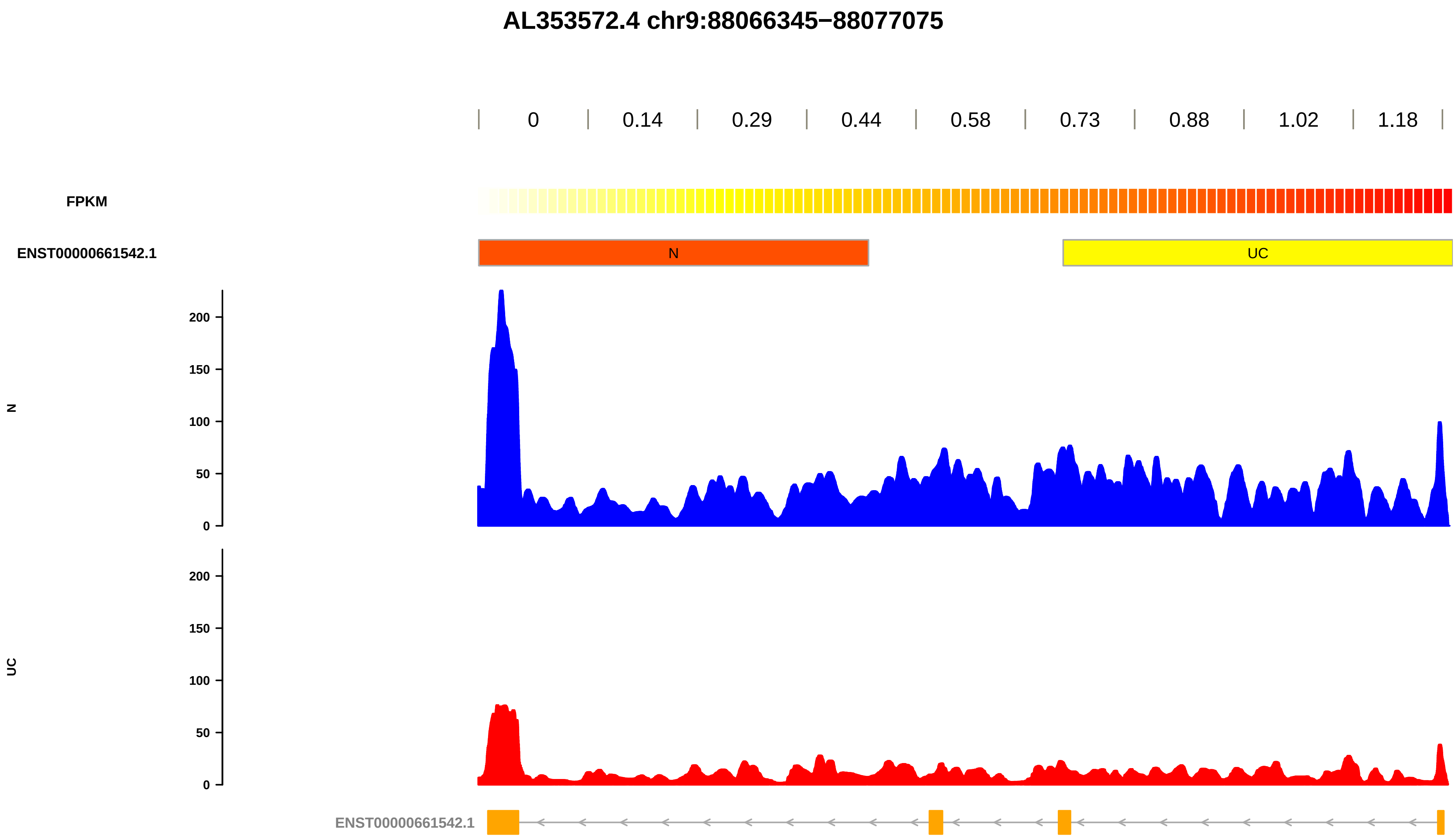

AL353572.4 chr9:88066345−88077075
0
0.14
0.29
0.44
0.58
0.73
0.88
1.02
1.18
FPKM
ENST00000661542.1
N
UC
200
150
100
50
0
N
UC
ENST00000661542.1

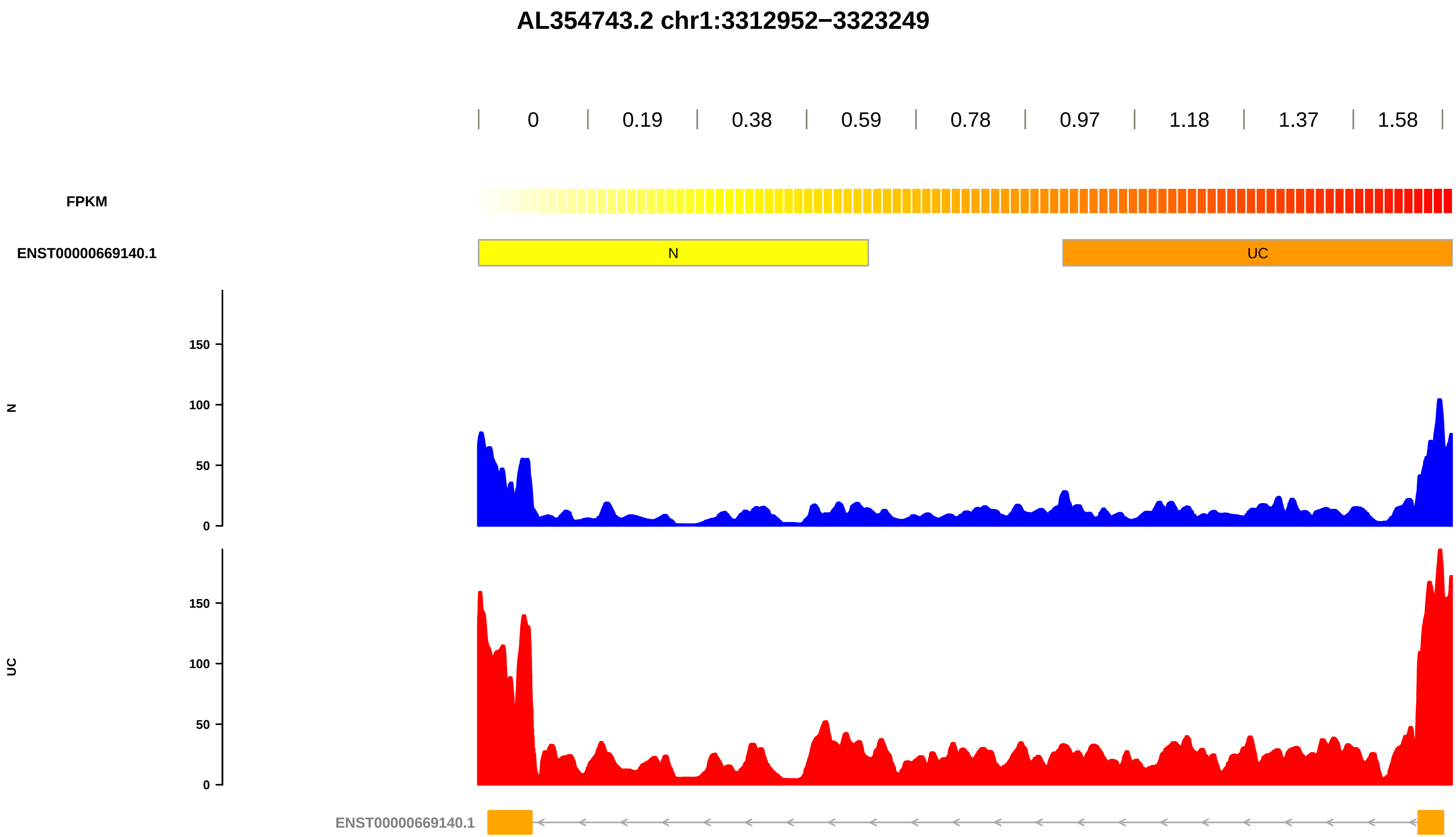
AL354743.2 chr1:3312952−3323249
0
0.19
0.38
0.59
0.78
0.97
1.18
1.37
1.58
FPKM
ENST00000669140.1
N
UC
N
150
100
50
0
UC
150
100
50
0
ENST00000669140.1

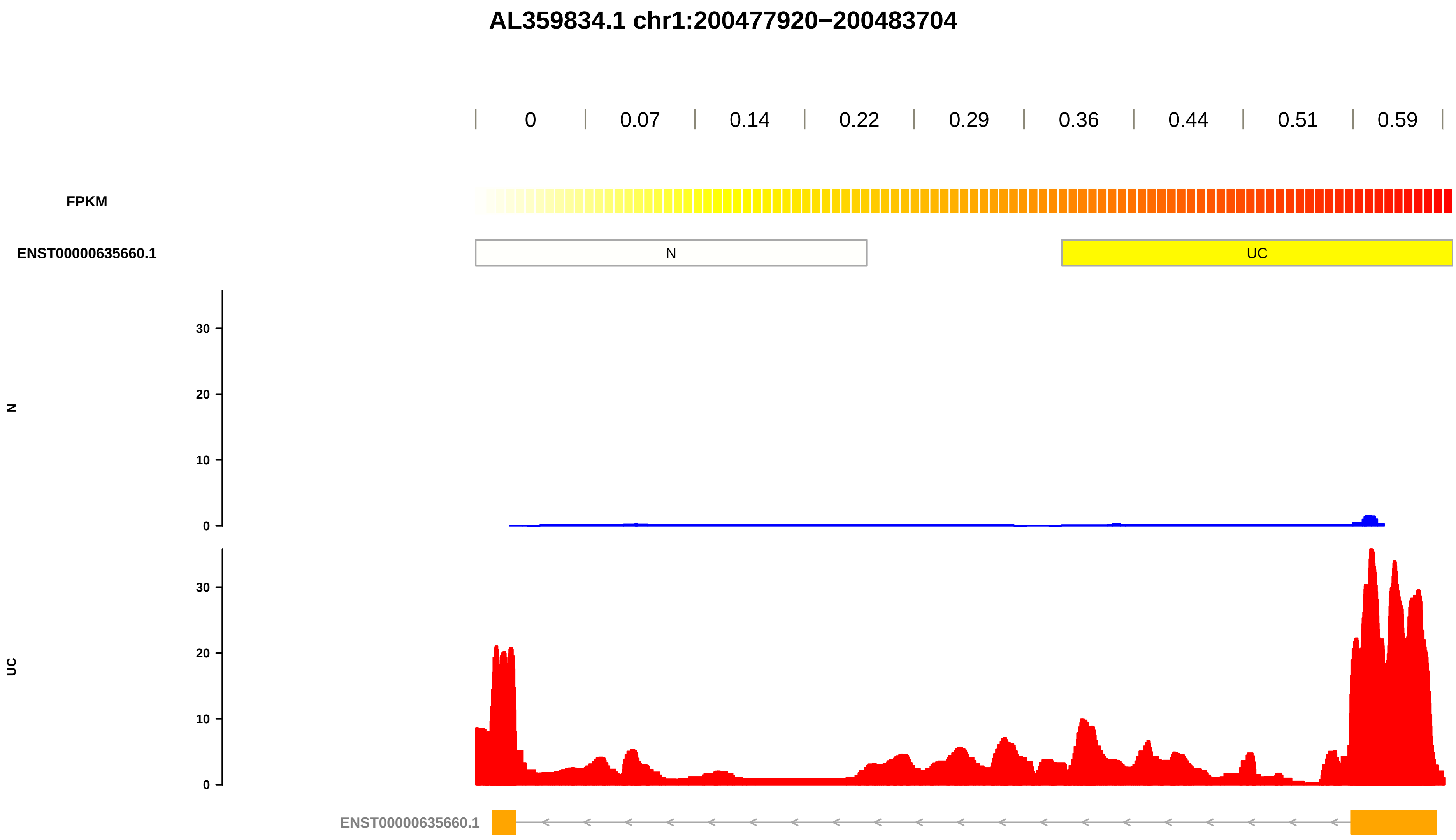

AL359834.1 chr1:200477920−200483704
0
0.07
0.14
0.22
0.29
0.36
0.44
0.51
0.59
FPKM
ENST00000635660.1
N
UC
N
30
20
10
0
UC
30
20
10
0
ENST00000635660.1

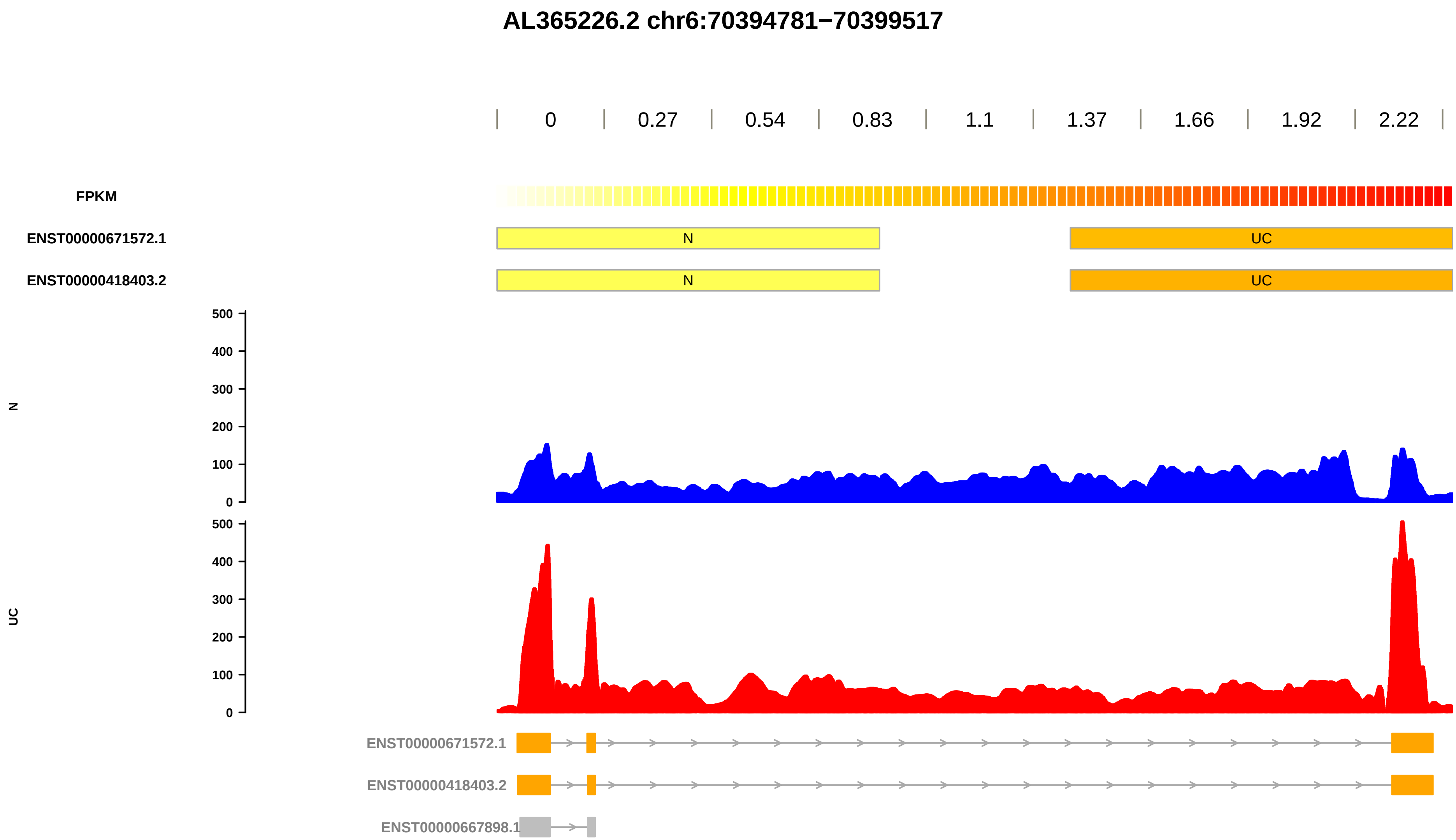
AL365226.2 chr6:70394781−70399517
0
0.27
0.54
0.83
1.1
1.37
1.66
1.92
2.22
FPKM
ENST00000671572.1
ENST00000418403.2
N
UC
N
UC
500
400
300
200
100
0
ENST00000671572.1
ENST00000418403.2
ENST00000667898.1

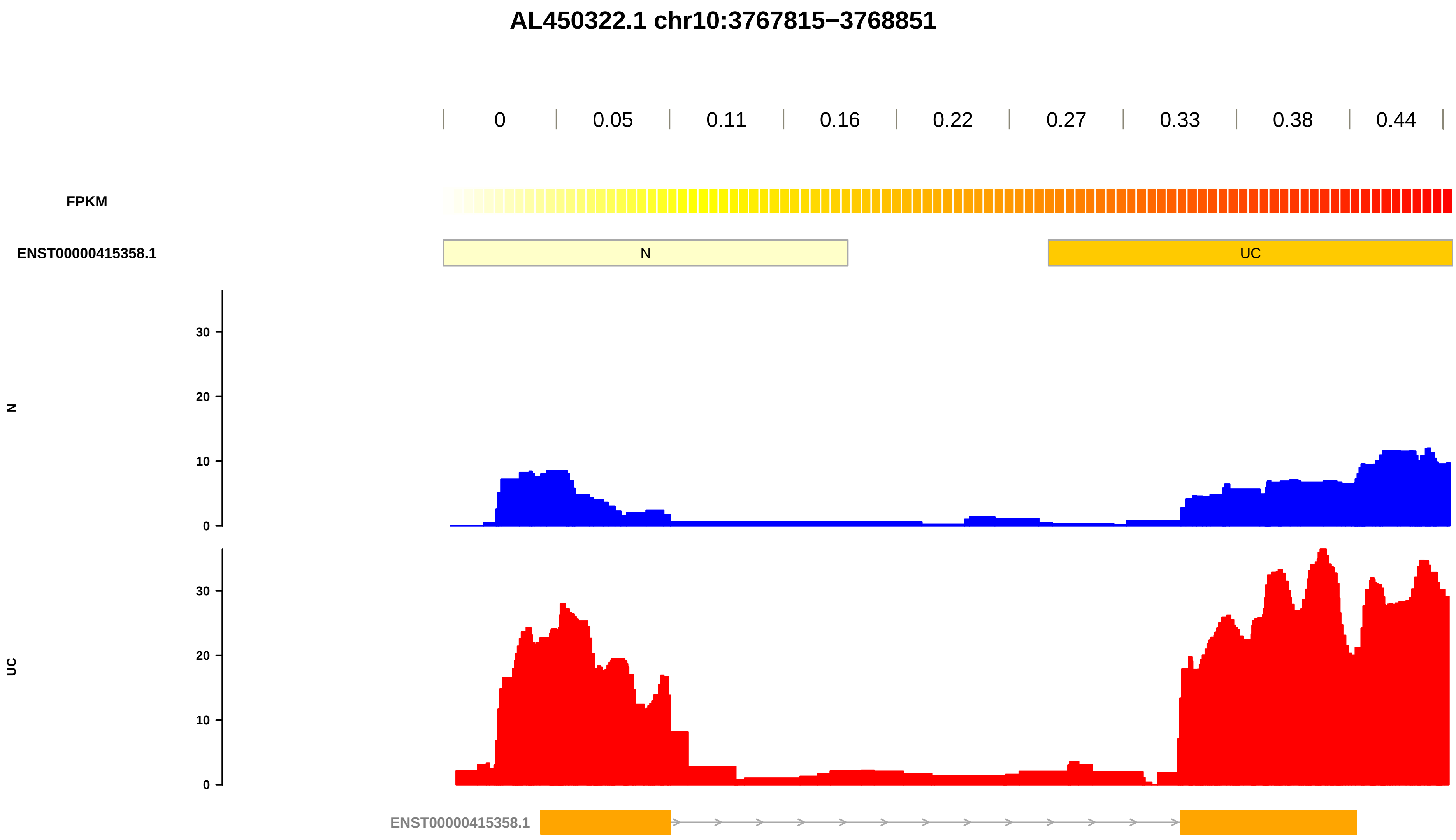

AL450322.1 chr10:3767815−3768851
0
0.05
0.11
0.16
0.22
0.27
0.33
0.38
0.44
FPKM
ENST00000415358.1
N
UC
N
UC
0
10
20
30
ENST00000415358.1

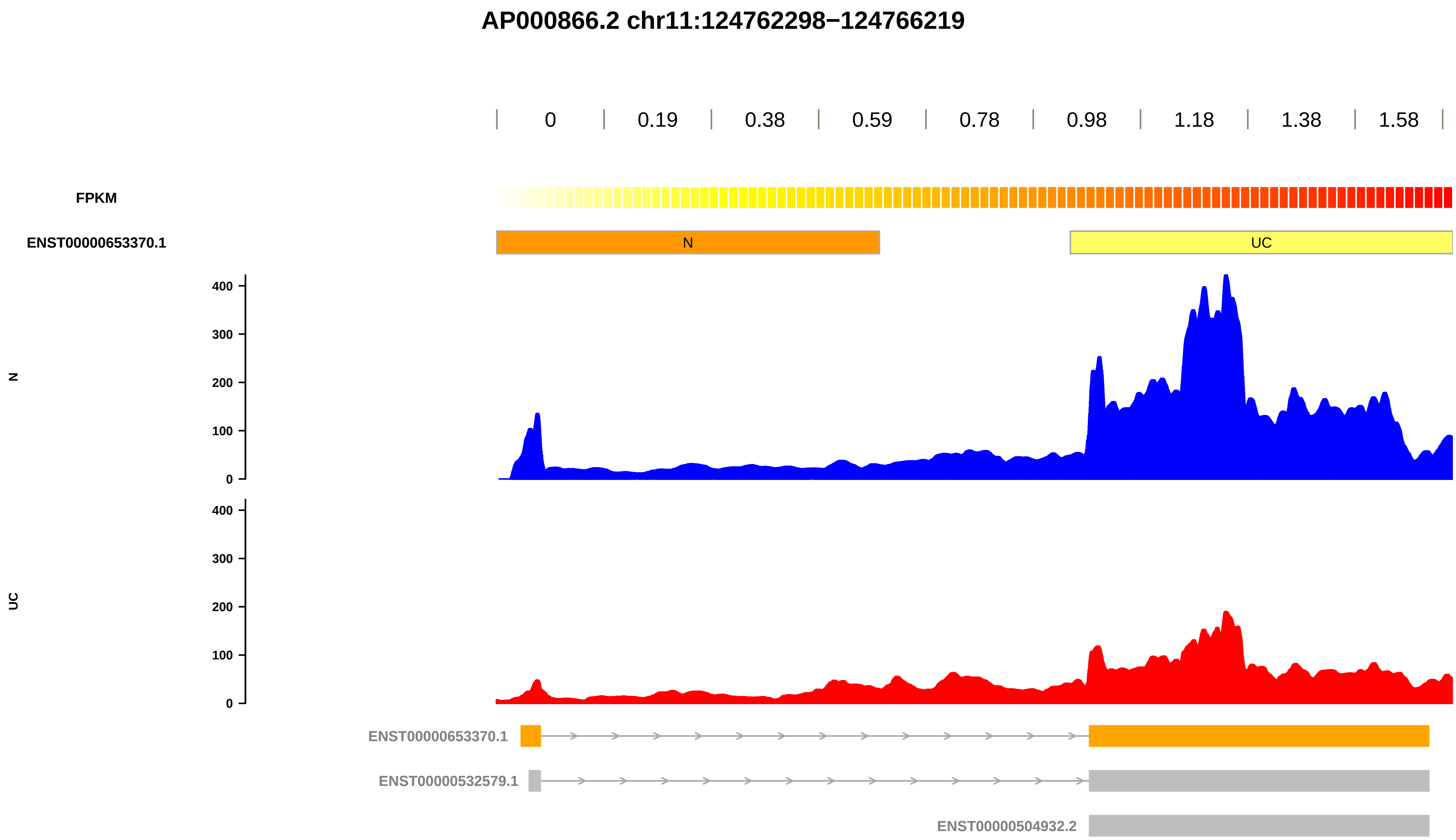
AP000866.2 chr11:124762298−124766219
0
0.19
0.38
0.59
0.78
0.98
1.18
1.38
1.58
FPKM
ENST00000653370.1
N
UC
400
300
200
100
0
N
400
300
200
100
0
UC
ENST00000653370.1
ENST00000532579.1
ENST00000504932.2

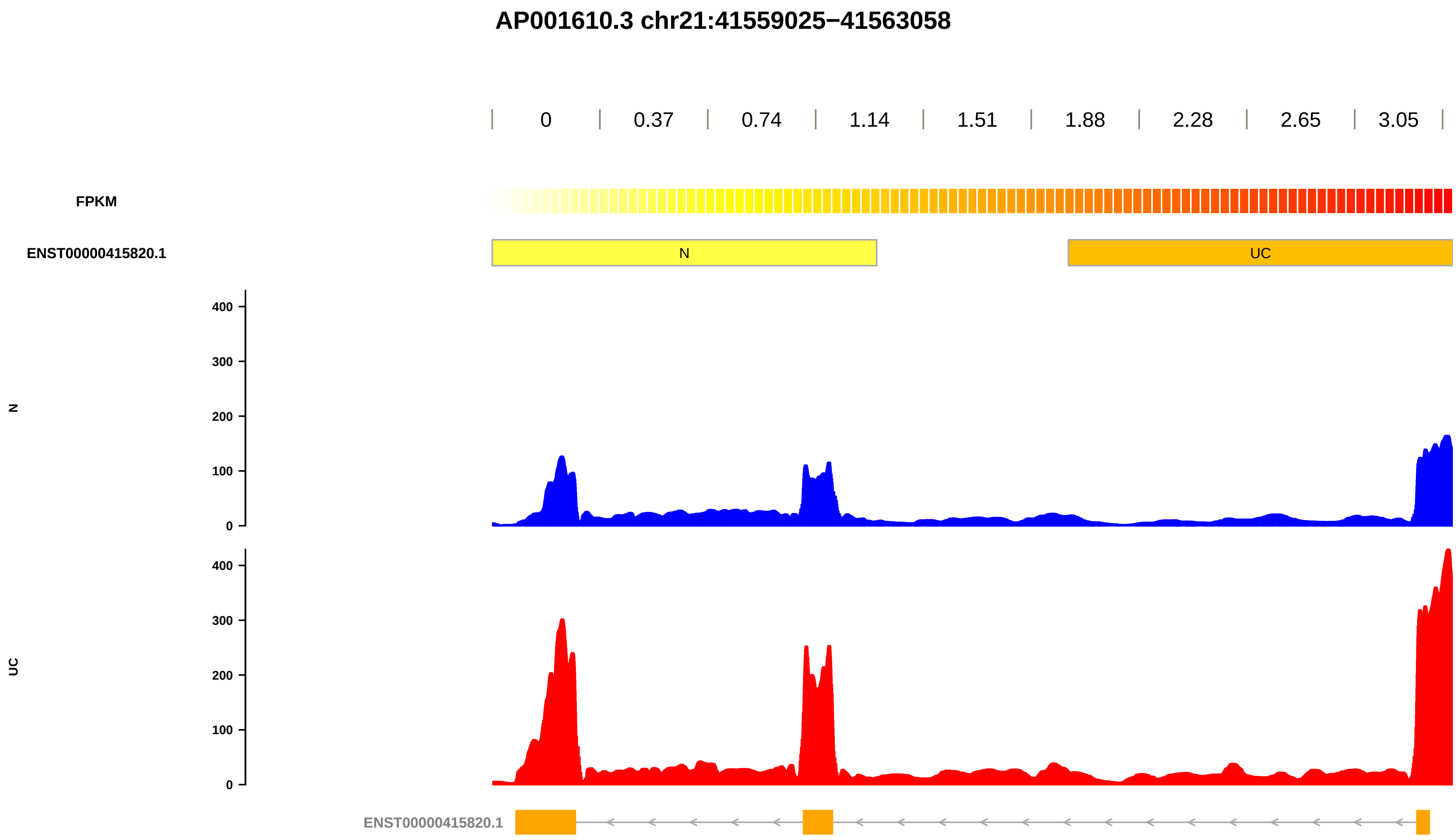
AP001610.3 chr21:41559025−41563058
0
0.37
0.74
1.14
1.51
1.88
2.28
2.65
3.05
FPKM
ENST00000415820.1
N
UC
N
400
300
200
100
0
UC
400
300
200
100
0
ENST00000415820.1

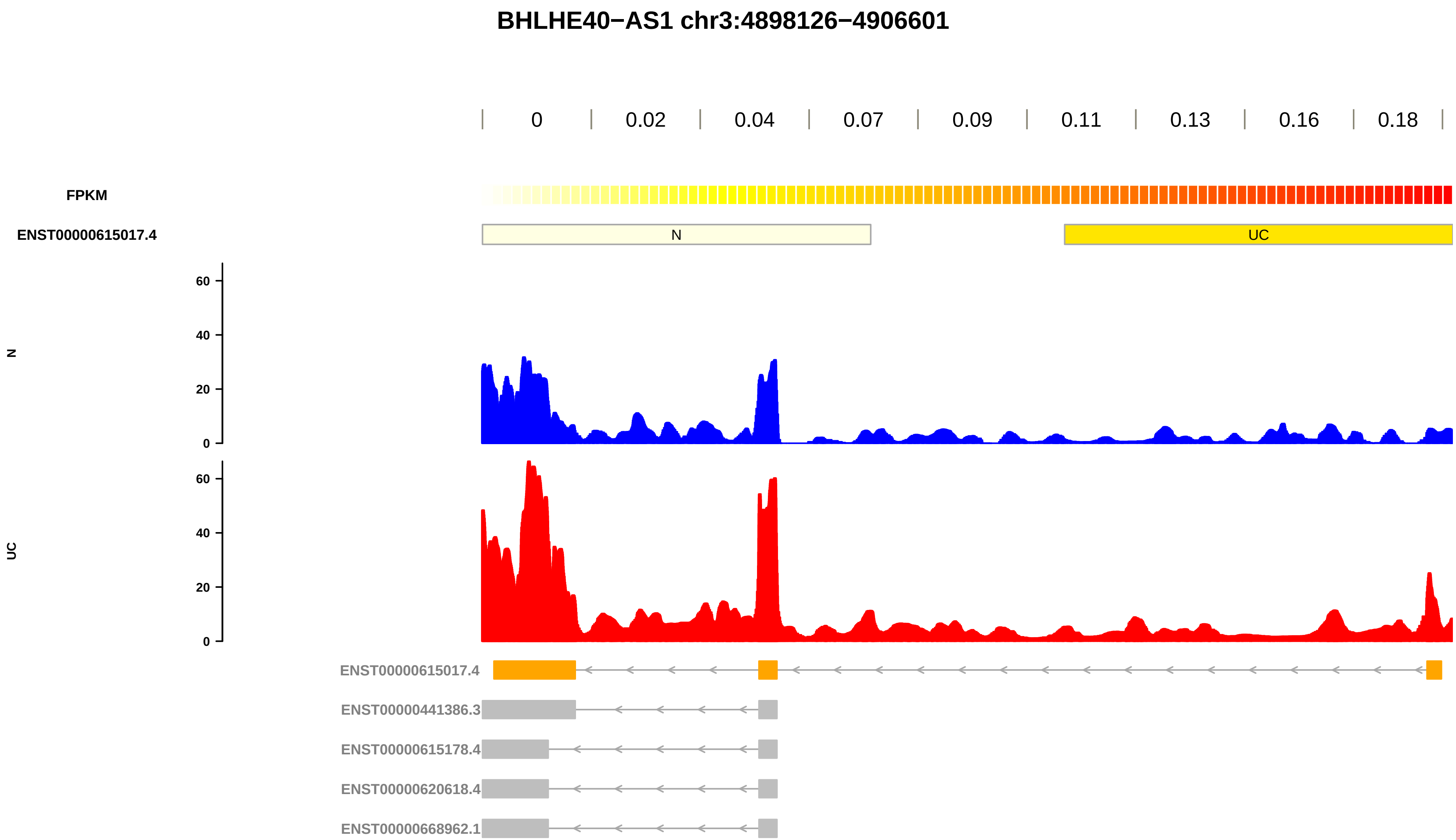
BHLHE40−AS1 chr3:4898126−4906601
0
0.02
0.04
0.07
0.09
0.11
0.13
0.16
0.18
FPKM
ENST00000615017.4
N
UC
60
40
20
0
N
60
40
20
0
UC
ENST00000615017.4
ENST00000441386.3
ENST00000615178.4
ENST00000620618.4
ENST00000668962.1

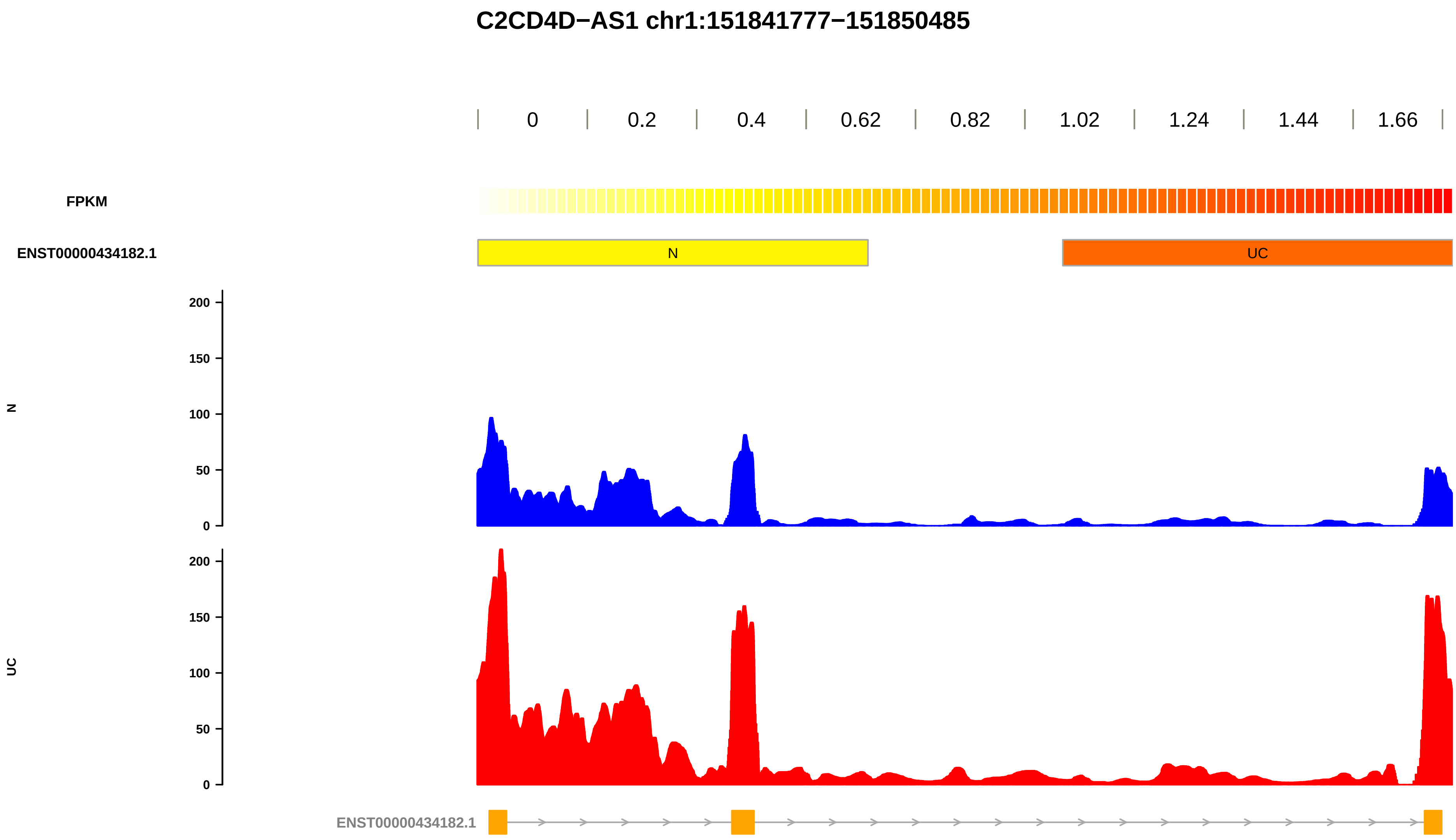

C2CD4D−AS1 chr1:151841777−151850485
0
0.2
0.4
0.62
0.82
1.02
1.24
1.44
1.66
FPKM
ENST00000434182.1
N
UC
200
150
100
50
0
N
200
150
100
50
0
UC
ENST00000434182.1

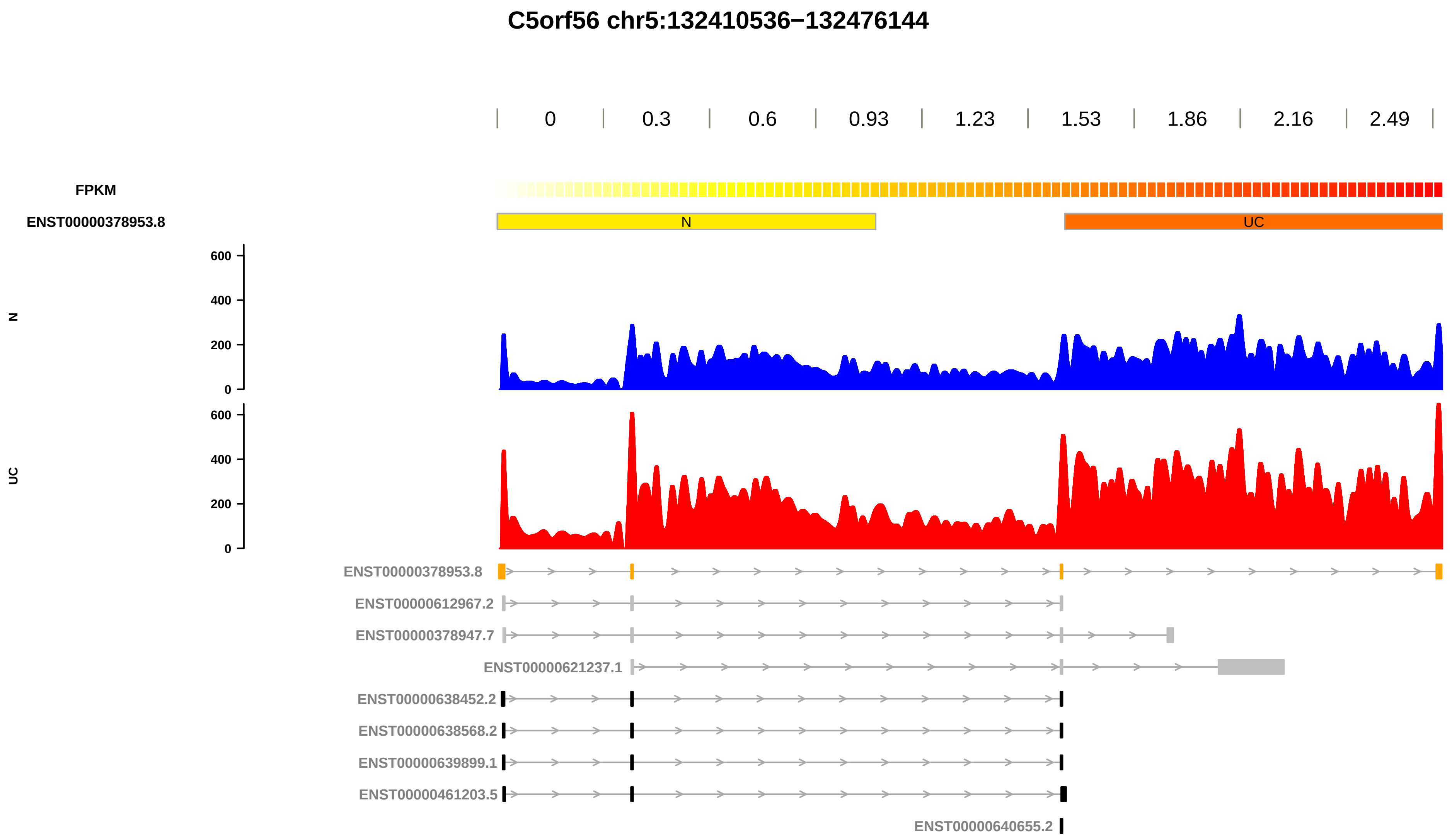

C5orf56 chr5:132410536−132476144
0
0.3
0.6
0.93
1.23
1.53
1.86
2.16
2.49
FPKM
ENST00000378953.8
N
UC
600
400
200
0
N
600
400
200
0
UC
ENST00000378953.8
ENST00000612967.2
ENST00000378947.7
ENST00000621237.1
ENST00000638452.2
ENST00000638568.2
ENST00000639899.1
ENST00000461203.5
ENST00000640655.2

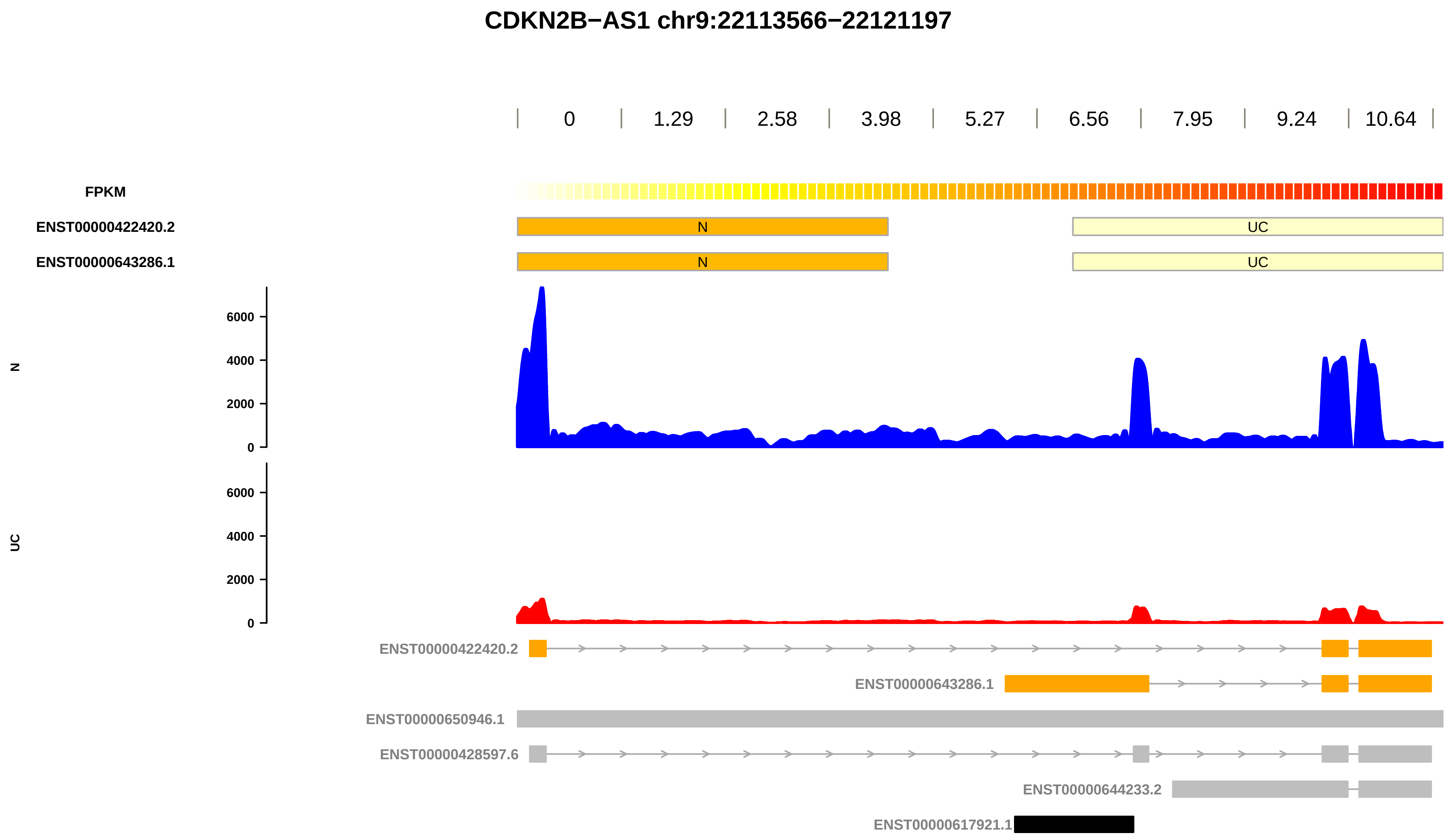

CDKN2B−AS1 chr9:22113566−22121197
0
1.29
2.58
3.98
5.27
6.56
7.95
9.24
10.64
FPKM
ENST00000422420.2
ENST00000643286.1
N
UC
N
UC
N
6000
4000
2000
0
UC
6000
4000
2000
0
ENST00000422420.2
ENST00000643286.1
ENST00000650946.1
ENST00000428597.6
ENST00000644233.2
ENST00000617921.1

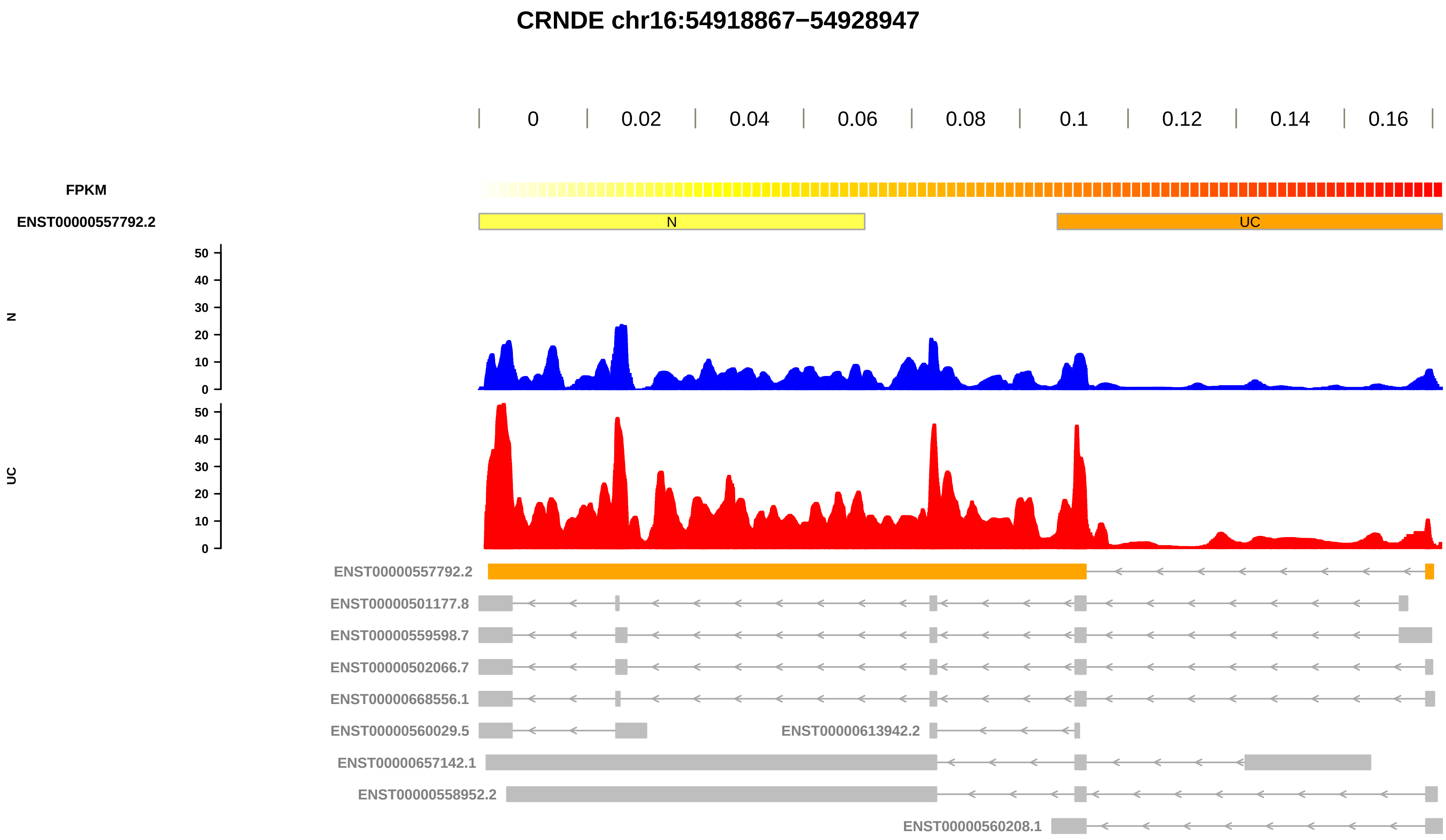
CRNDE chr16:54918867−54928947
0
0.02
0.04
0.06
0.08
0.1
0.12
0.14
0.16
FPKM
ENST00000557792.2
N
UC
50
40
30
20
10
0
N
UC
ENST00000557792.2
ENST00000501177.8
ENST00000559598.7
ENST00000502066.7
ENST00000668556.1
ENST00000560029.5
ENST00000613942.2
ENST00000657142.1
ENST00000558952.2
ENST00000560208.1

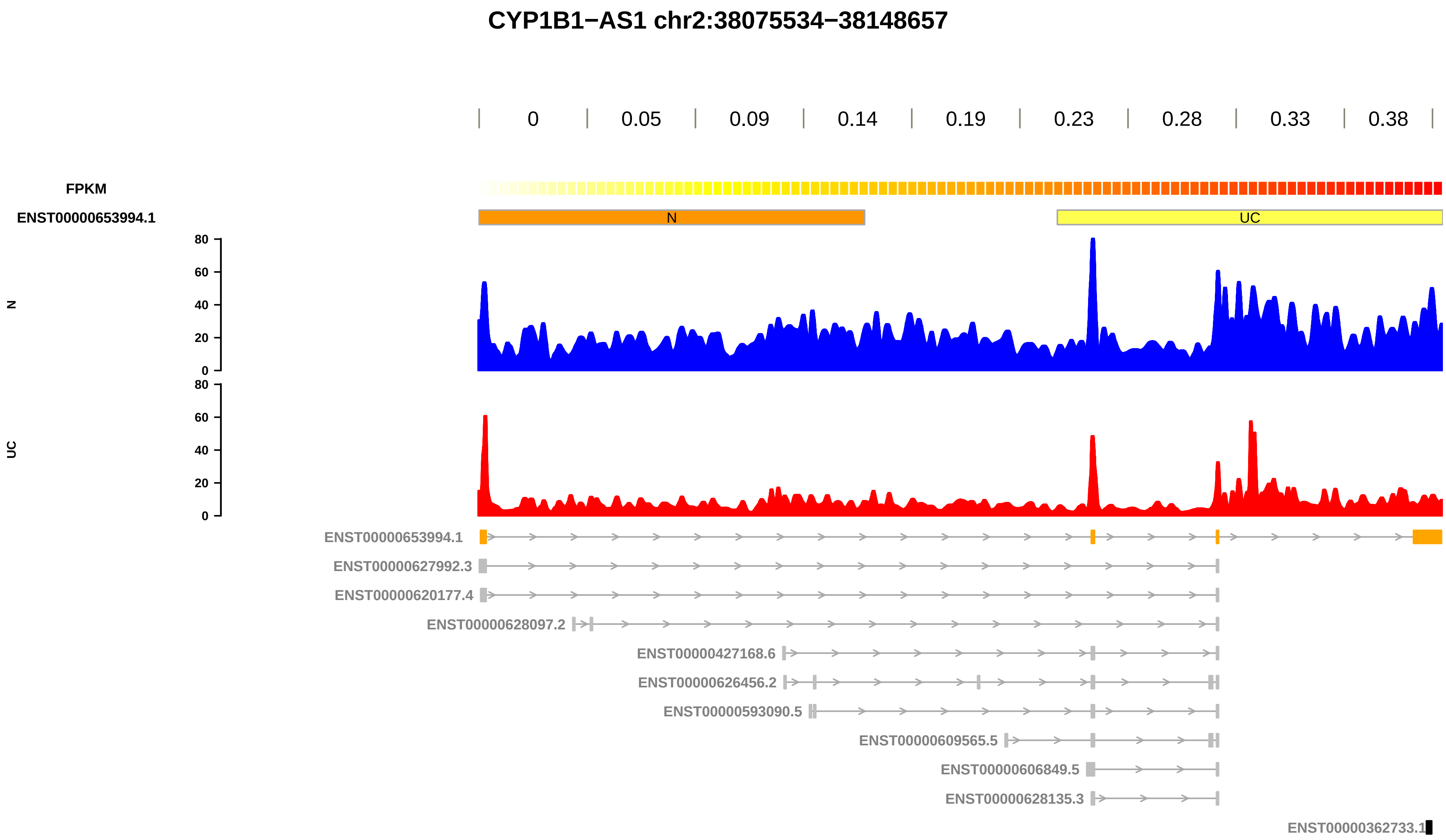
CYP1B1−AS1 chr2:38075534−38148657
0
0.05
0.09
0.14
0.19
0.23
0.28
0.33
0.38
FPKM
ENST00000653994.1
N
UC
80
60
40
20
0
ENST00000653994.1
ENST00000627992.3
ENST00000620177.4
ENST00000628097.2
ENST00000427168.6
ENST00000626456.2
ENST00000593090.5
ENST00000609565.5
ENST00000606849.5
ENST00000628135.3
ENST00000362733.1

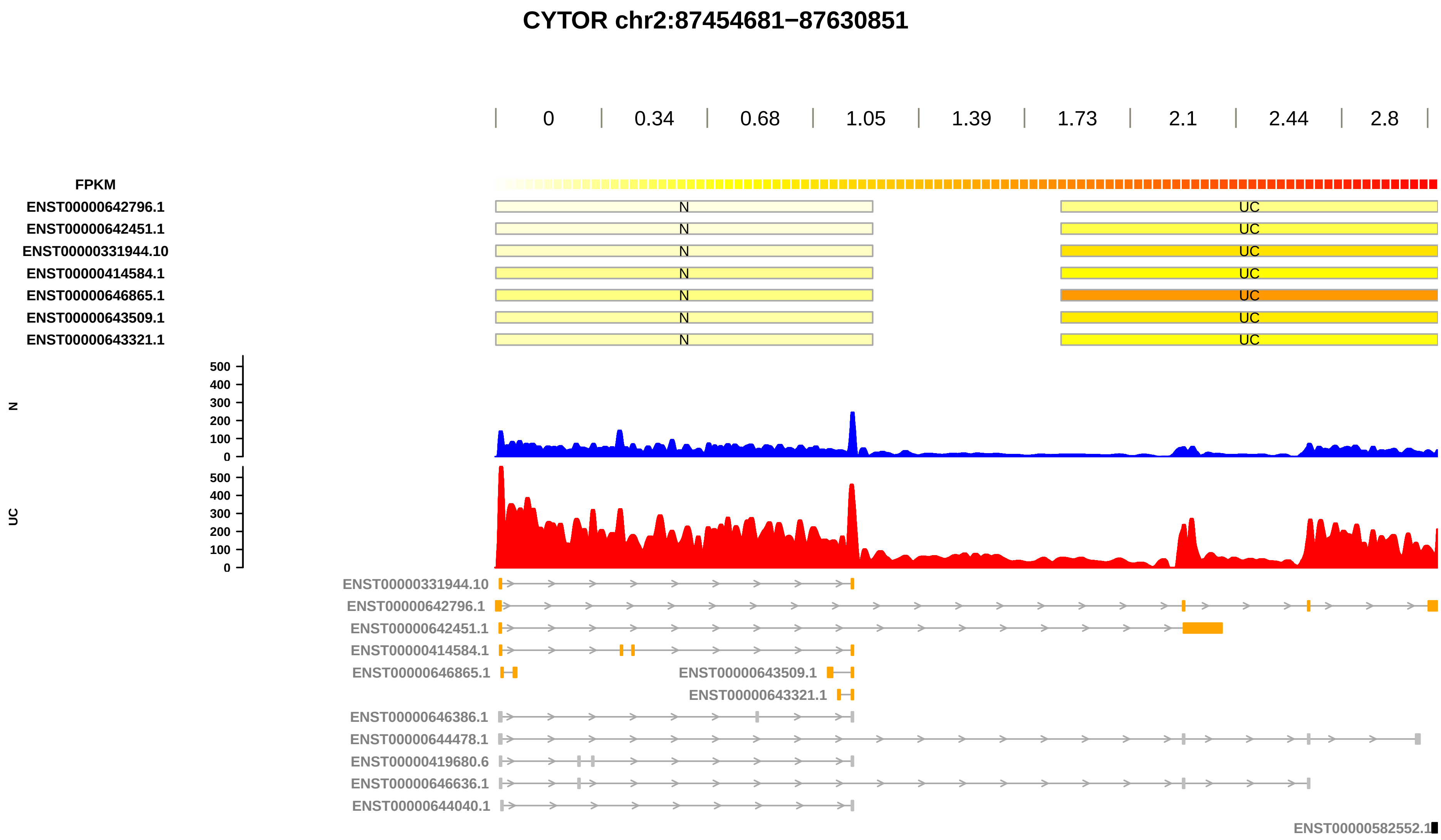
CYTOR chr2:87454681−87630851
0
0.34
0.68
1.05
1.39
1.73
2.1
2.44
2.8
FPKM
ENST00000642796.1
ENST00000642451.1
ENST00000331944.10
ENST00000414584.1
ENST00000646865.1
ENST00000643509.1
ENST00000643321.1
N
UC
500
400
300
200
100
0
ENST00000646386.1
ENST00000644478.1
ENST00000419680.6
ENST00000646636.1
ENST00000644040.1
ENST00000582552.1

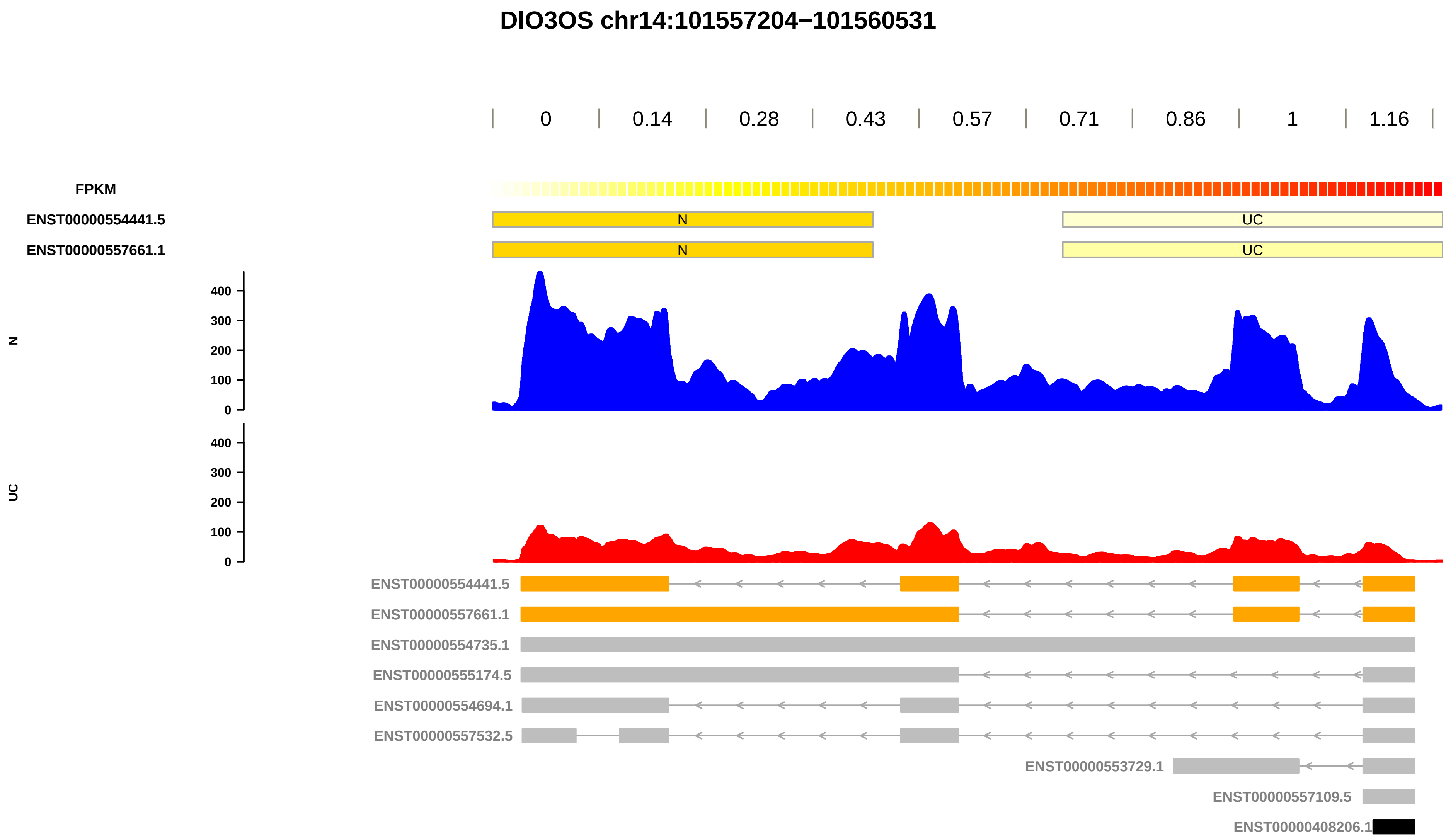
DIO3OS chr14:101557204−101560531
0
0.14
0.28
0.43
0.57
0.71
0.86
1
1.16
FPKM
ENST00000554441.5
ENST00000557661.1
N
UC
N
UC
N
400
300
200
100
0
UC
400
300
200
100
0
ENST00000554441.5
ENST00000557661.1
ENST00000554735.1
ENST00000555174.5
ENST00000554694.1
ENST00000557532.5
ENST00000553729.1
ENST00000557109.5
ENST00000408206.1

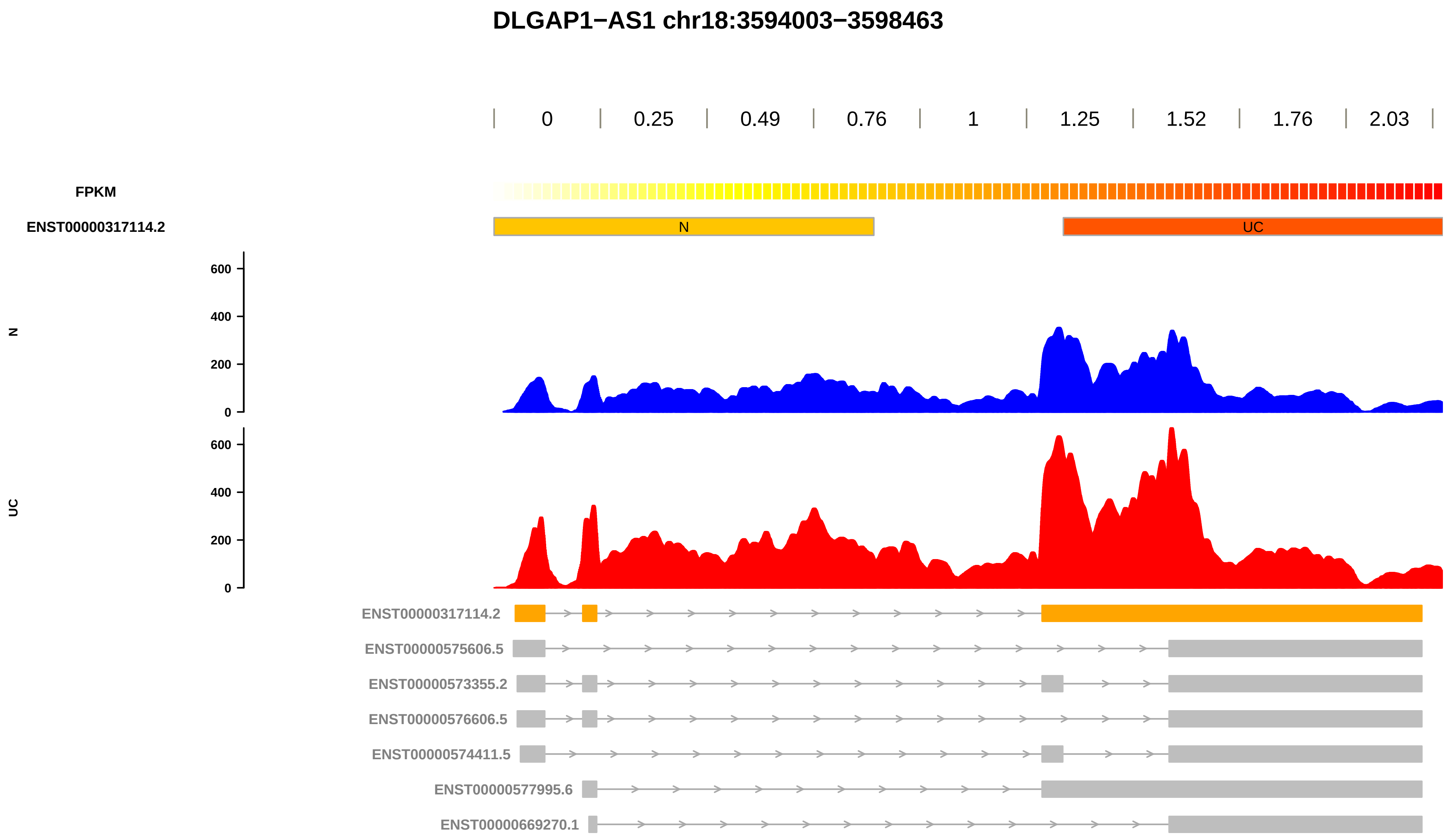
DLGAP1−AS1 chr18:3594003−3598463
0
0.25
0.49
0.76
1
1.25
1.52
1.76
2.03
FPKM
ENST00000317114.2
N
UC
600
400
200
0
N
UC
ENST00000317114.2
ENST00000575606.5
ENST00000573355.2
ENST00000576606.5
ENST00000574411.5
ENST00000577995.6
ENST00000669270.1

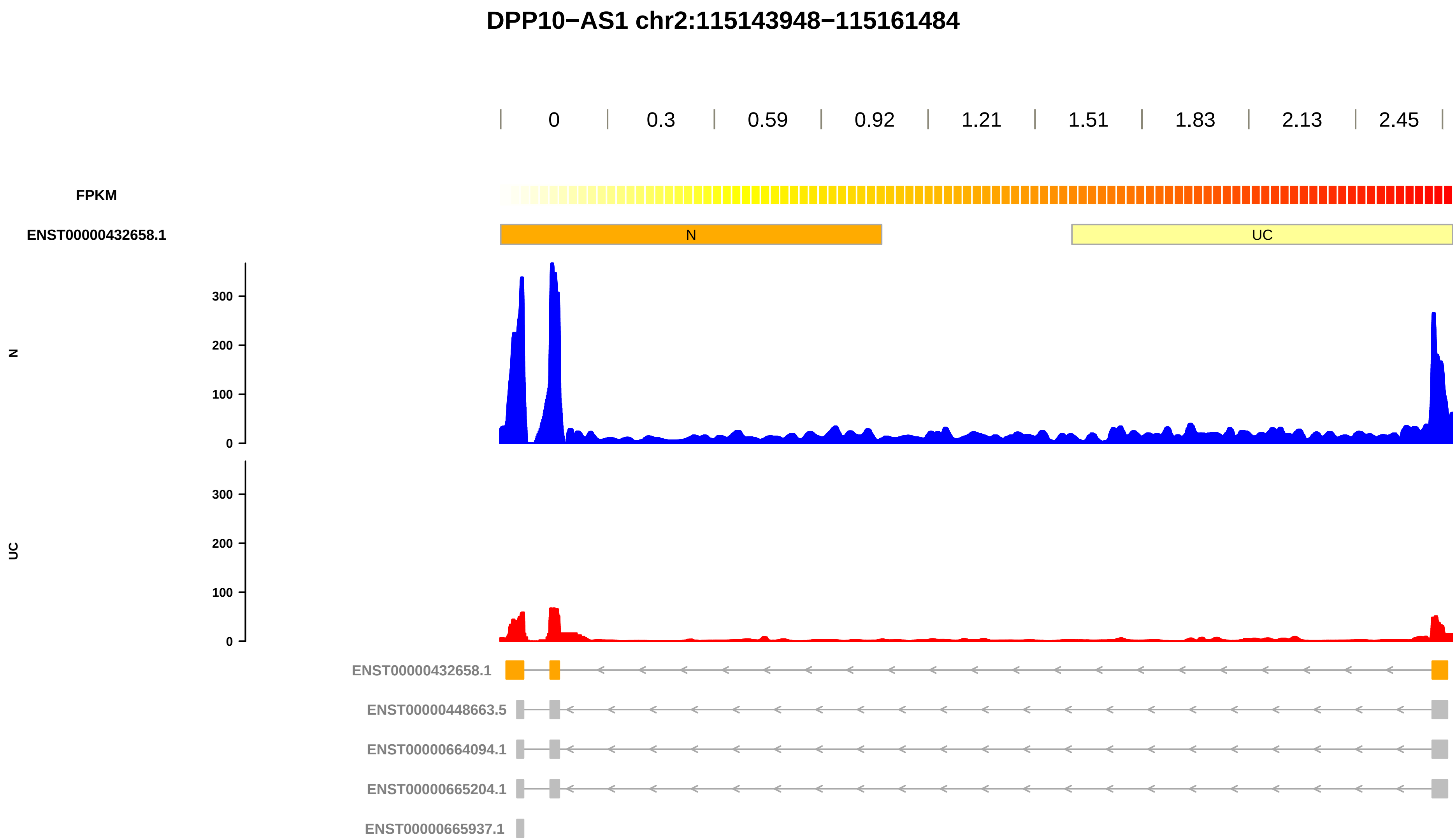
DPP10−AS1 chr2:115143948−115161484
0
0.3
0.59
0.92
1.21
1.51
1.83
2.13
2.45
FPKM
ENST00000432658.1
N
UC
300
200
100
0
N
300
200
100
0
UC
ENST00000432658.1
ENST00000448663.5
ENST00000664094.1
ENST00000665204.1
ENST00000665937.1

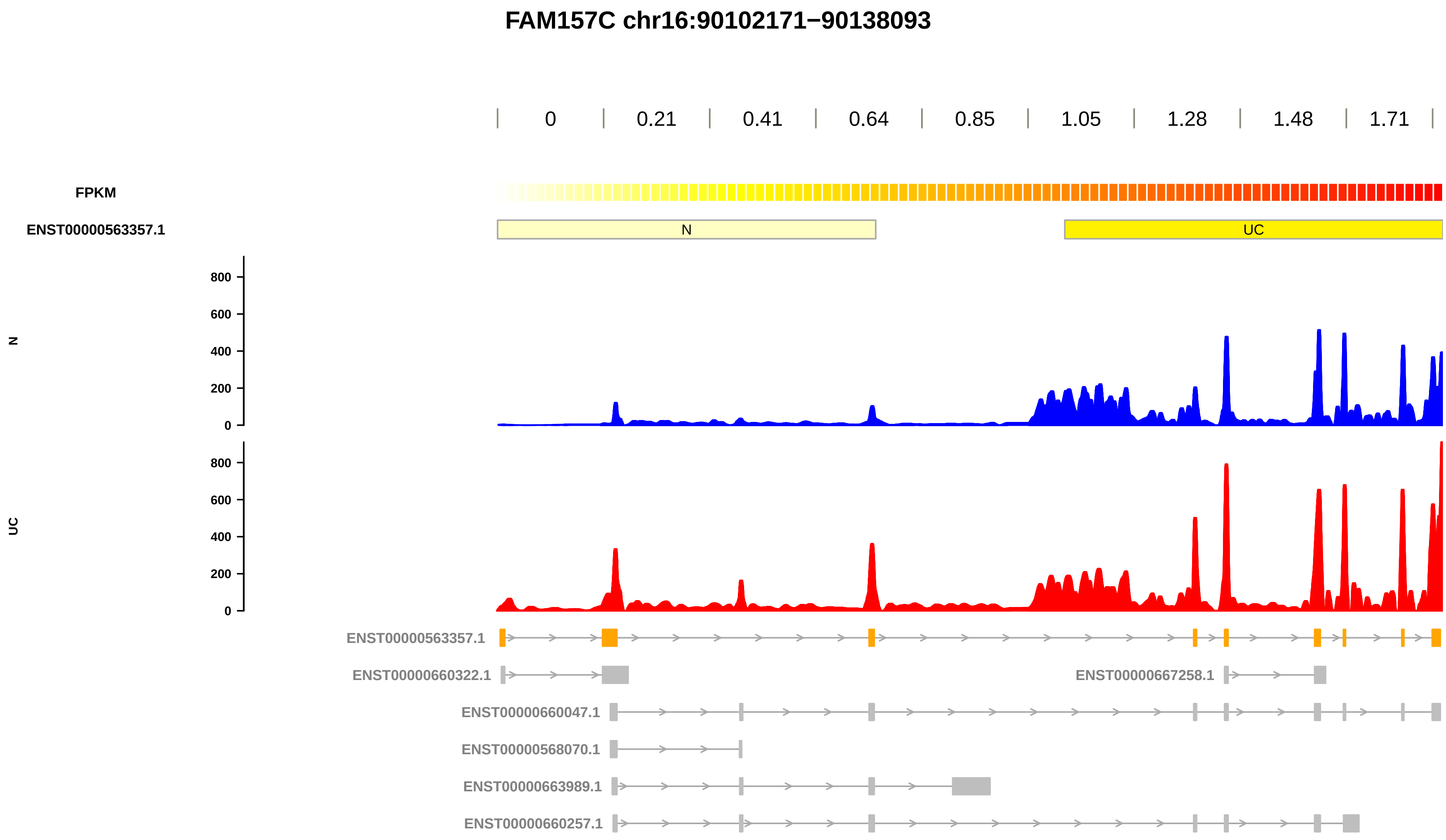

FAM157C chr16:90102171−90138093
0
0.21
0.41
0.64
0.85
1.05
1.28
1.48
1.71
FPKM
ENST00000563357.1
N
UC
800
600
400
200
0
N
800
600
400
200
0
UC
ENST00000563357.1
ENST00000660322.1
ENST00000667258.1
ENST00000660047.1
ENST00000568070.1
ENST00000663989.1
ENST00000660257.1

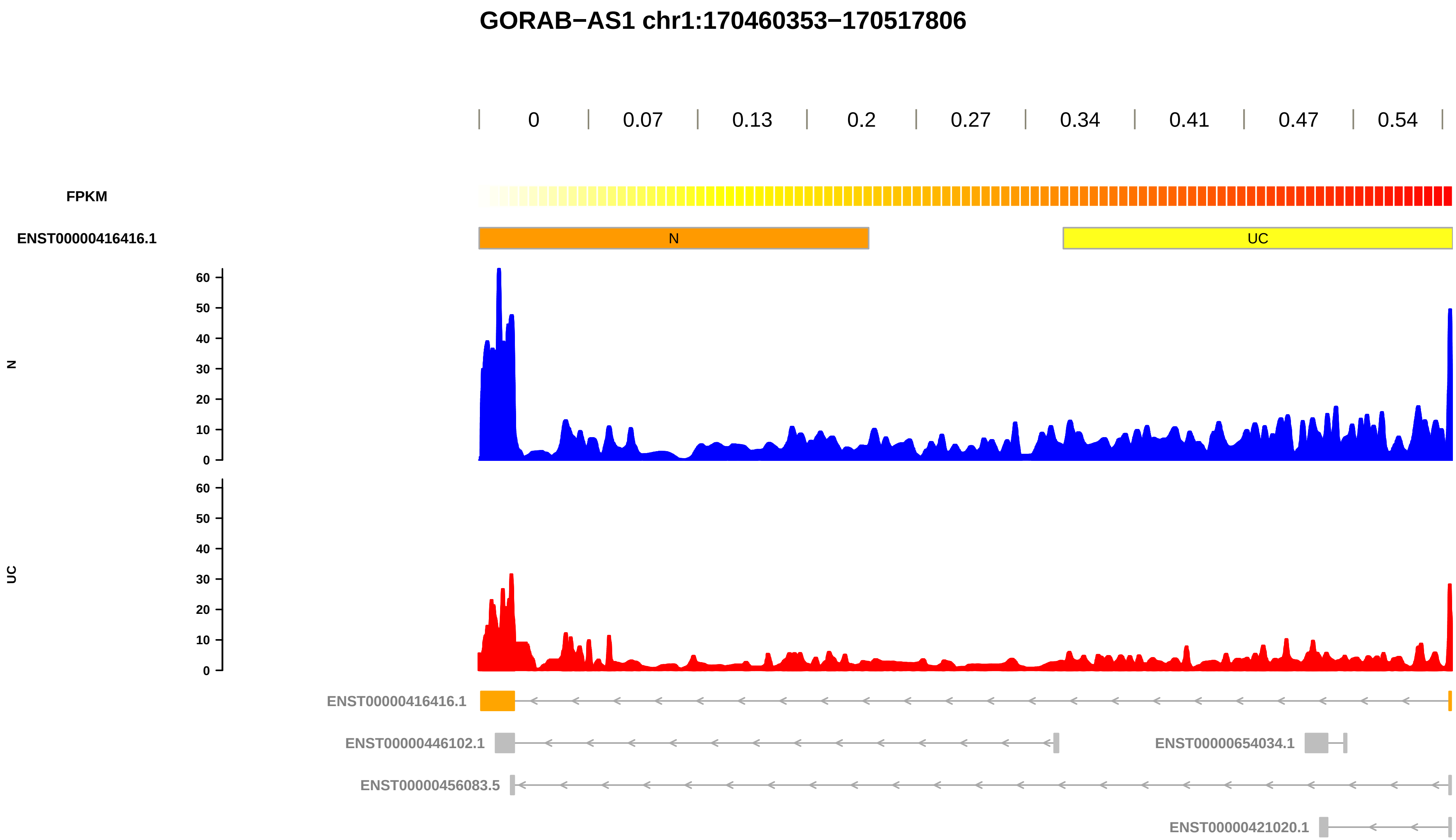
GORAB−AS1 chr1:170460353−170517806
0
0.07
0.13
0.2
0.27
0.34
0.41
0.47
0.54
FPKM
ENST00000416416.1
N
UC
60
50
40
30
20
10
0
N
UC
ENST00000416416.1
ENST00000446102.1
ENST00000654034.1
ENST00000456083.5
ENST00000421020.1

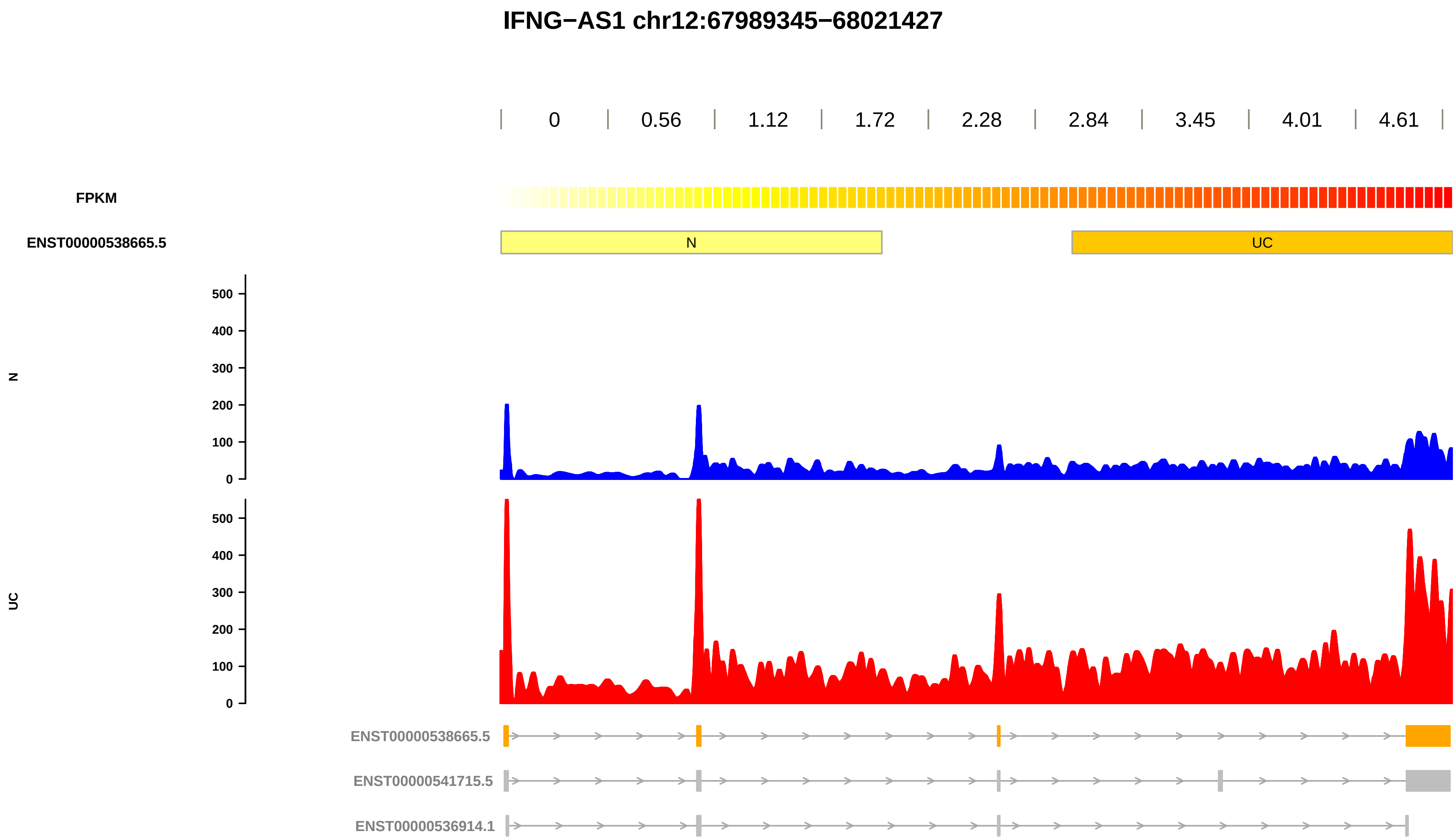
IFNG−AS1 chr12:67989345−68021427
0
0.56
1.12
1.72
2.28
2.84
3.45
4.01
4.61
FPKM
ENST00000538665.5
N
UC
N
UC
0
100
200
300
400
500
ENST00000538665.5
ENST00000541715.5
ENST00000536914.1

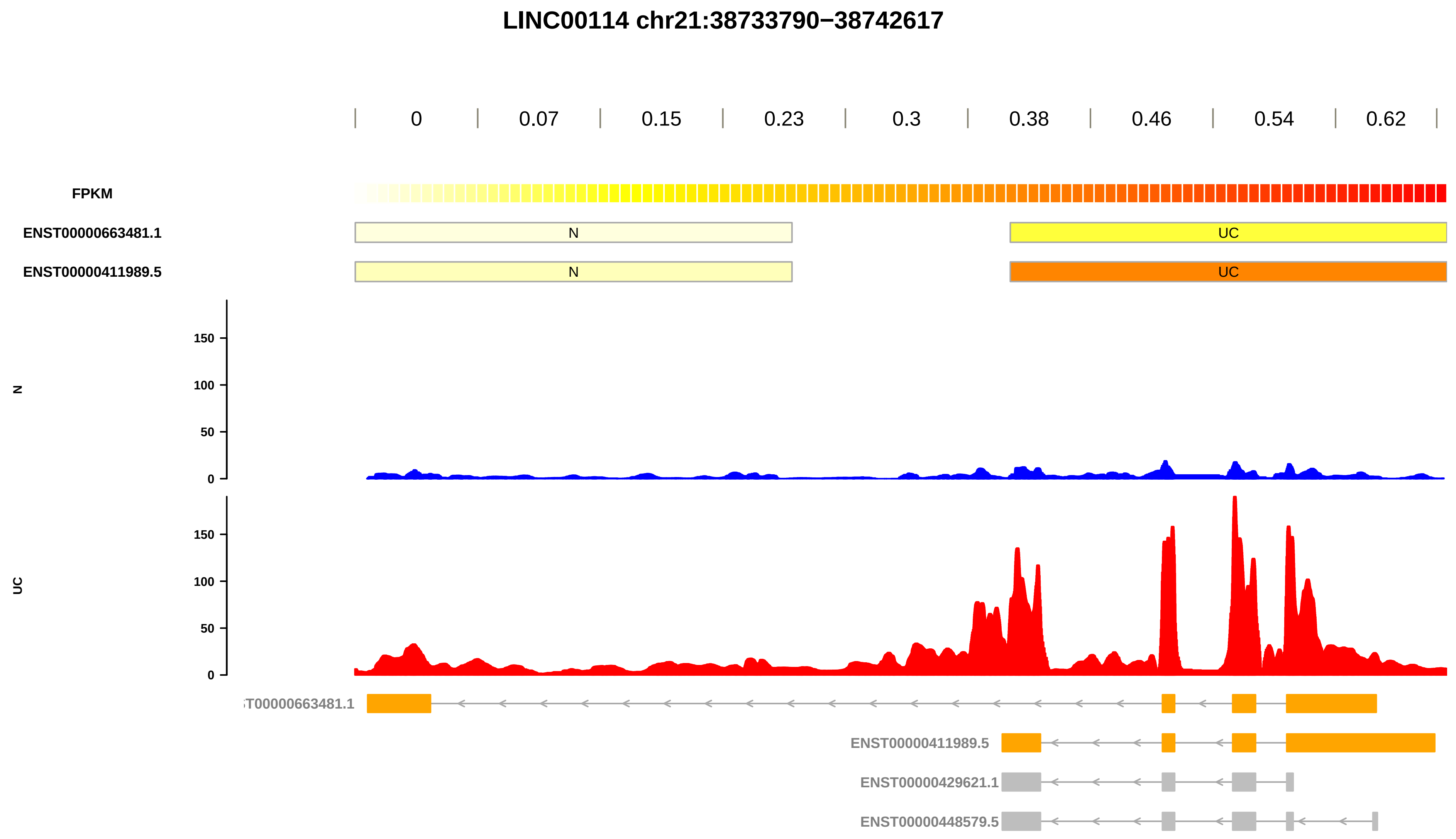

LINC00114 chr21:38733790−38742617
0
0.07
0.15
0.23
0.3
0.38
0.46
0.54
0.62
FPKM
ENST00000663481.1
N
UC
ENST00000411989.5
N
UC
N
UC
150
100
50
0
ENST00000411989.5
ENST00000429621.1
ENST00000448579.5

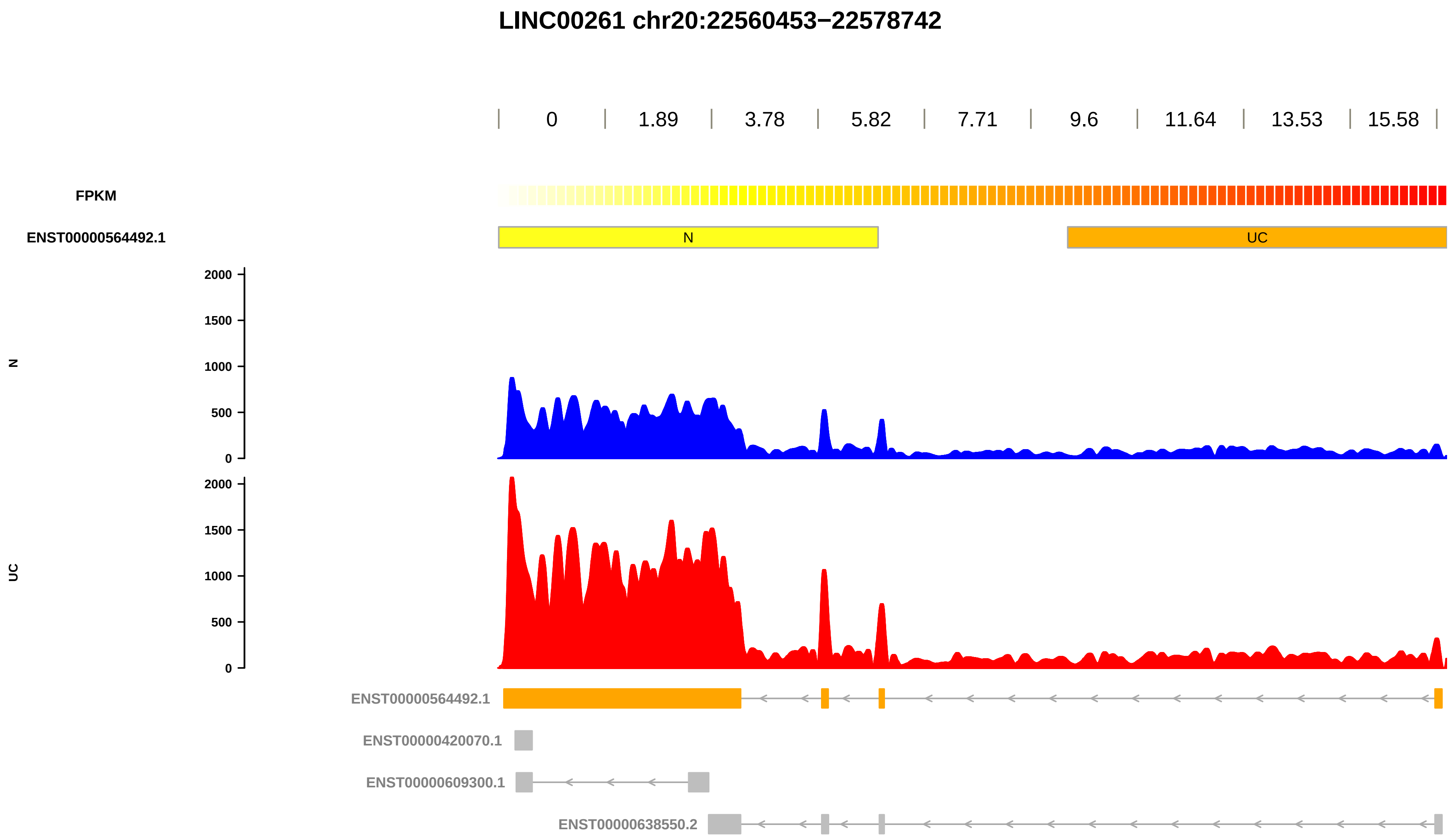
LINC00261 chr20:22560453−22578742
0
1.89
3.78
5.82
7.71
9.6
11.64
13.53
15.58
FPKM
ENST00000564492.1
N
UC
2000
1500
1000
500
0
N
UC
ENST00000564492.1
ENST00000420070.1
ENST00000609300.1
ENST00000638550.2

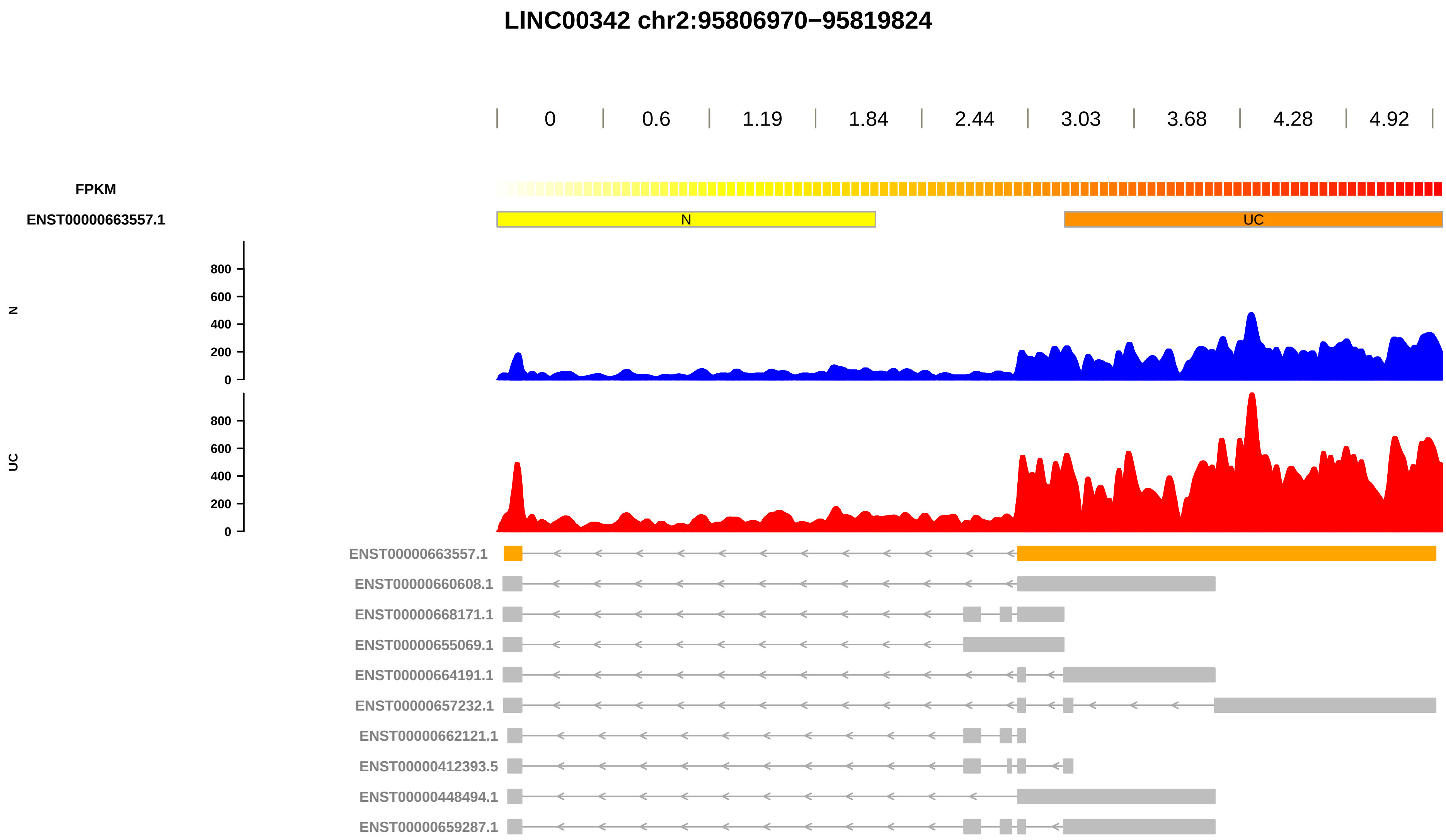
LINC00342 chr2:95806970−95819824
0
0.6
1.19
1.84
2.44
3.03
3.68
4.28
4.92
FPKM
ENST00000663557.1
N
UC
N
UC
800
600
400
200
0
ENST00000663557.1
ENST00000660608.1
ENST00000668171.1
ENST00000655069.1
ENST00000664191.1
ENST00000657232.1
ENST00000662121.1
ENST00000412393.5
ENST00000448494.1
ENST00000659287.1

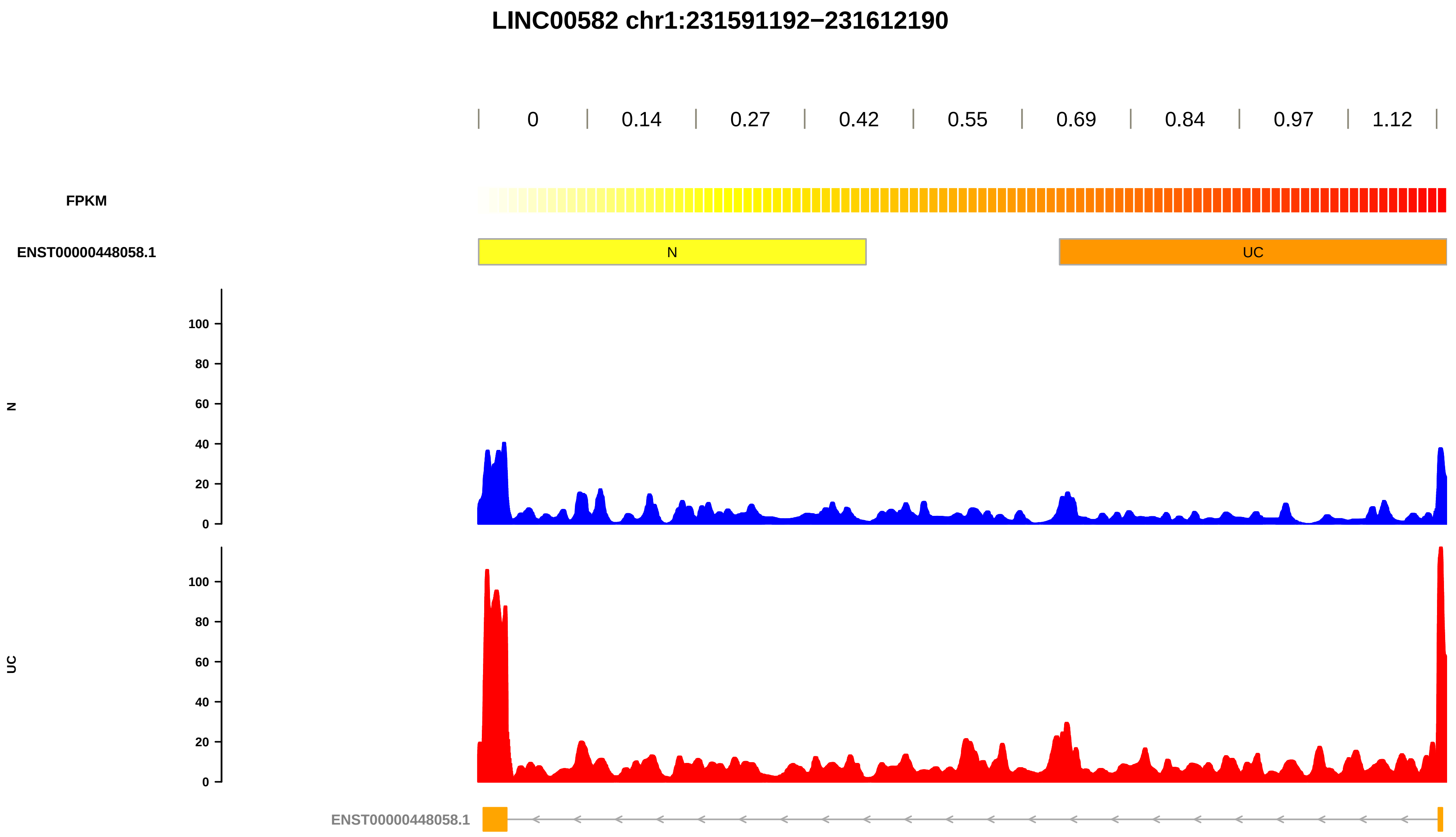
LINC00582 chr1:231591192−231612190
0
0.14
0.27
0.42
0.55
0.69
0.84
0.97
1.12
FPKM
ENST00000448058.1
N
UC
N
UC
0
20
40
60
80
100
ENST00000448058.1

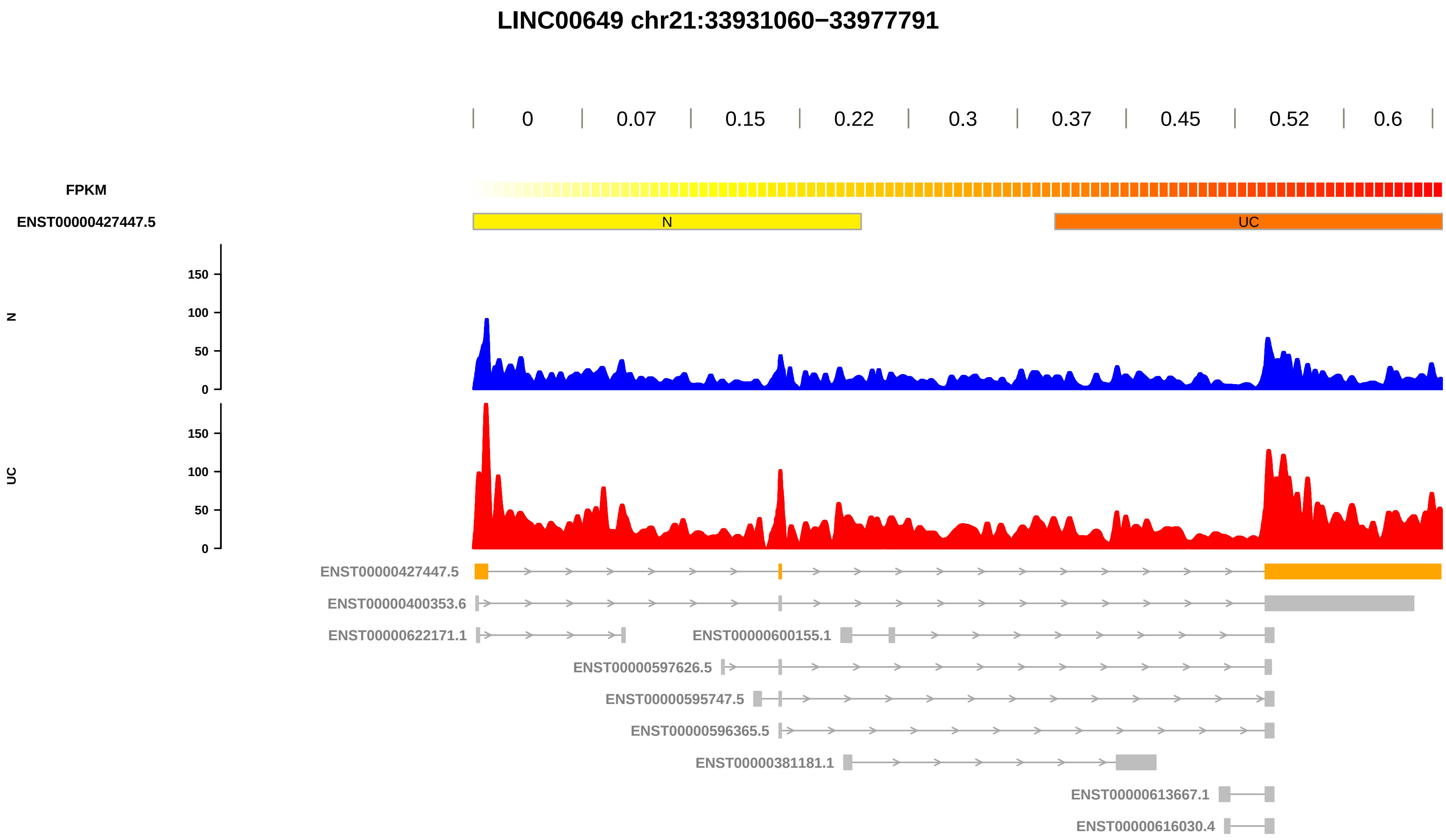
LINC00649 chr21:33931060−33977791
0
0.07
0.15
0.22
0.3
0.37
0.45
0.52
0.6
FPKM
ENST00000427447.5
N
UC
150
100
50
0
N
150
100
50
0
UC
ENST00000427447.5
ENST00000400353.6
ENST00000622171.1
ENST00000600155.1
ENST00000597626.5
ENST00000595747.5
ENST00000596365.5
ENST00000381181.1
ENST00000613667.1
ENST00000616030.4

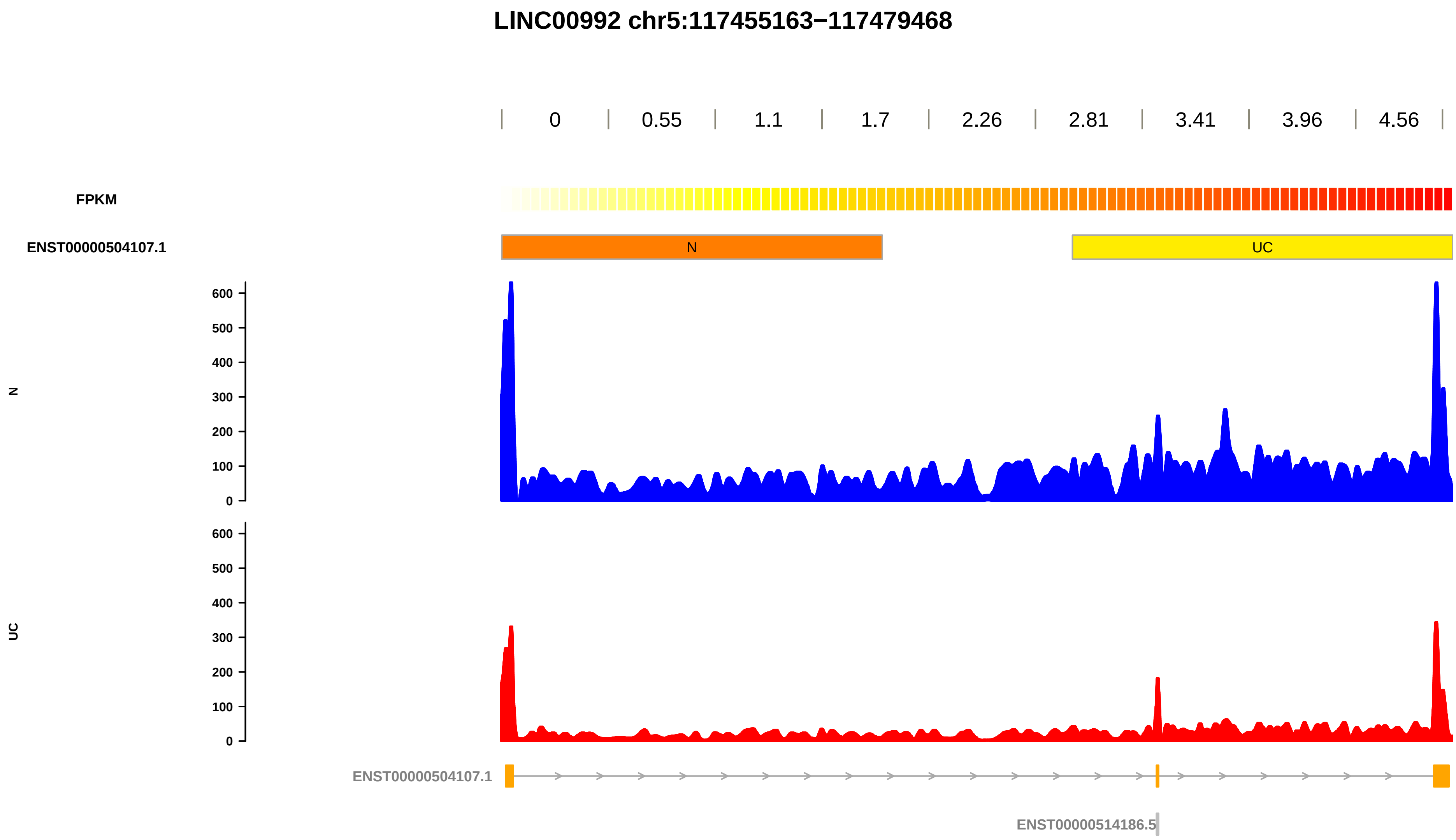
LINC00992 chr5:117455163−117479468
0
0.55
1.1
1.7
2.26
2.81
3.41
3.96
4.56
FPKM
ENST00000504107.1
N
UC
600
500
400
300
200
100
0
N
UC
ENST00000504107.1
ENST00000514186.5

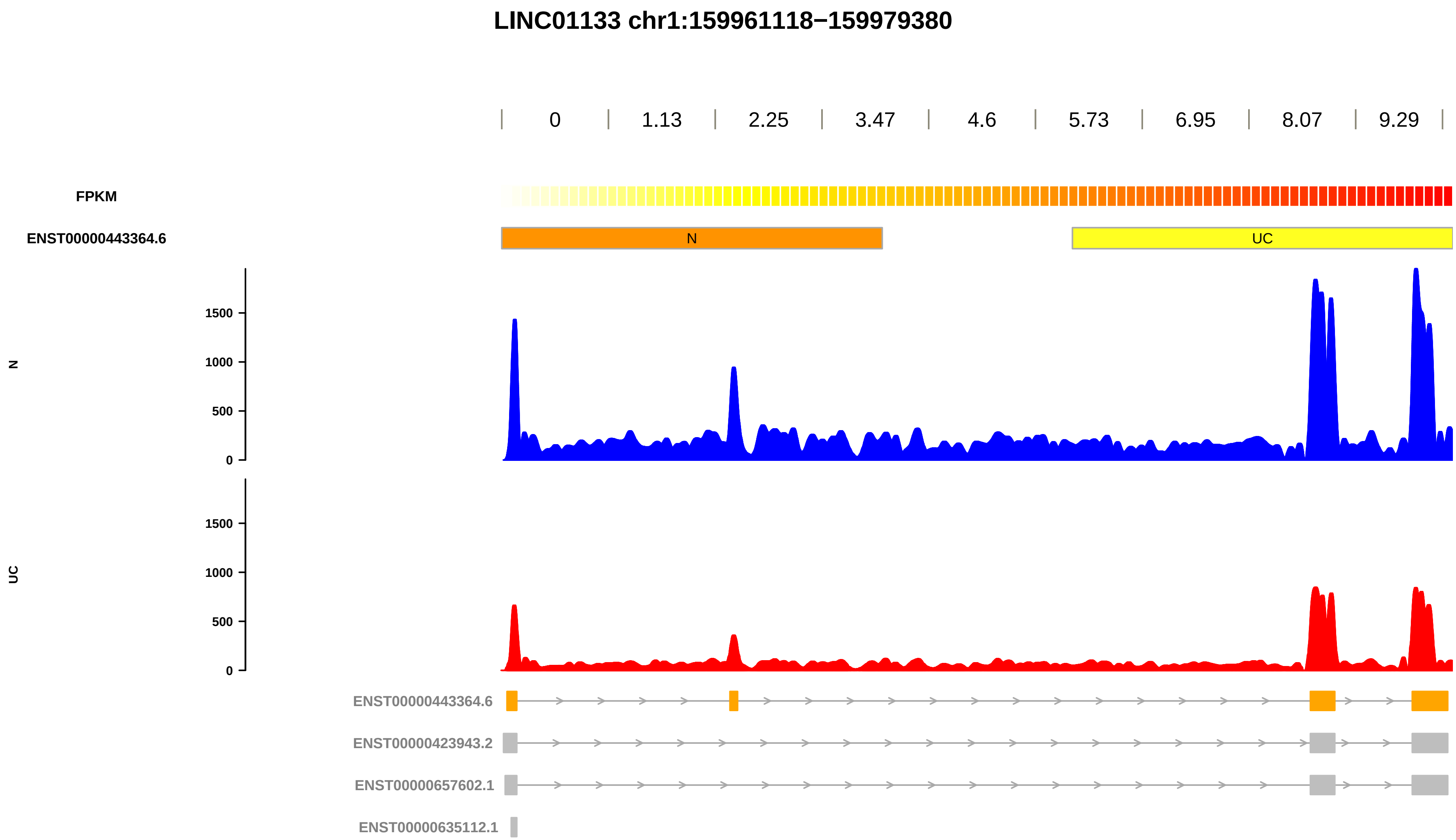

LINC01133 chr1:159961118−159979380
0
1.13
2.25
3.47
4.6
5.73
6.95
8.07
9.29
FPKM
ENST00000443364.6
N
UC
N
1500
1000
500
0
UC
1500
1000
500
0
ENST00000443364.6
ENST00000423943.2
ENST00000657602.1
ENST00000635112.1

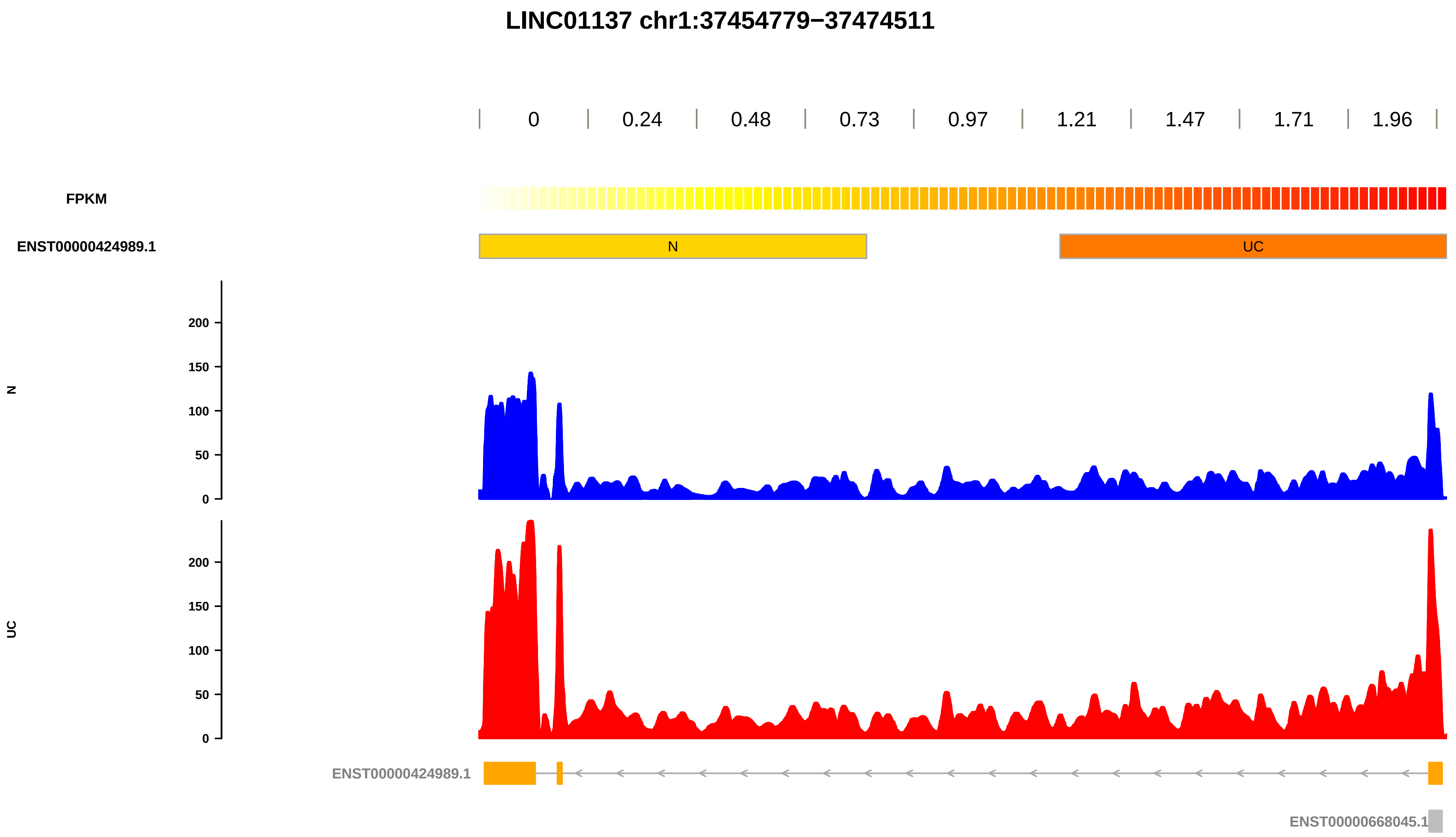

LINC01137 chr1:37454779−37474511
0
0.24
0.48
0.73
0.97
1.21
1.47
1.71
1.96
FPKM
ENST00000424989.1
N
UC
N
0
50
100
150
200
UC
0
50
100
150
200
ENST00000424989.1
ENST00000668045.1

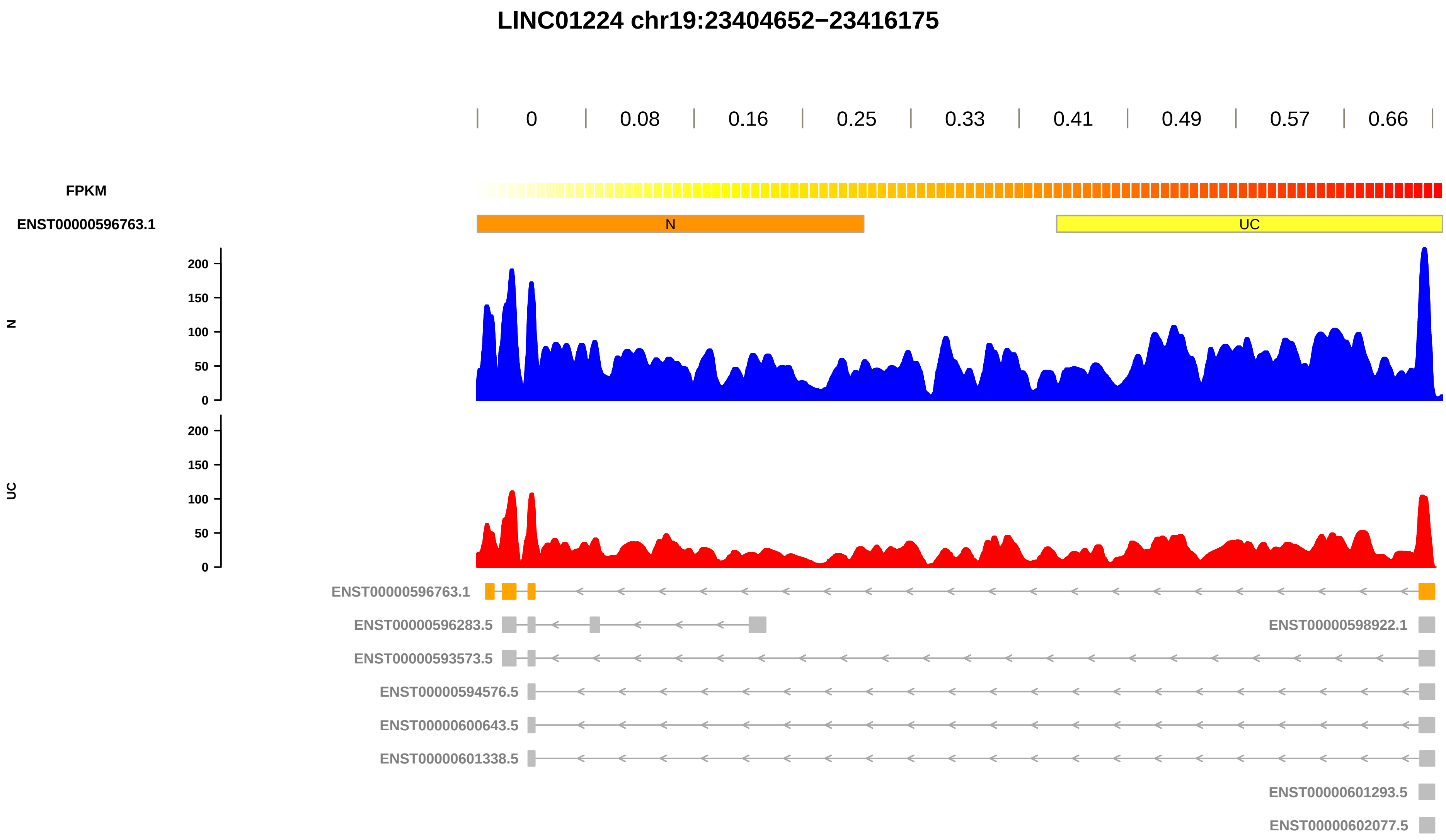

LINC01224 chr19:23404652−23416175
0
0.08
0.16
0.25
0.33
0.41
0.49
0.57
0.66
FPKM
ENST00000596763.1
N
UC
200
150
100
50
0
N
UC
ENST00000596763.1
ENST00000596283.5
ENST00000598922.1
ENST00000593573.5
ENST00000594576.5
ENST00000600643.5
ENST00000601338.5
ENST00000601293.5
ENST00000602077.5

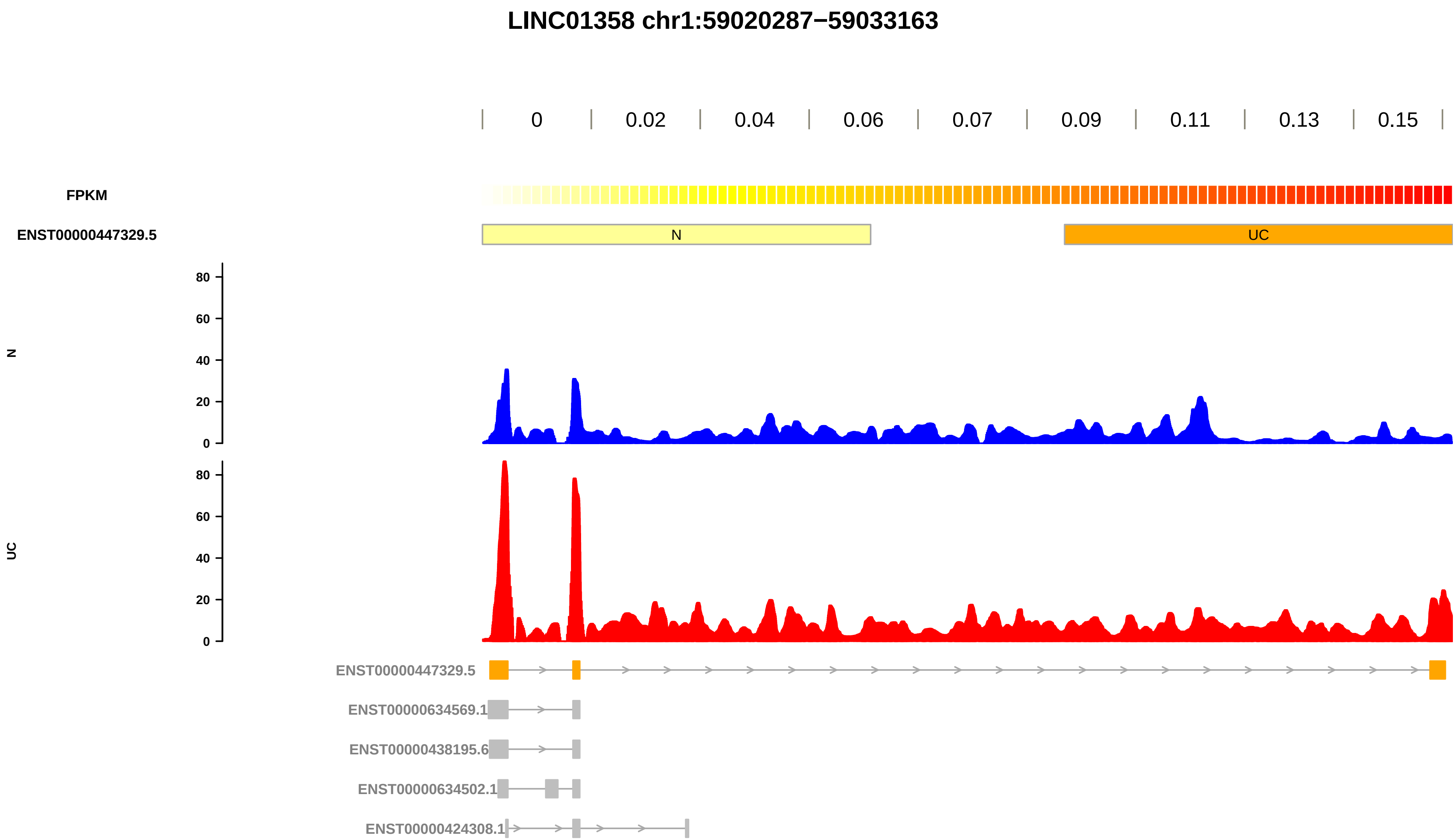
LINC01358 chr1:59020287−59033163
0
0.02
0.04
0.06
0.07
0.09
0.11
0.13
0.15
FPKM
ENST00000447329.5
N
UC
N
UC
0
20
40
60
80
ENST00000447329.5
ENST00000634569.1
ENST00000438195.6
ENST00000634502.1
ENST00000424308.1

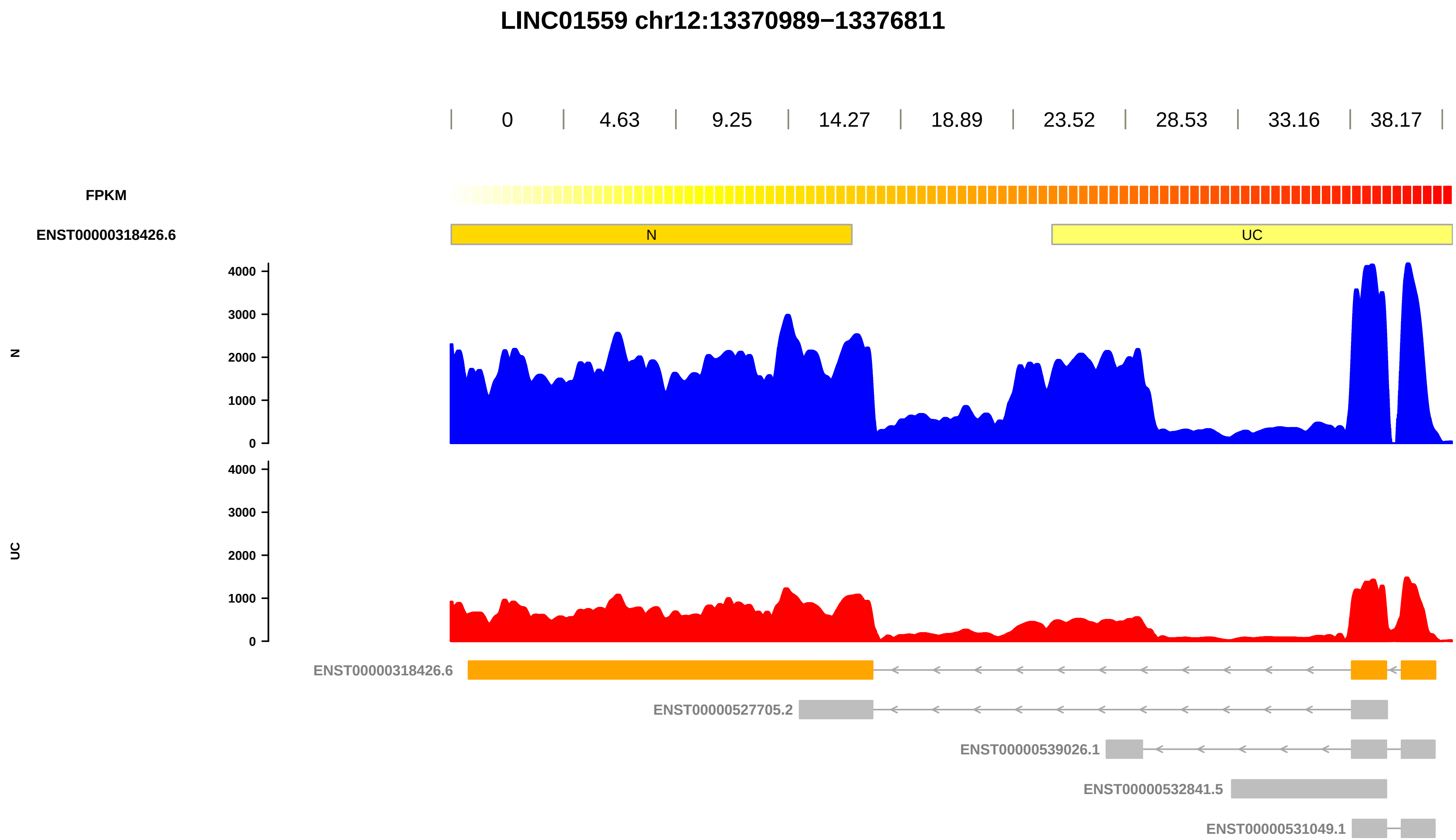
LINC01559 chr12:13370989−13376811
0
4.63
9.25
14.27
18.89
23.52
28.53
33.16
38.17
FPKM
ENST00000318426.6
N
UC
N
UC
4000
3000
2000
1000
0
ENST00000318426.6
ENST00000527705.2
ENST00000539026.1
ENST00000532841.5
ENST00000531049.1

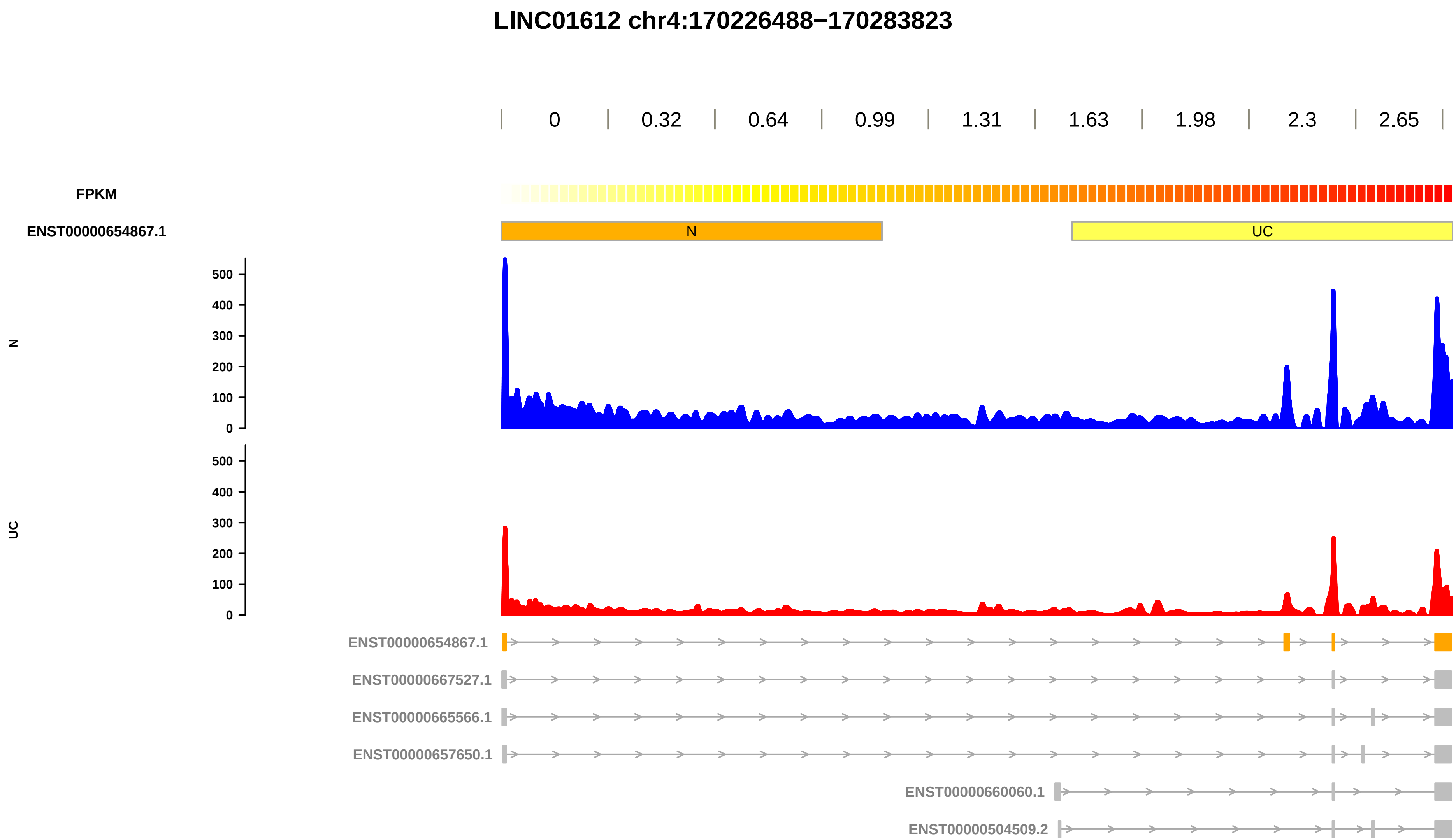
LINC01612 chr4:170226488−170283823
0
0.32
0.64
0.99
1.31
1.63
1.98
2.3
2.65
FPKM
ENST00000654867.1
N
UC
500
400
300
200
100
0
N
500
400
300
200
100
0
UC
ENST00000654867.1
ENST00000667527.1
ENST00000665566.1
ENST00000657650.1
ENST00000660060.1
ENST00000504509.2

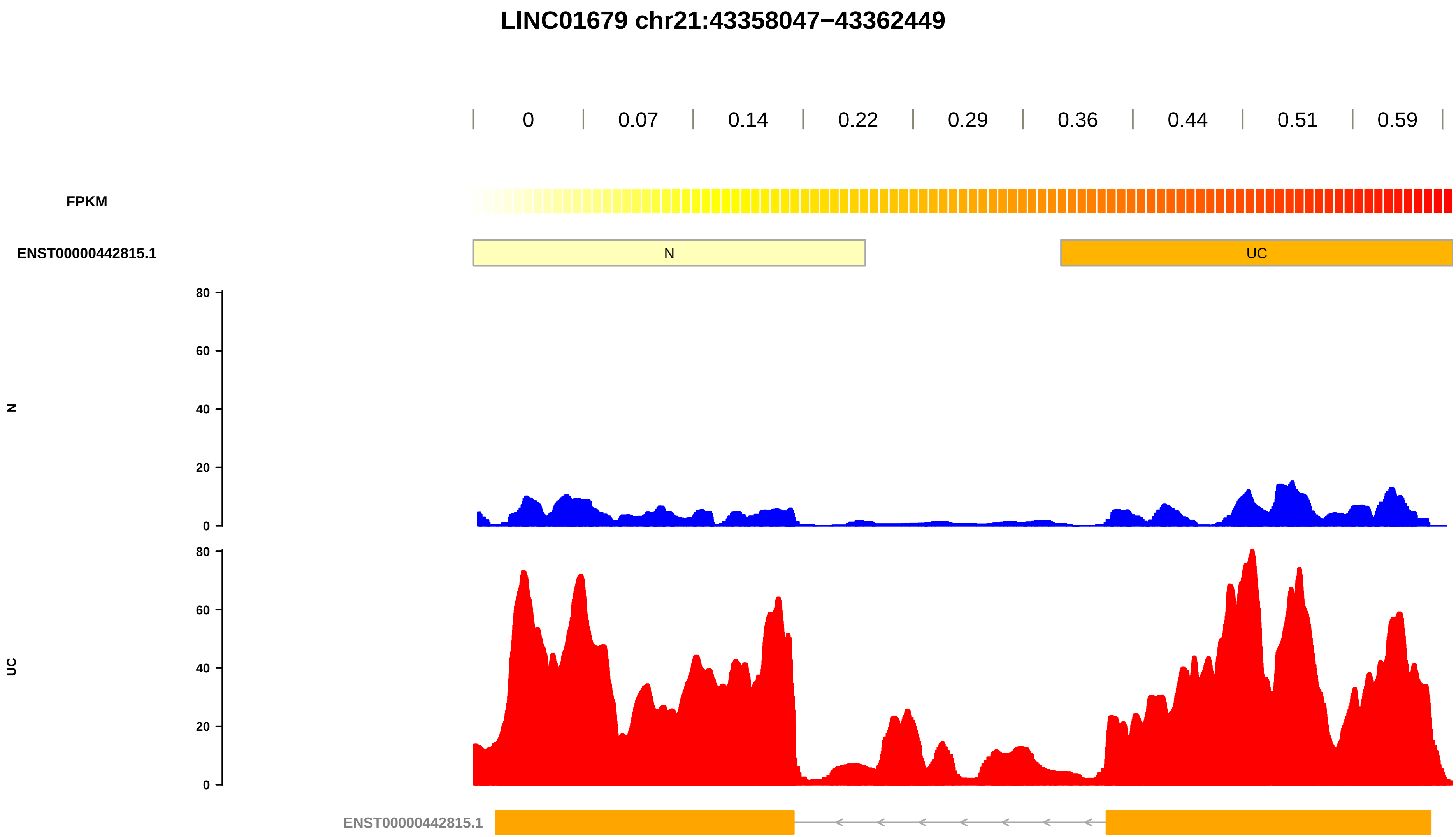
LINC01679 chr21:43358047−43362449
0
0.07
0.14
0.22
0.29
0.36
0.44
0.51
0.59
FPKM
ENST00000442815.1
N
UC
80
60
40
20
0
N
UC
ENST00000442815.1

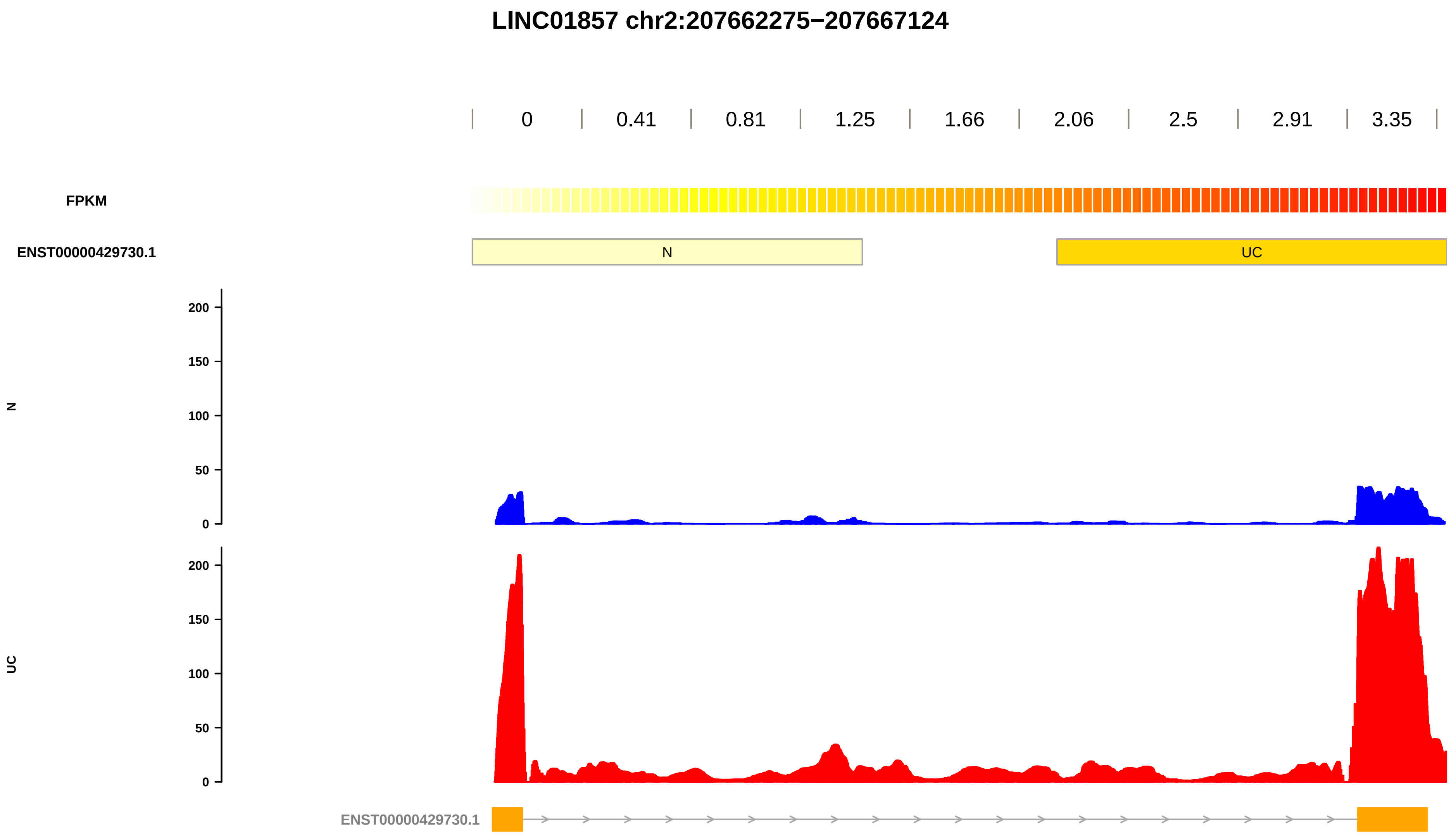
LINC01857 chr2:207662275−207667124
0
0.41
0.81
1.25
1.66
2.06
2.5
2.91
3.35
FPKM
ENST00000429730.1
N
UC
200
150
100
50
0
N
200
150
100
50
0
UC
ENST00000429730.1

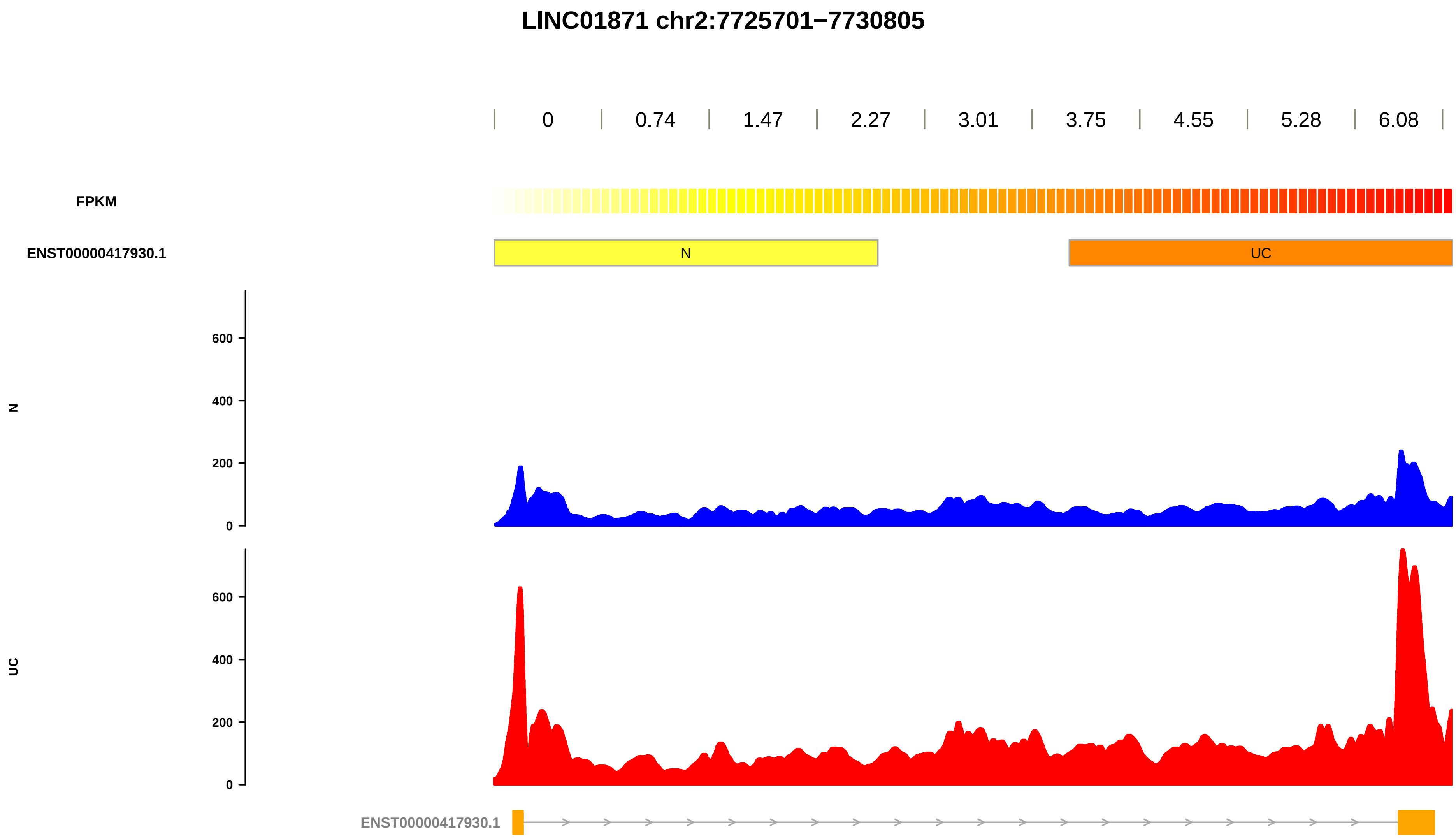

LINC01871 chr2:7725701−7730805
0
0.74
1.47
2.27
3.01
3.75
4.55
5.28
6.08
FPKM
ENST00000417930.1
N
UC
600
400
200
0
N
600
400
200
0
UC
ENST00000417930.1

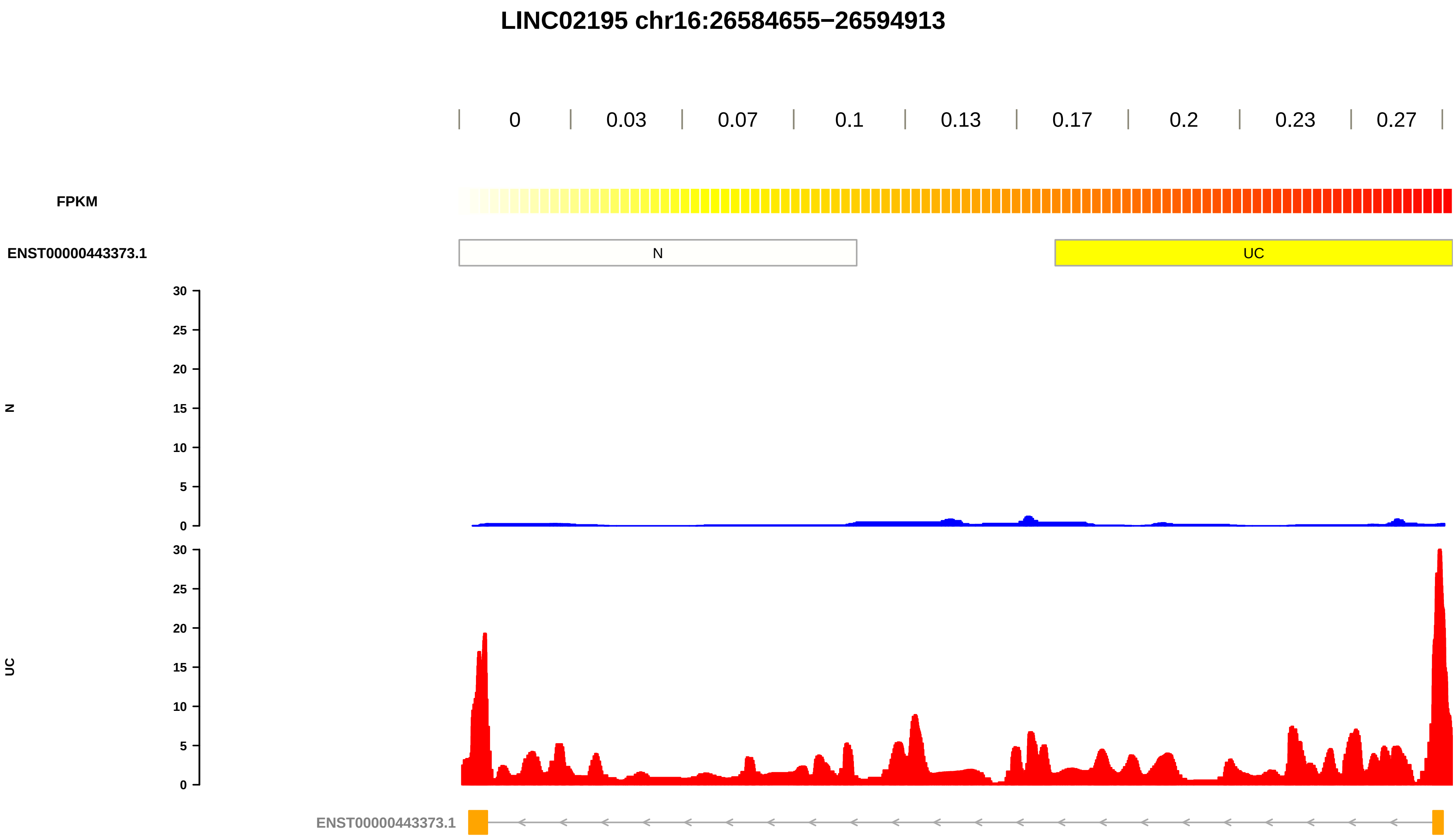

LINC02195 chr16:26584655−26594913
0
0.03
0.07
0.1
0.13
0.17
0.2
0.23
0.27
FPKM
ENST00000443373.1
N
UC
30
25
20
15
10
5
0
N
UC
ENST00000443373.1

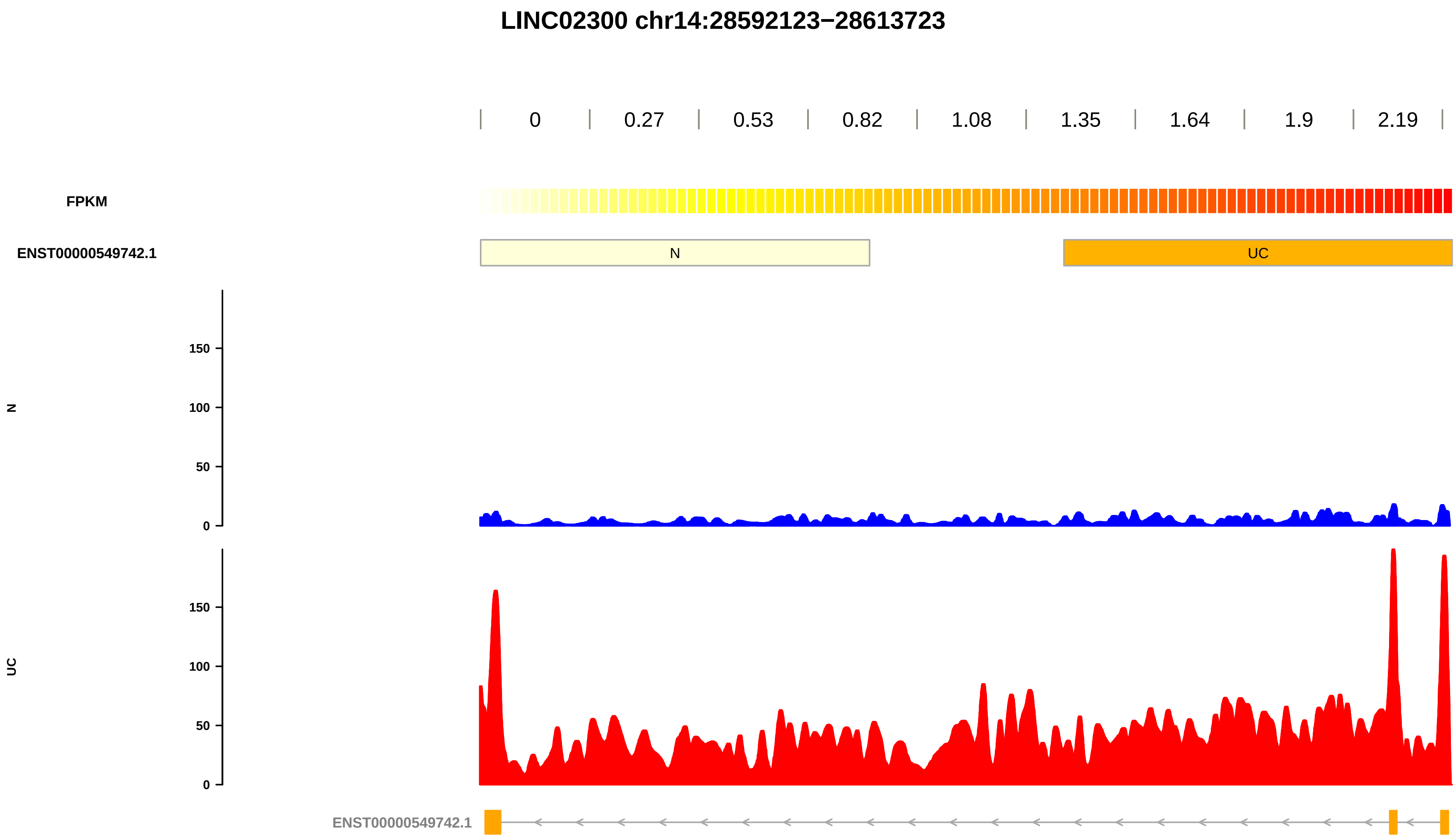

LINC02300 chr14:28592123−28613723
0
0.27
0.53
0.82
1.08
1.35
1.64
1.9
2.19
FPKM
ENST00000549742.1
N
UC
150
100
50
0
N
150
100
50
0
UC
ENST00000549742.1

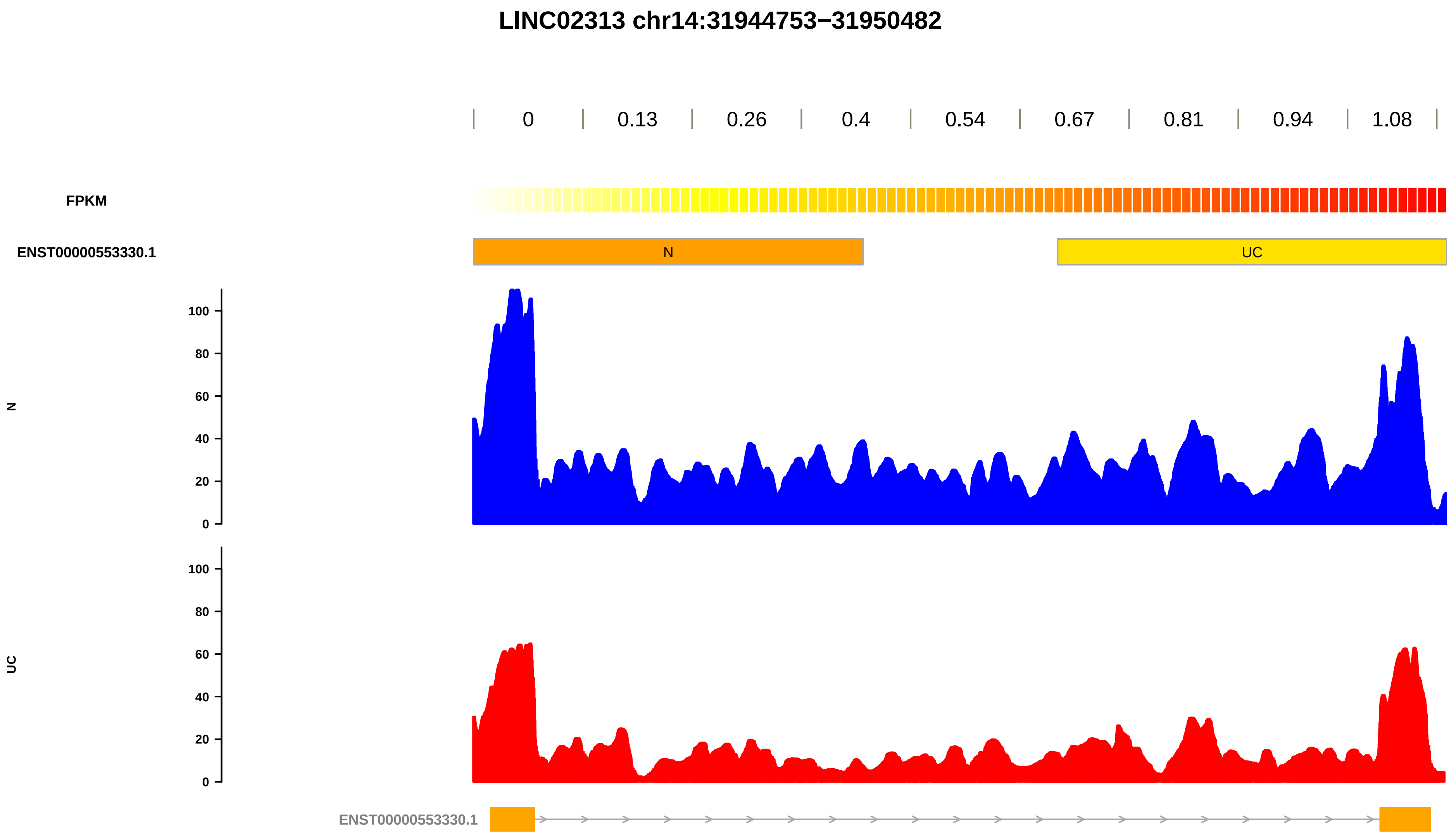

LINC02313 chr14:31944753−31950482
0
0.13
0.26
0.4
0.54
0.67
0.81
0.94
1.08
FPKM
ENST00000553330.1
N
UC
N
100
80
60
40
20
0
UC
100
80
60
40
20
0
ENST00000553330.1

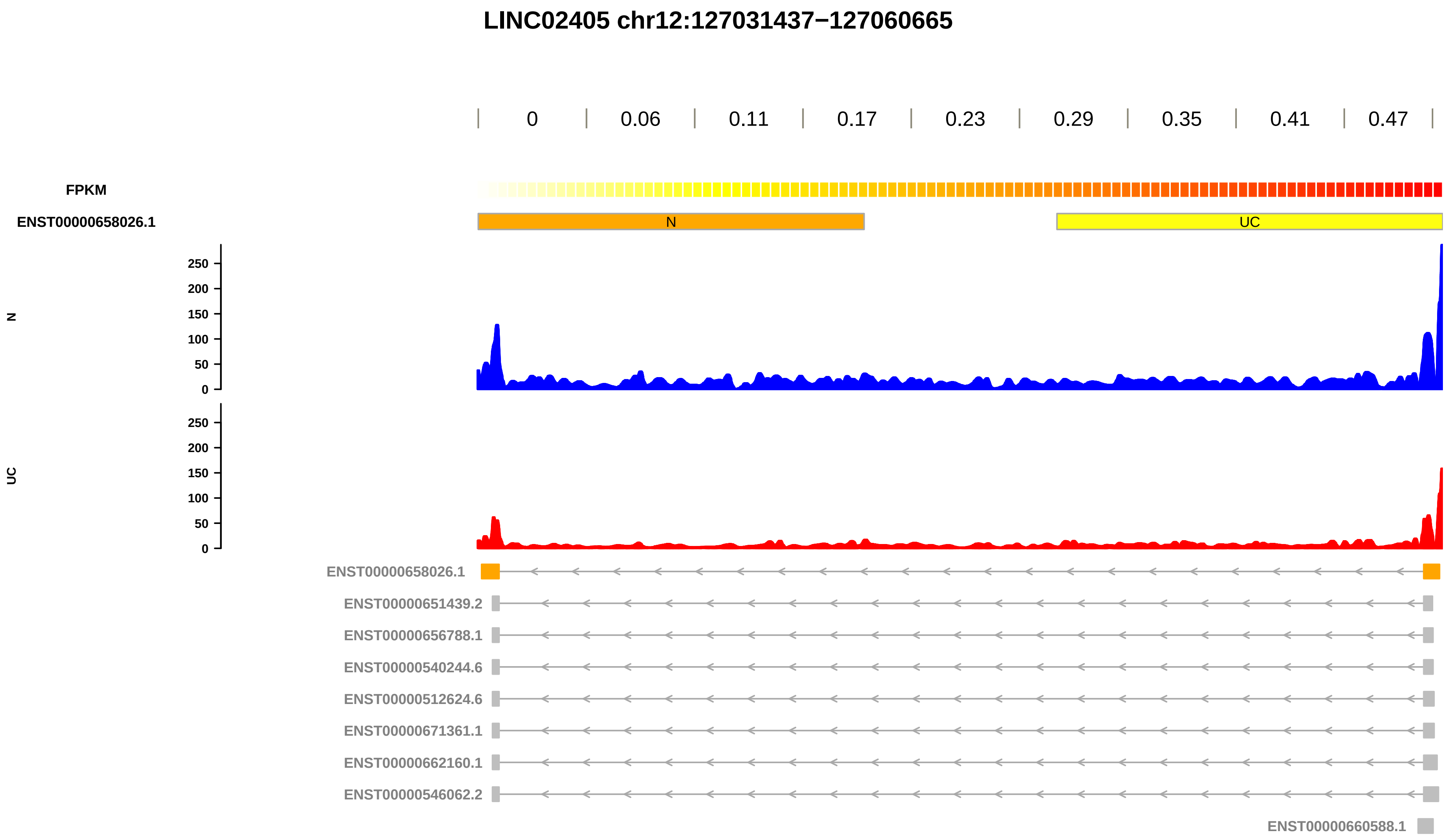

LINC02405 chr12:127031437−127060665
0
0.06
0.11
0.17
0.23
0.29
0.35
0.41
0.47
FPKM
ENST00000658026.1
N
UC
250
200
150
100
50
0
N
UC
ENST00000658026.1
ENST00000651439.2
ENST00000656788.1
ENST00000540244.6
ENST00000512624.6
ENST00000671361.1
ENST00000662160.1
ENST00000546062.2
ENST00000660588.1

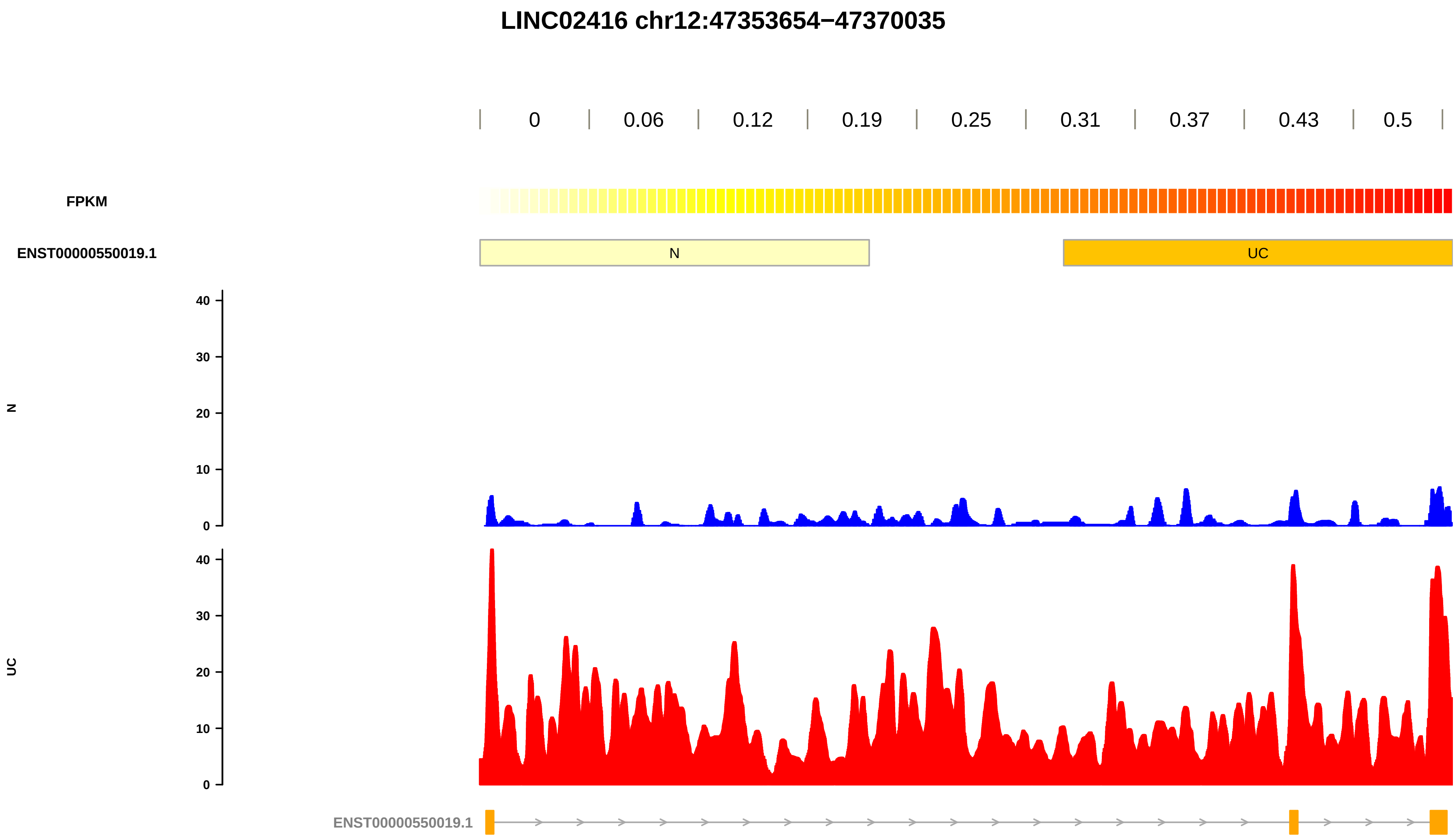

LINC02416 chr12:47353654−47370035
0
0.06
0.12
0.19
0.25
0.31
0.37
0.43
0.5
FPKM
ENST00000550019.1
N
UC
40
30
20
10
0
N
40
30
20
10
0
UC
ENST00000550019.1

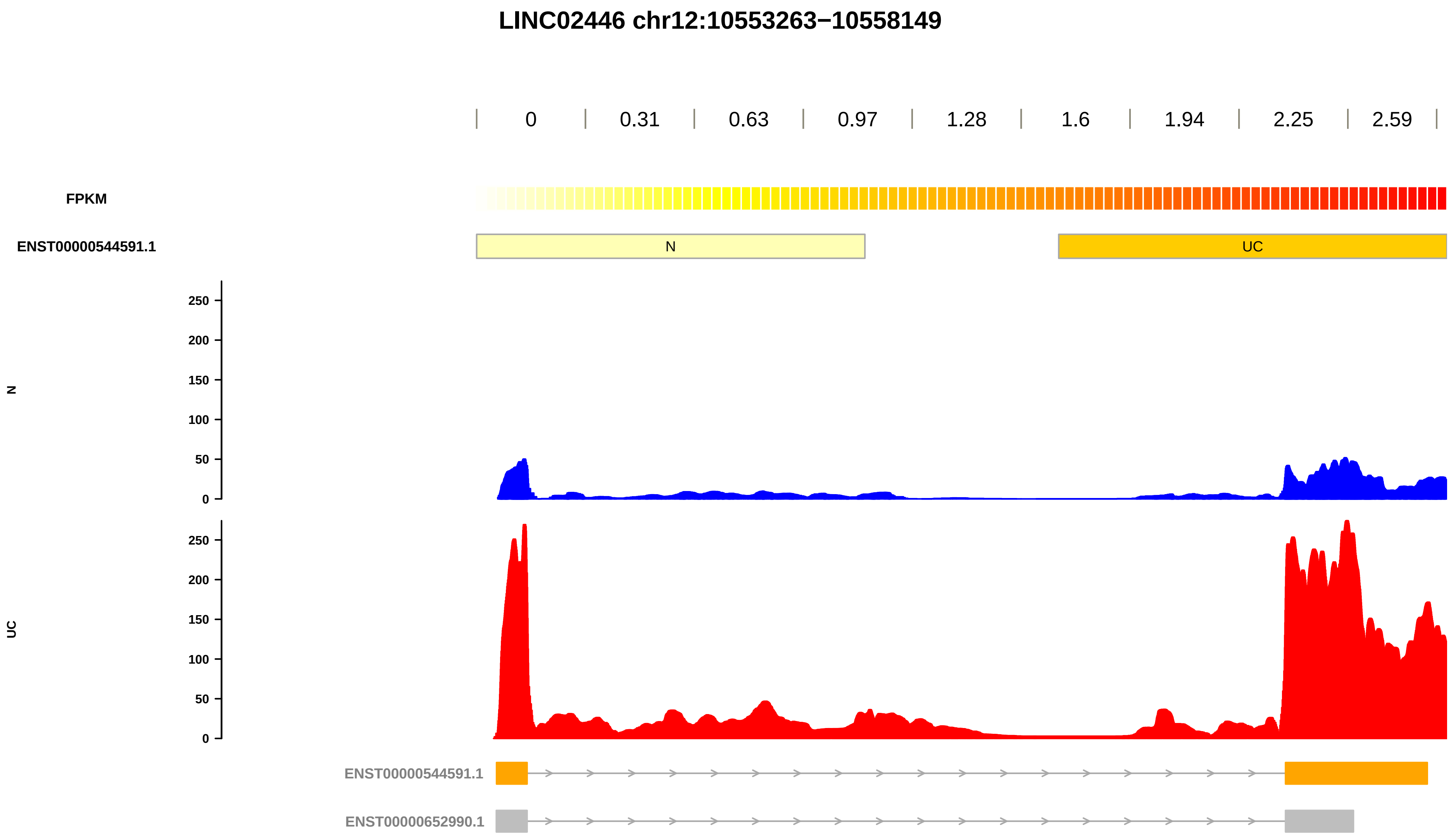

LINC02446 chr12:10553263−10558149
0
0.31
0.63
0.97
1.28
1.6
1.94
2.25
2.59
FPKM
ENST00000544591.1
N
UC
N
UC
250
200
150
100
50
0
ENST00000544591.1
ENST00000652990.1

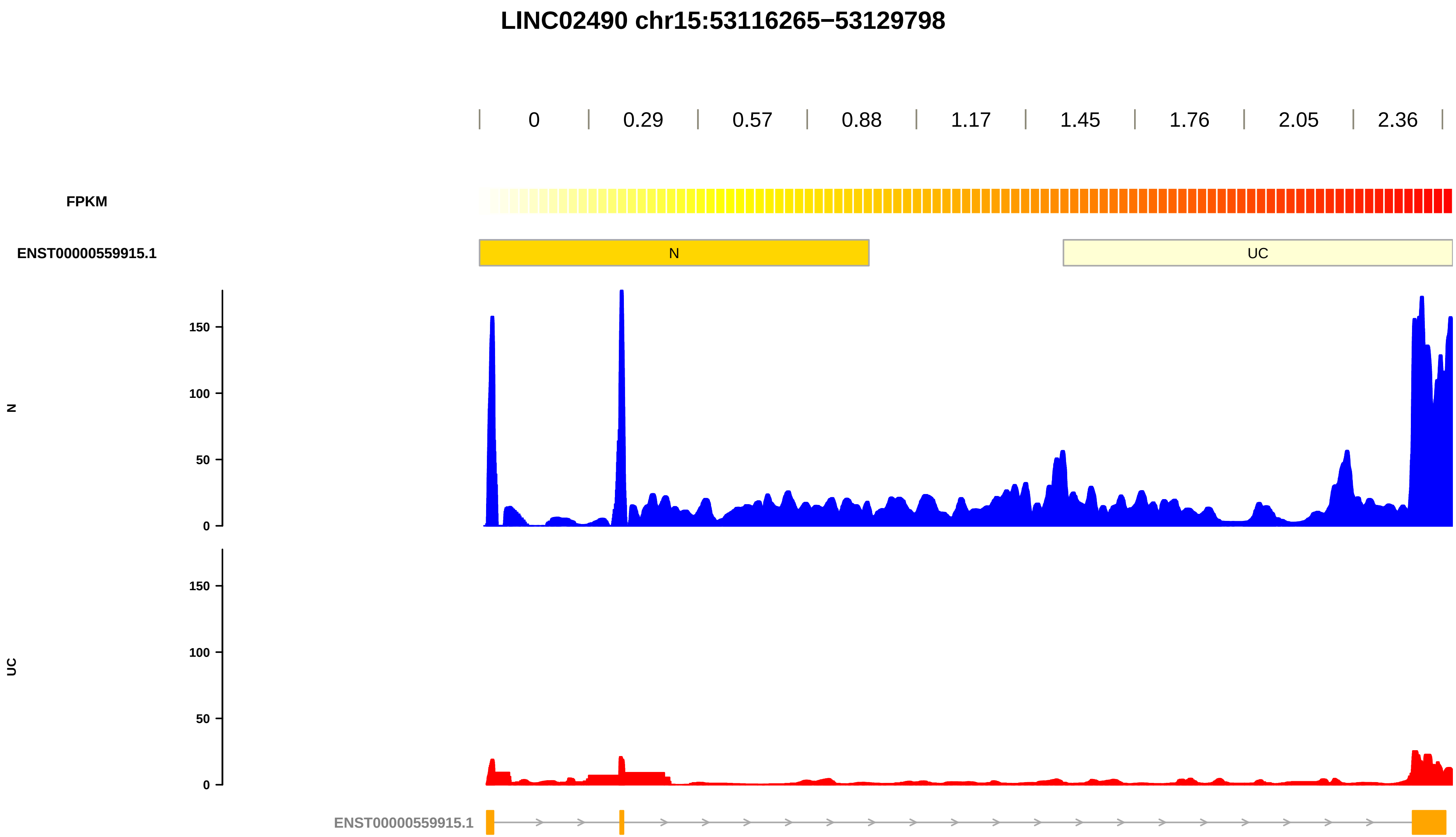
LINC02490 chr15:53116265−53129798
0
0.29
0.57
0.88
1.17
1.45
1.76
2.05
2.36
FPKM
ENST00000559915.1
N
UC
N
150
100
50
0
UC
150
100
50
0
ENST00000559915.1

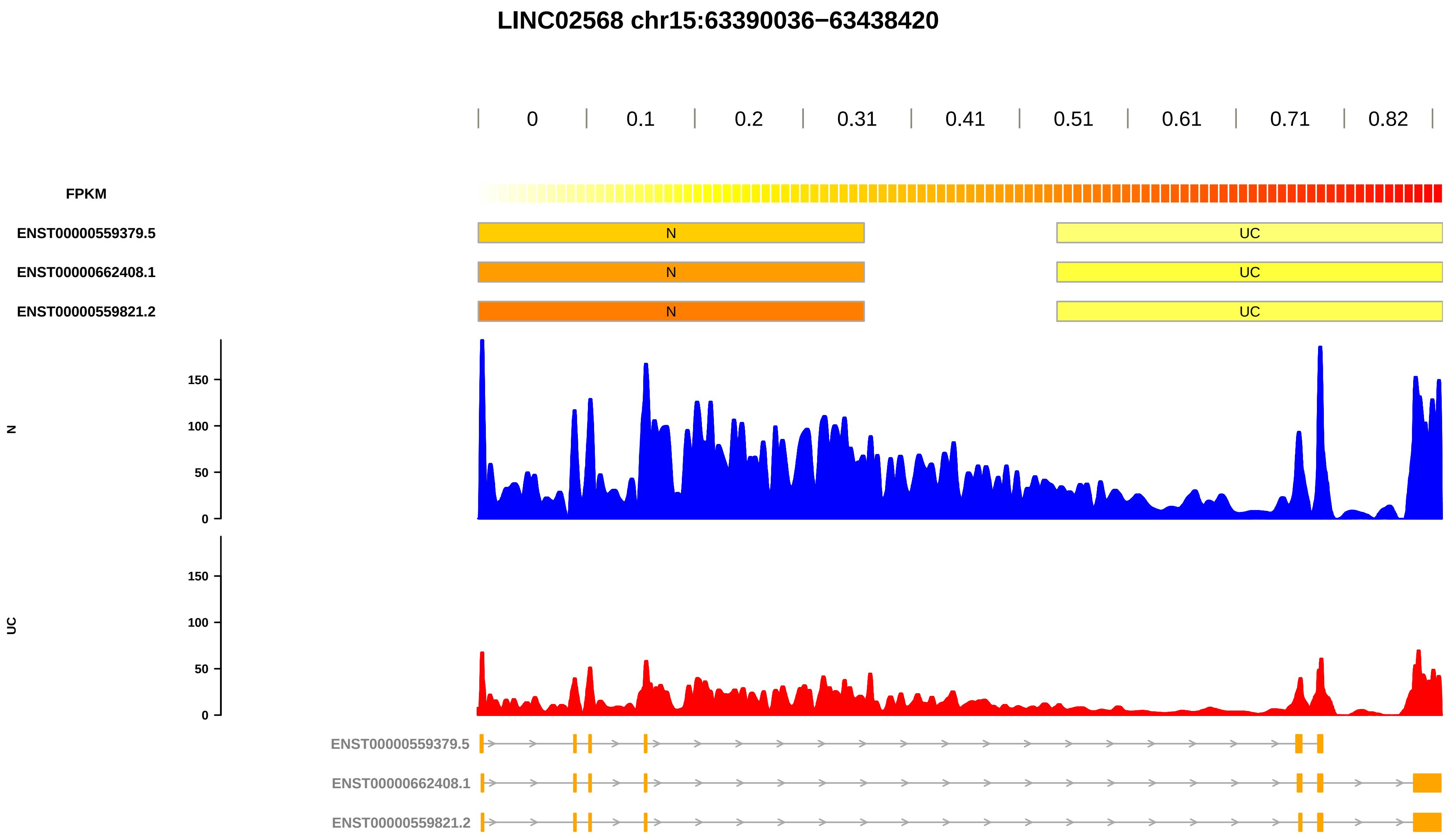
LINC02568 chr15:63390036−63438420
0
0.1
0.2
0.31
0.41
0.51
0.61
0.71
0.82
FPKM
ENST00000559379.5
ENST00000662408.1
ENST00000559821.2
N
UC
N
UC
N
UC
150
100
50
0
N
150
100
50
0
UC
ENST00000559379.5
ENST00000662408.1
ENST00000559821.2

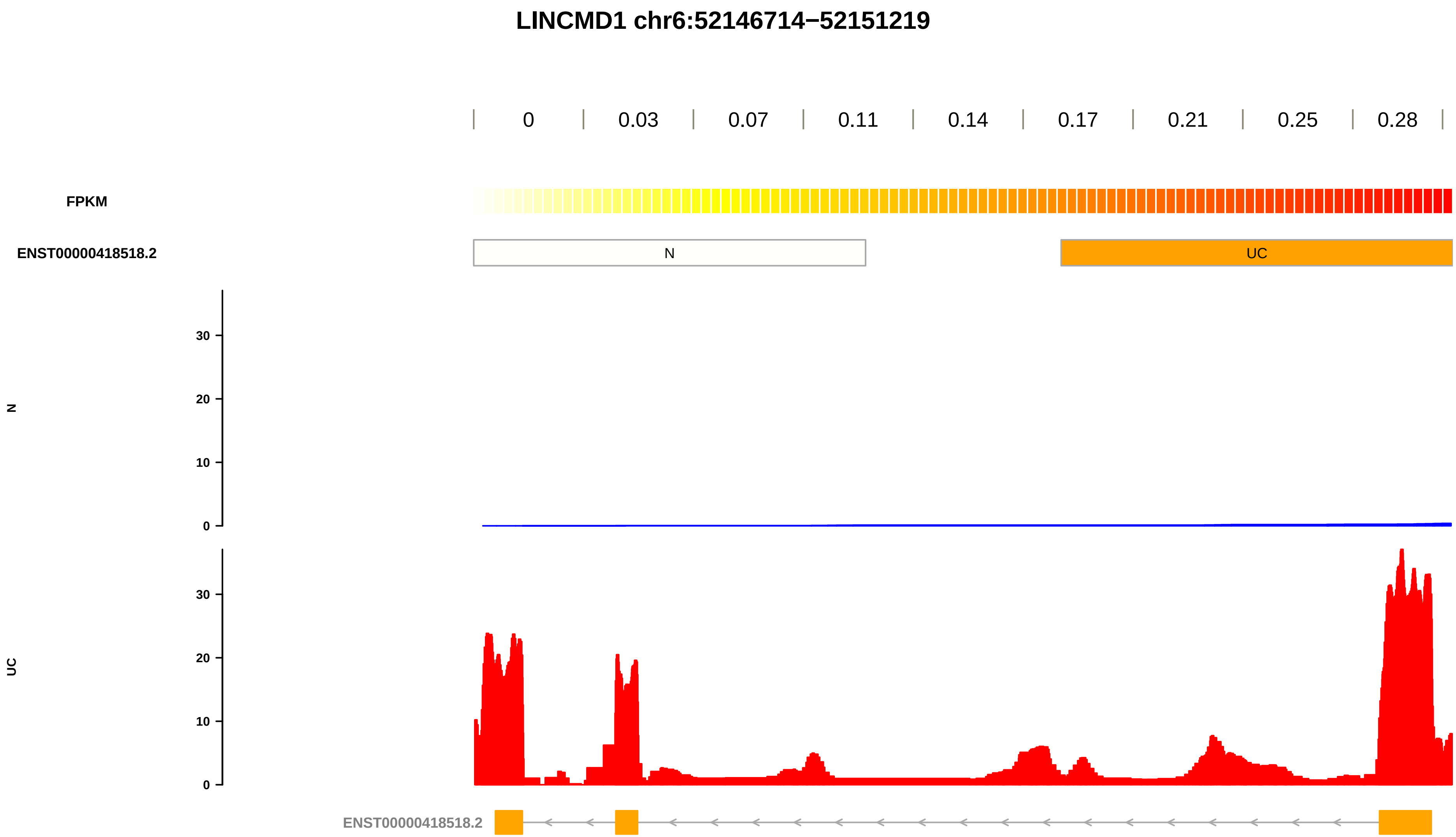

LINCMD1 chr6:52146714−52151219
0
0.03
0.07
0.11
0.14
0.17
0.21
0.25
0.28
FPKM
ENST00000418518.2
N
UC
N
30
20
10
0
UC
30
20
10
0
ENST00000418518.2

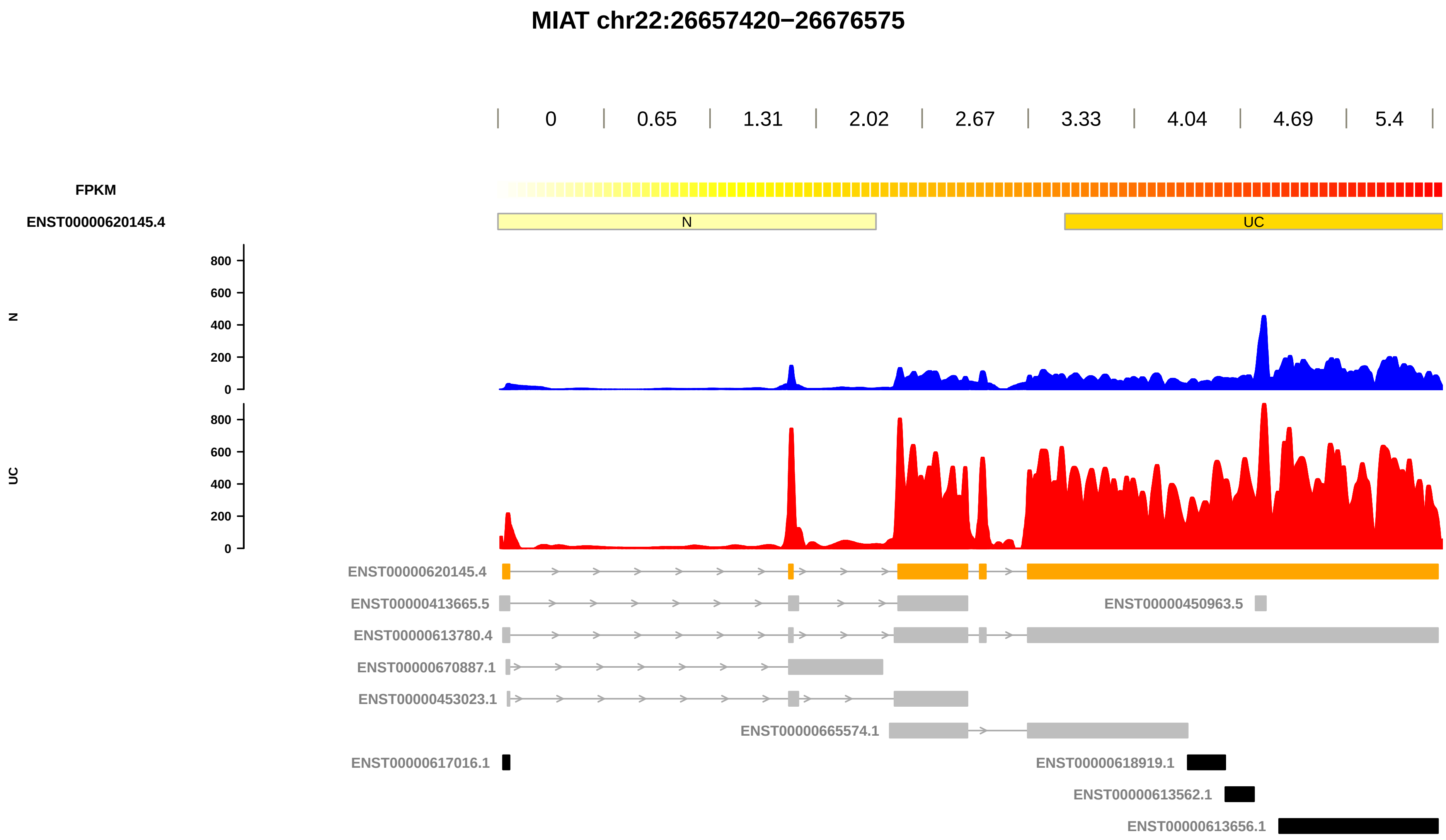
MIAT chr22:26657420−26676575
0
0.65
1.31
2.02
2.67
3.33
4.04
4.69
5.4
FPKM
ENST00000620145.4
N
UC
800
600
400
200
0
N
UC
ENST00000620145.4
ENST00000413665.5
ENST00000450963.5
ENST00000613780.4
ENST00000670887.1
ENST00000453023.1
ENST00000665574.1
ENST00000617016.1
ENST00000618919.1
ENST00000613562.1
ENST00000613656.1

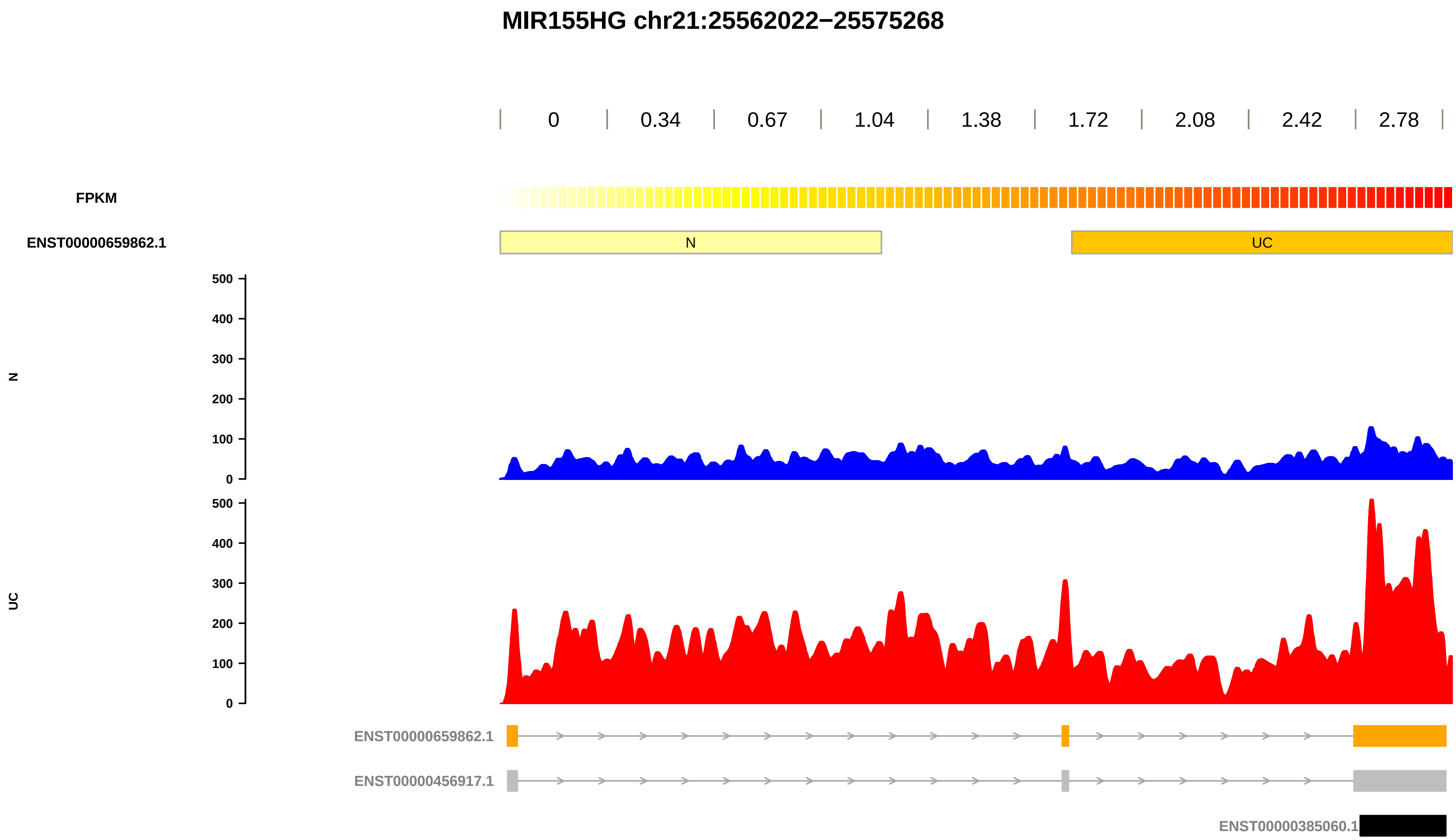
MIR155HG chr21:25562022−25575268
0
0.34
0.67
1.04
1.38
1.72
2.08
2.42
2.78
FPKM
ENST00000659862.1
N
UC
500
400
300
200
100
0
N
500
400
300
200
100
0
UC
ENST00000659862.1
ENST00000456917.1
ENST00000385060.1

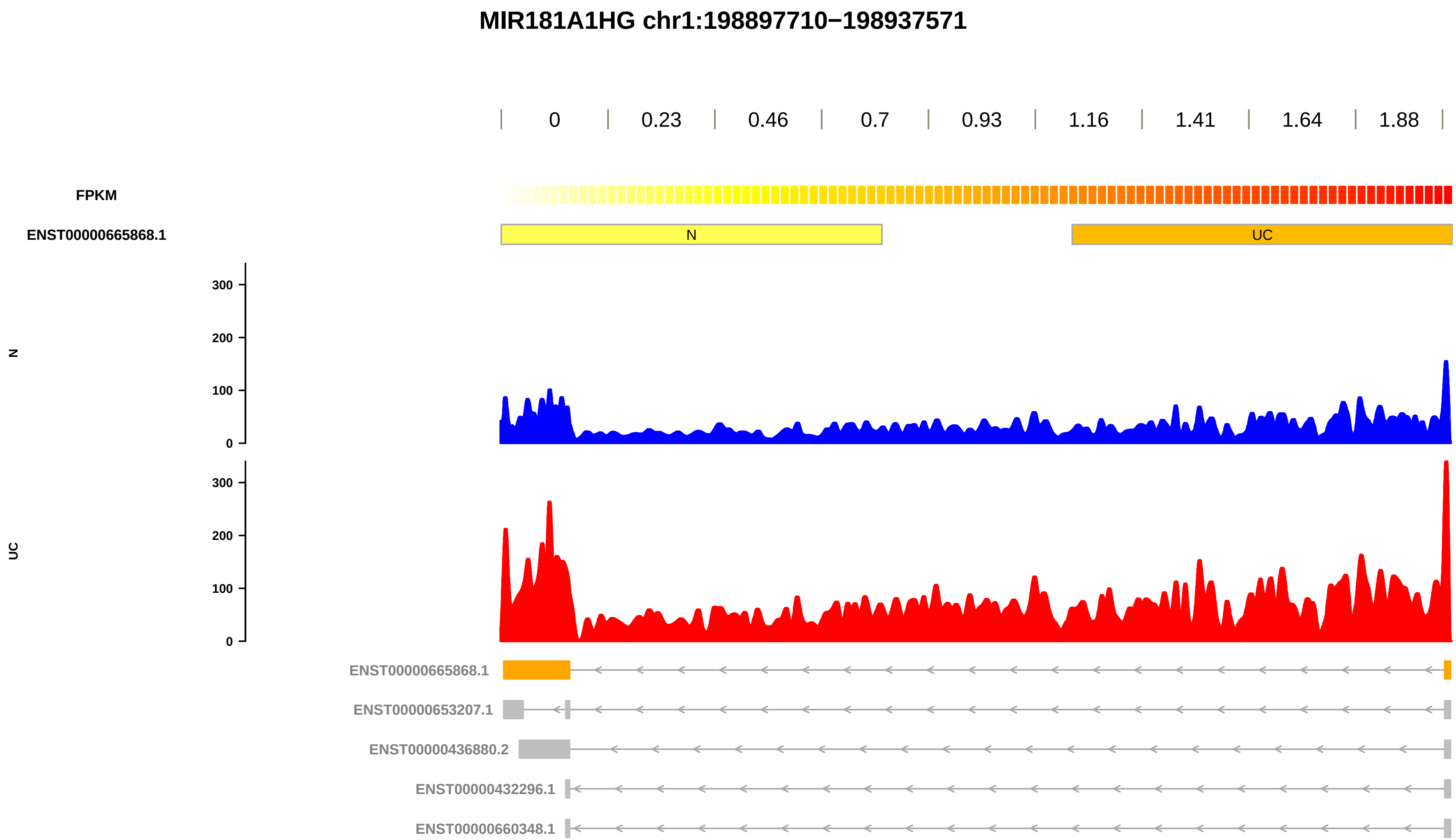
MIR181A1HG chr1:198897710−198937571
0
0.23
0.46
0.7
0.93
1.16
1.41
1.64
1.88
FPKM
ENST00000665868.1
N
UC
N
UC
300
200
100
0
300
200
100
0
ENST00000665868.1
ENST00000653207.1
ENST00000436880.2
ENST00000432296.1
ENST00000660348.1

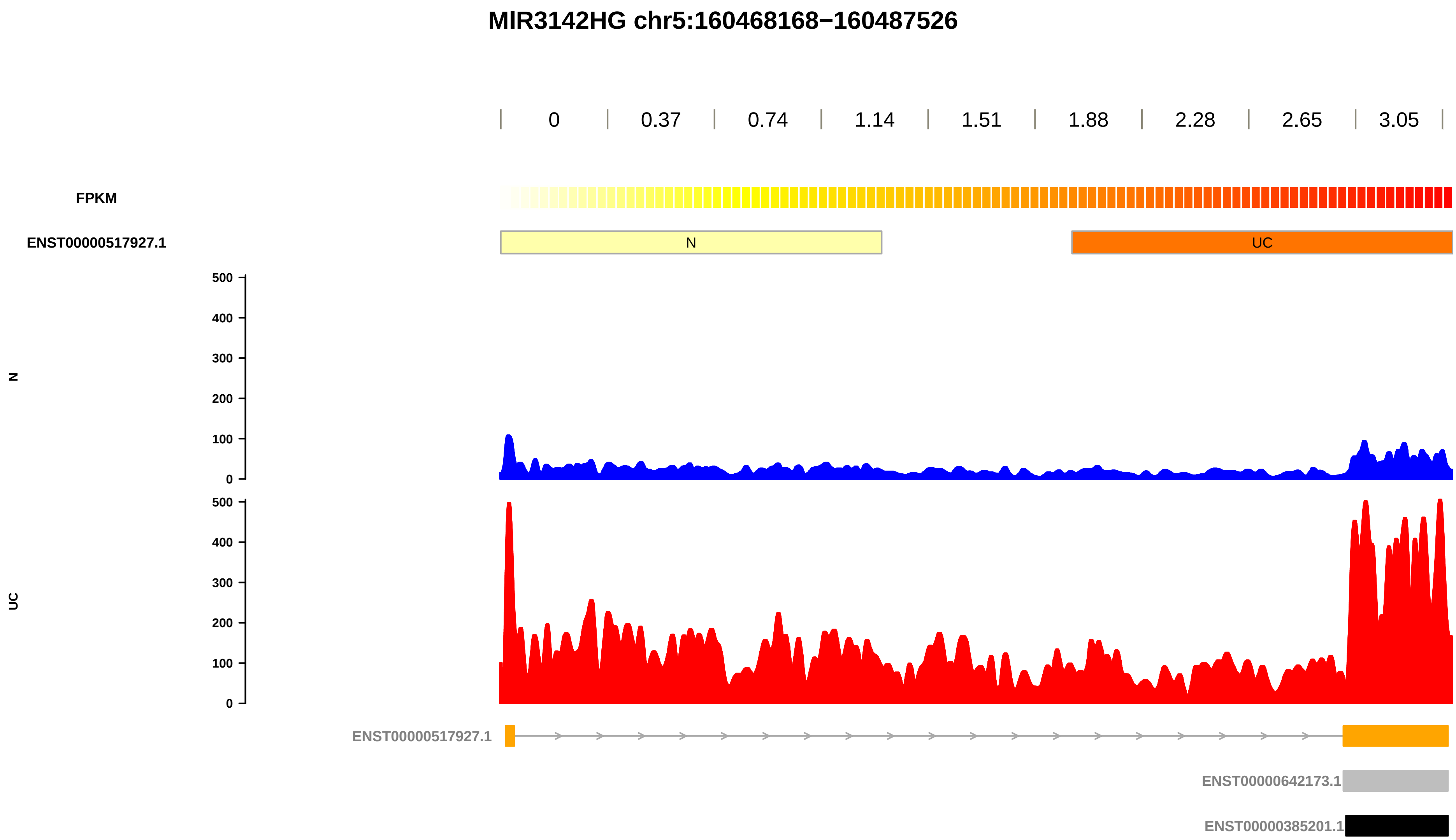
MIR3142HG chr5:160468168−160487526
0
0.37
0.74
1.14
1.51
1.88
2.28
2.65
3.05
FPKM
ENST00000517927.1
N
UC
N
UC
500
400
300
200
100
0
ENST00000517927.1
ENST00000642173.1
ENST00000385201.1

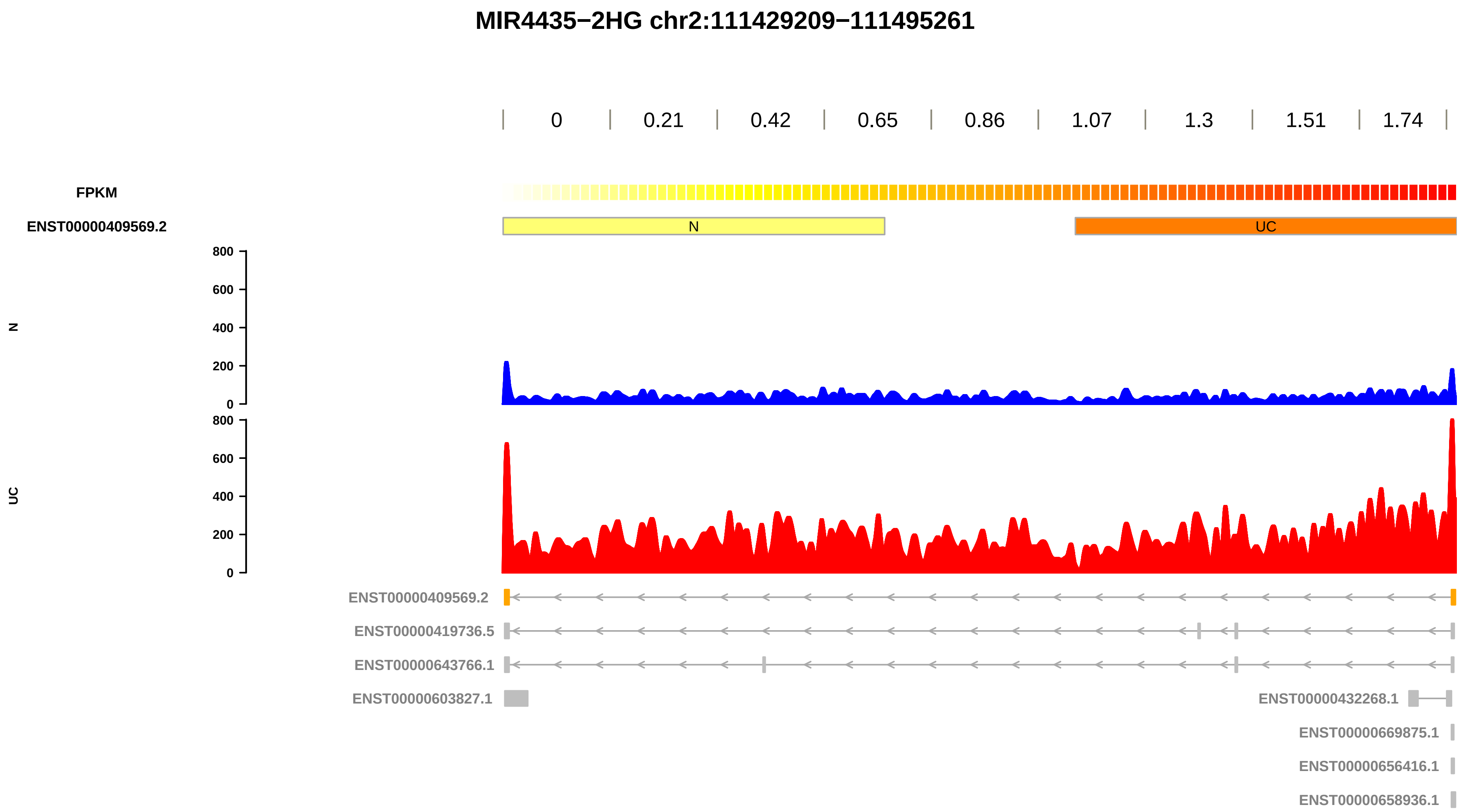
MIR4435−2HG chr2:111429209−111495261
0
0.21
0.42
0.65
0.86
1.07
1.3
1.51
1.74
FPKM
ENST00000409569.2
N
UC
800
600
400
200
0
N
UC
ENST00000409569.2
ENST00000419736.5
ENST00000643766.1
ENST00000603827.1
ENST00000432268.1
ENST00000669875.1
ENST00000656416.1
ENST00000658936.1

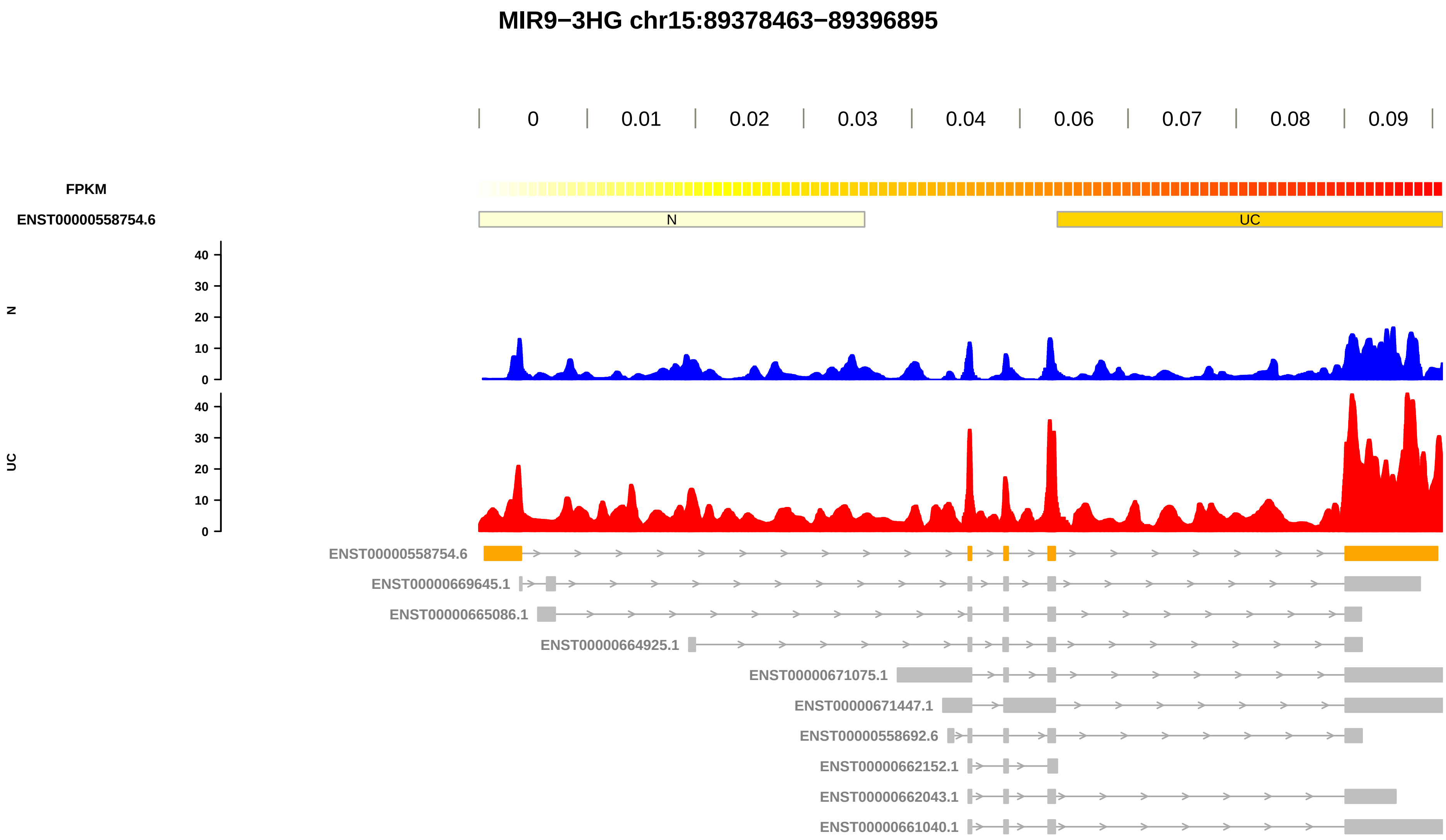
MIR9−3HG chr15:89378463−89396895
0
0.01
0.02
0.03
0.04
0.06
0.07
0.08
0.09
FPKM
ENST00000558754.6
N
UC
N
UC
0
10
20
30
40
ENST00000558754.6
ENST00000669645.1
ENST00000665086.1
ENST00000664925.1
ENST00000671075.1
ENST00000671447.1
ENST00000558692.6
ENST00000662152.1
ENST00000662043.1
ENST00000661040.1

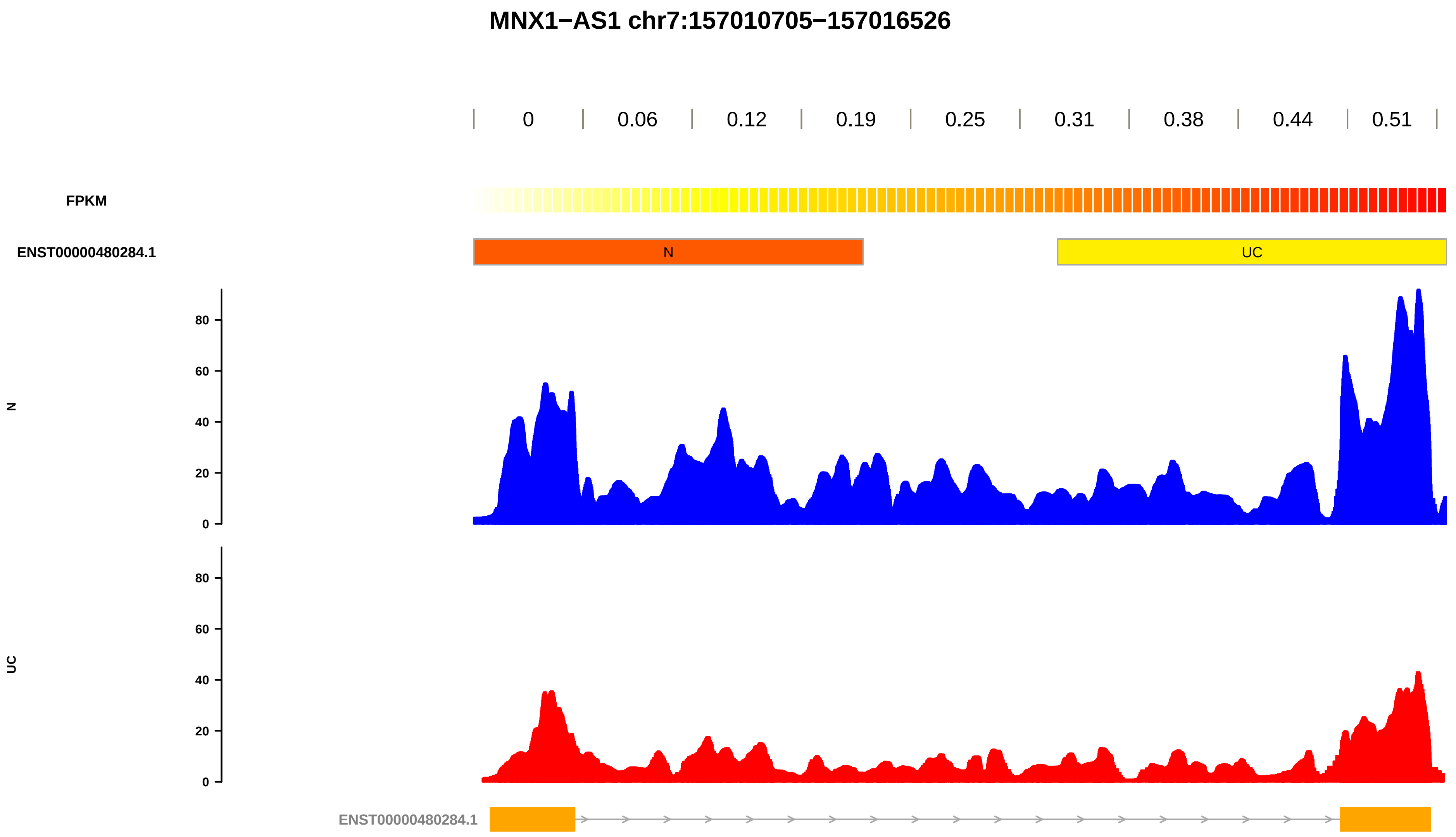
MNX1−AS1 chr7:157010705−157016526
0
0.06
0.12
0.19
0.25
0.31
0.38
0.44
0.51
FPKM
ENST00000480284.1
N
UC
N
UC
0
20
40
60
80
ENST00000480284.1

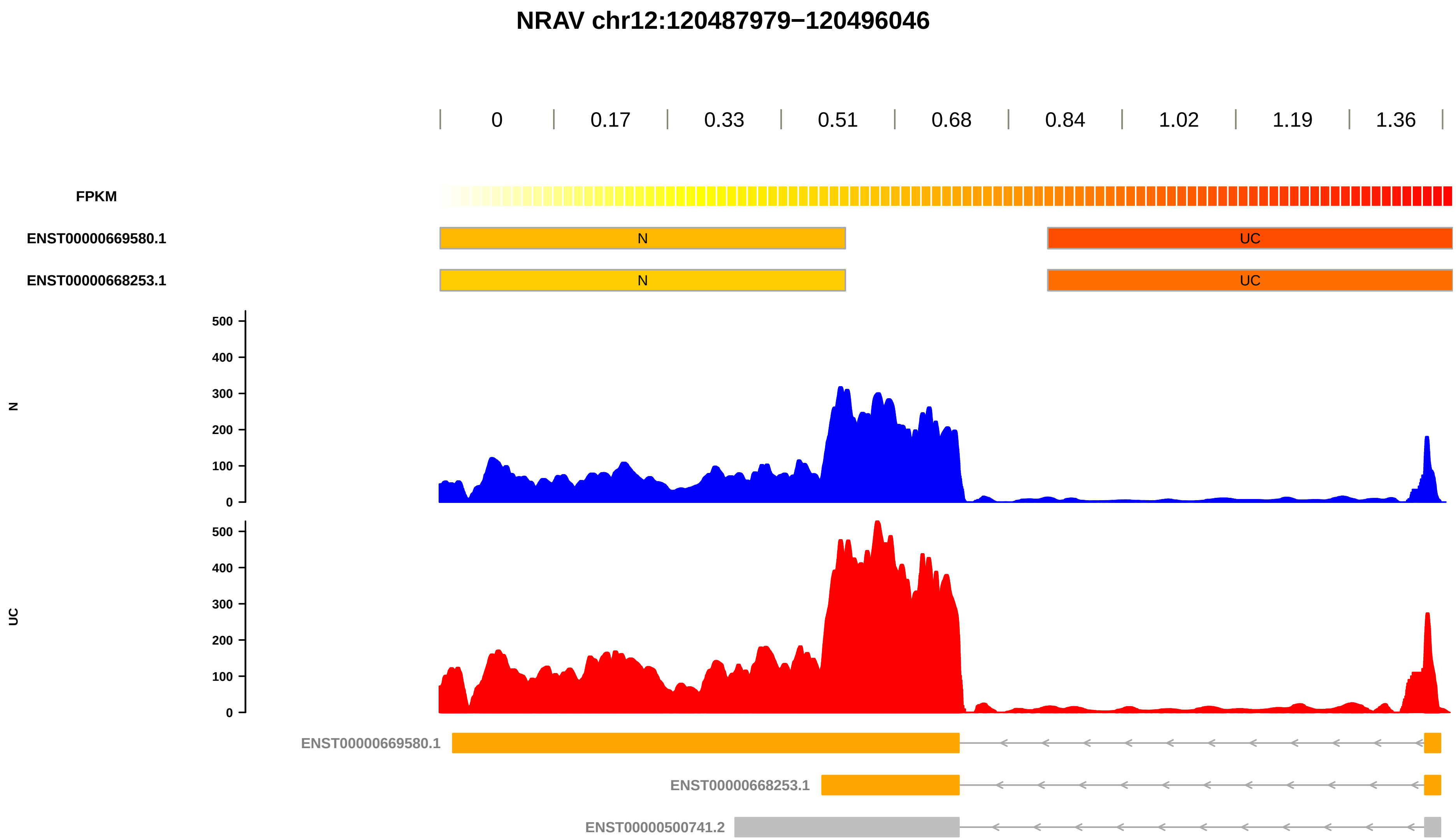

NRAV chr12:120487979−120496046
0
0.17
0.33
0.51
0.68
0.84
1.02
1.19
1.36
FPKM
ENST00000669580.1
N
UC
ENST00000668253.1
N
UC
N
UC
500
400
300
200
100
0
ENST00000669580.1
ENST00000668253.1
ENST00000500741.2

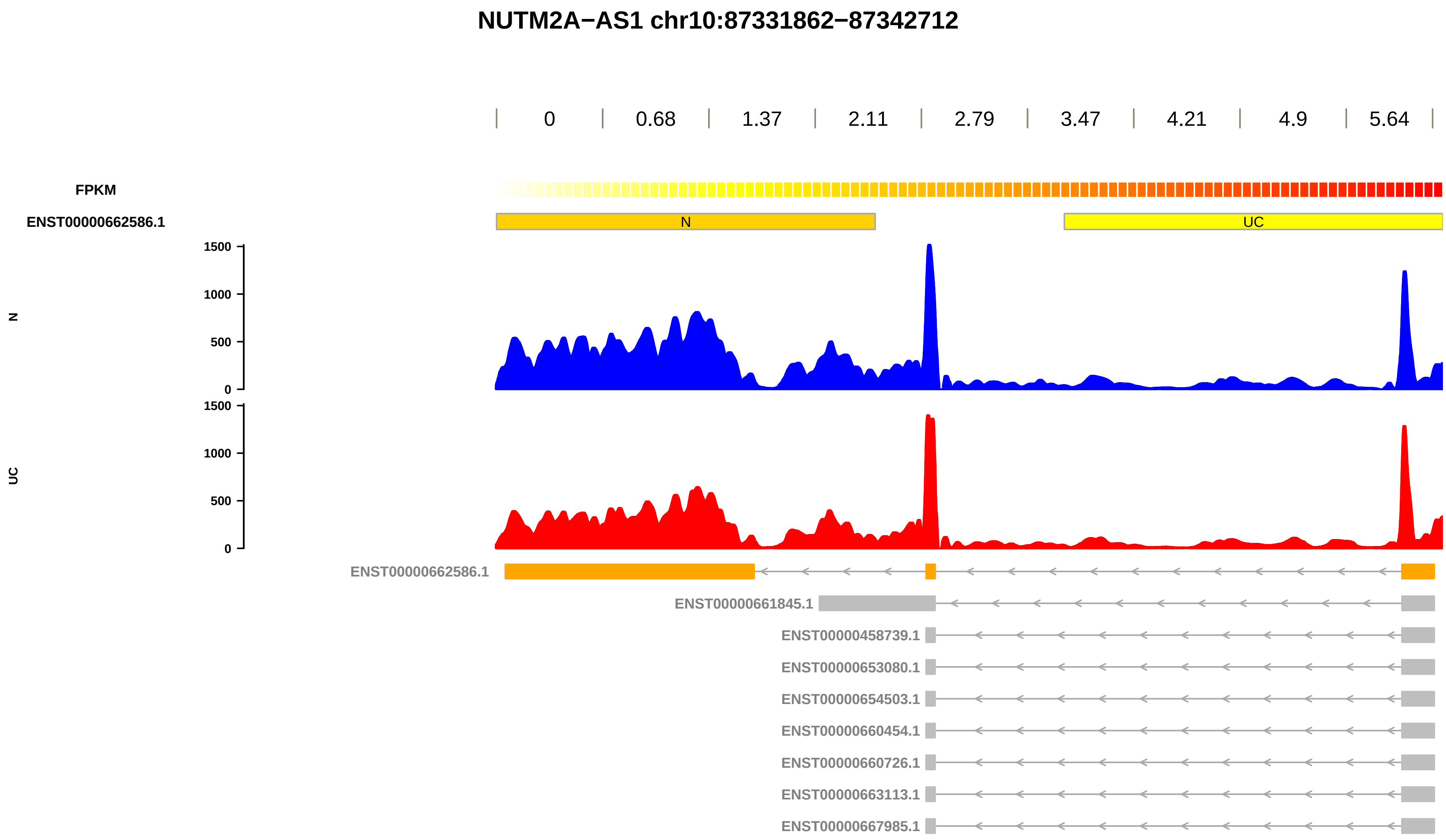
NUTM2A−AS1 chr10:87331862−87342712
0
0.68
1.37
2.11
2.79
3.47
4.21
4.9
5.64
FPKM
ENST00000662586.1
N
UC
1500
1000
500
0
N
1500
1000
500
0
UC
ENST00000662586.1
ENST00000661845.1
ENST00000458739.1
ENST00000653080.1
ENST00000654503.1
ENST00000660454.1
ENST00000660726.1
ENST00000663113.1
ENST00000667985.1

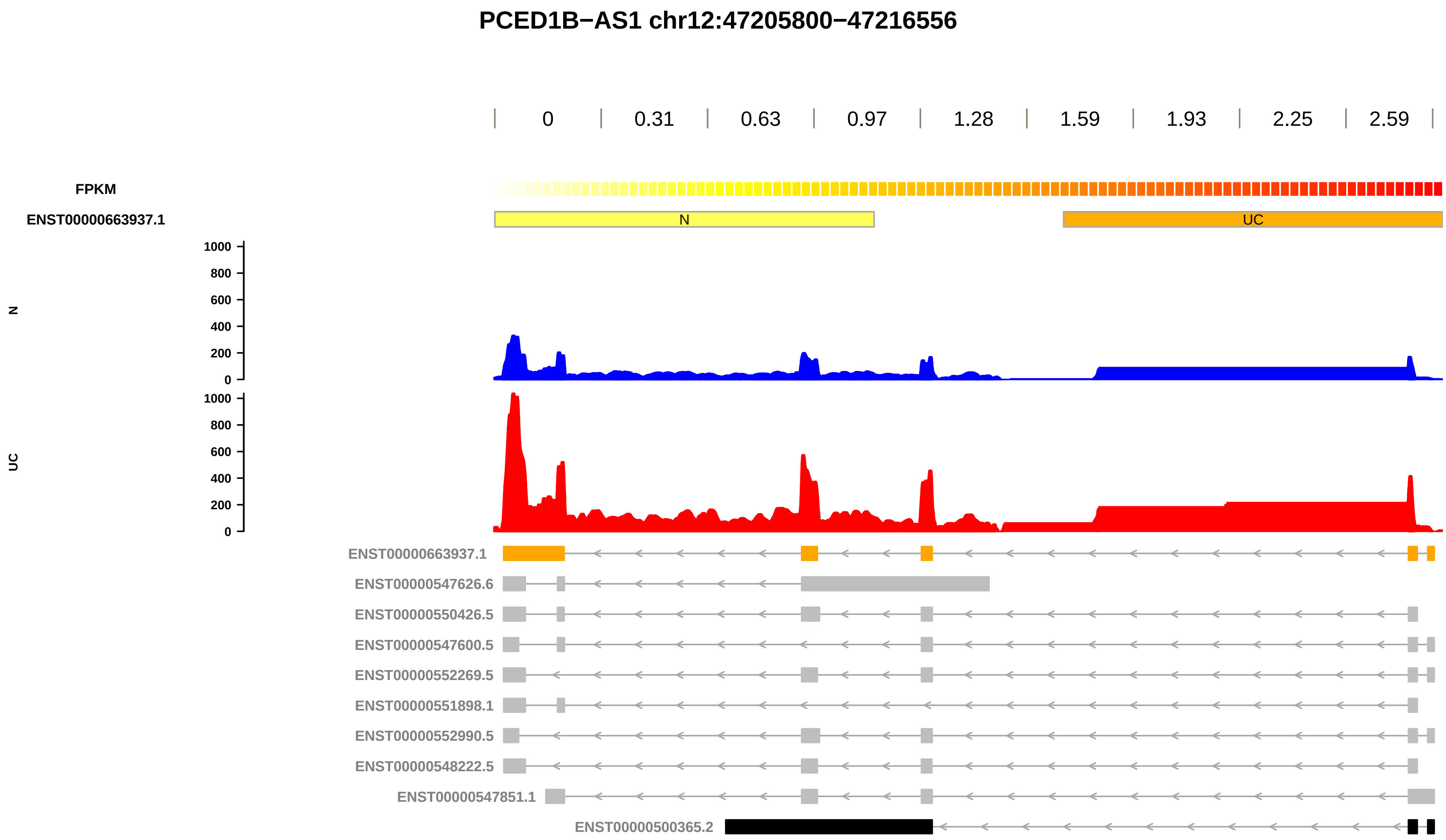
PCED1B−AS1 chr12:47205800−47216556
0
0.31
0.63
0.97
1.28
1.59
1.93
2.25
2.59
FPKM
ENST00000663937.1
N
UC
1000
800
600
400
200
0
N
1000
800
600
400
200
0
UC
ENST00000663937.1
ENST00000547626.6
ENST00000550426.5
ENST00000547600.5
ENST00000552269.5
ENST00000551898.1
ENST00000552990.5
ENST00000548222.5
ENST00000547851.1
ENST00000500365.2

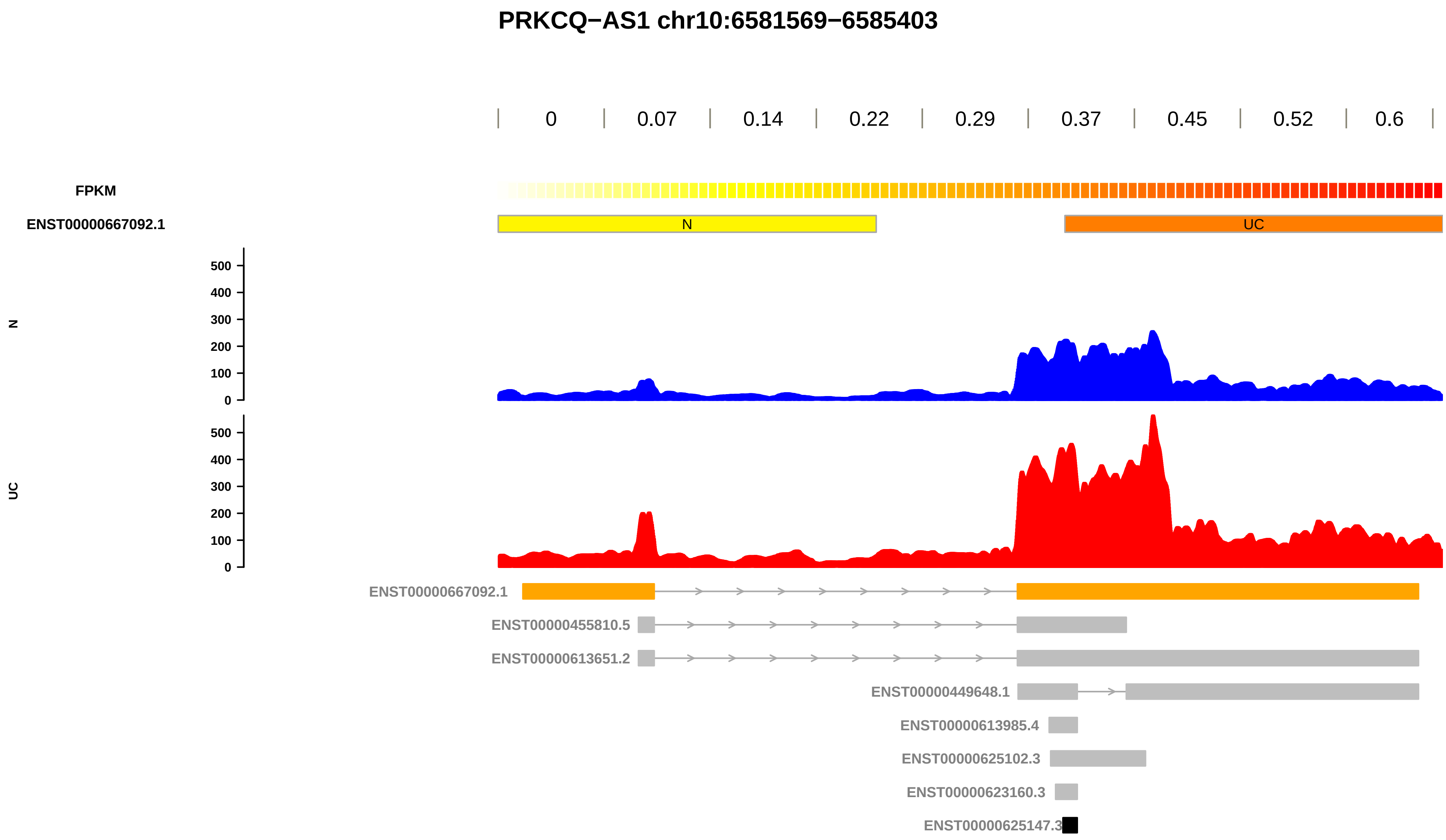

PRKCQ−AS1 chr10:6581569−6585403
0
0.07
0.14
0.22
0.29
0.37
0.45
0.52
0.6
FPKM
ENST00000667092.1
N
UC
N
UC
500
400
300
200
100
0
ENST00000667092.1
ENST00000455810.5
ENST00000613651.2
ENST00000449648.1
ENST00000613985.4
ENST00000625102.3
ENST00000623160.3
ENST00000625147.3

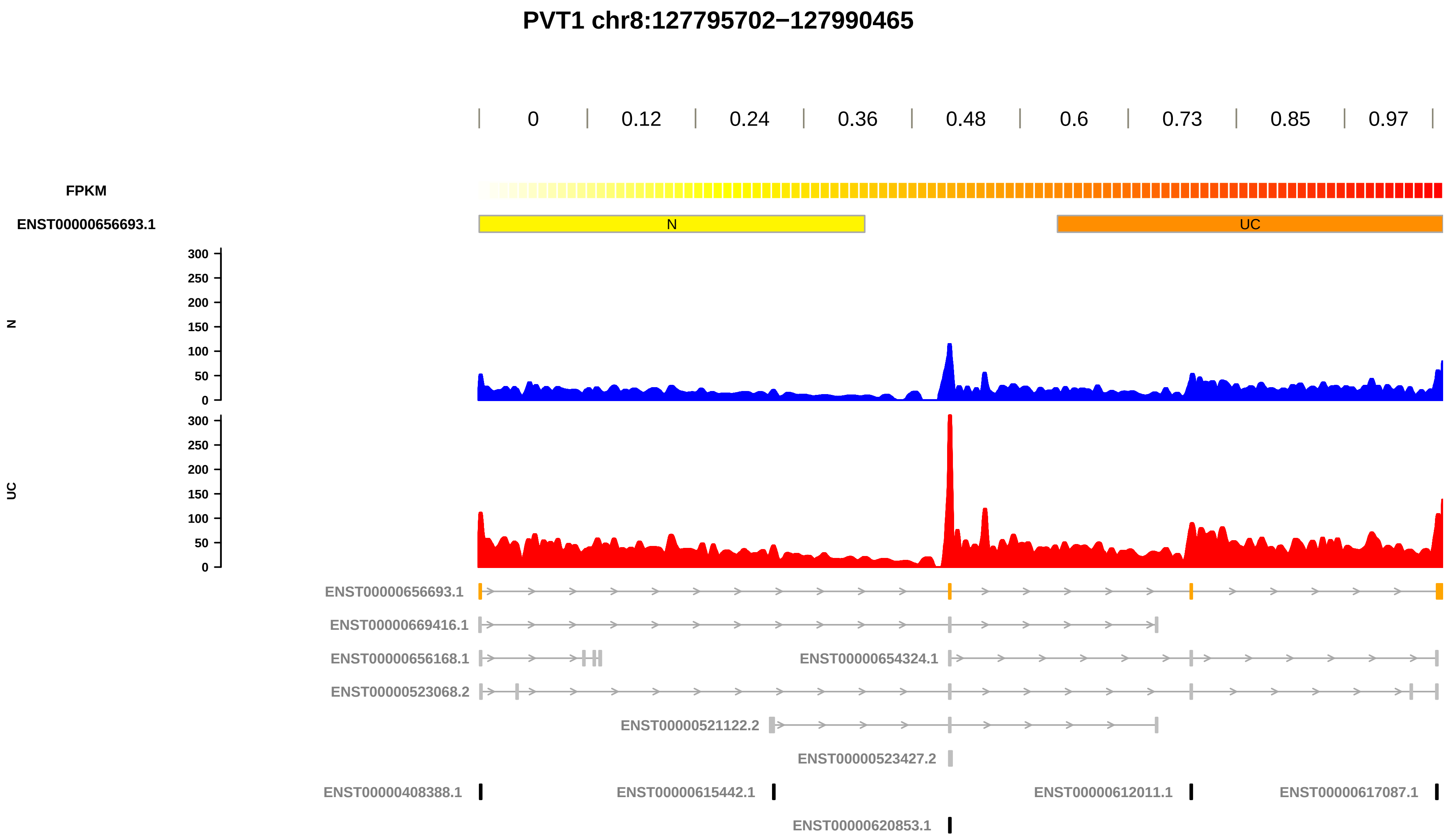
PVT1 chr8:127795702−127990465
0
0.12
0.24
0.36
0.48
0.6
0.73
0.85
0.97
FPKM
ENST00000656693.1
N
UC
300
250
200
150
100
50
0
N
UC
ENST00000656693.1
ENST00000669416.1
ENST00000656168.1
ENST00000654324.1
ENST00000523068.2
ENST00000521122.2
ENST00000523427.2
ENST00000408388.1
ENST00000615442.1
ENST00000612011.1
ENST00000617087.1
ENST00000620853.1

# SEMA6A−AS2 chr5:116574383−116611300

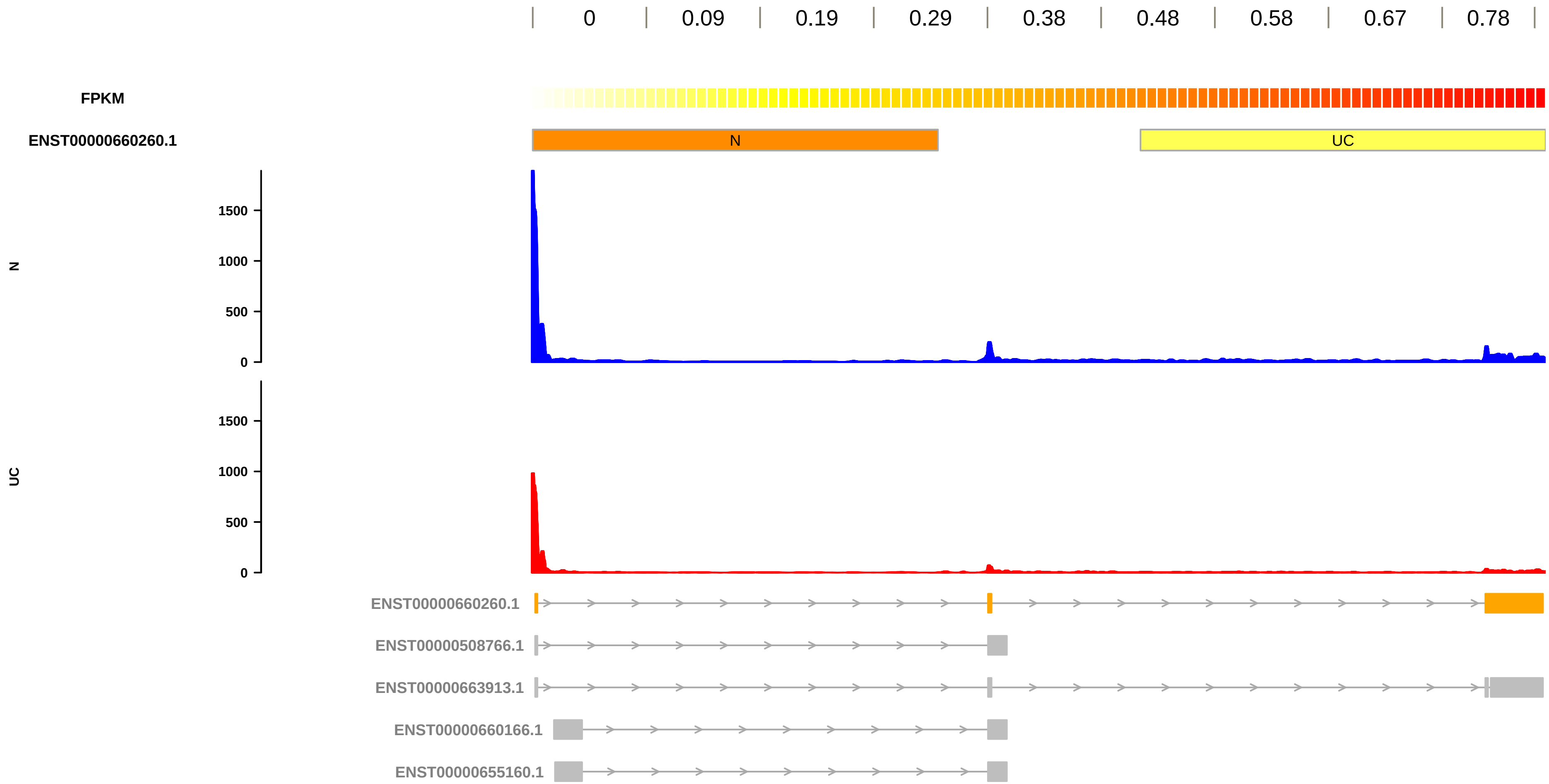

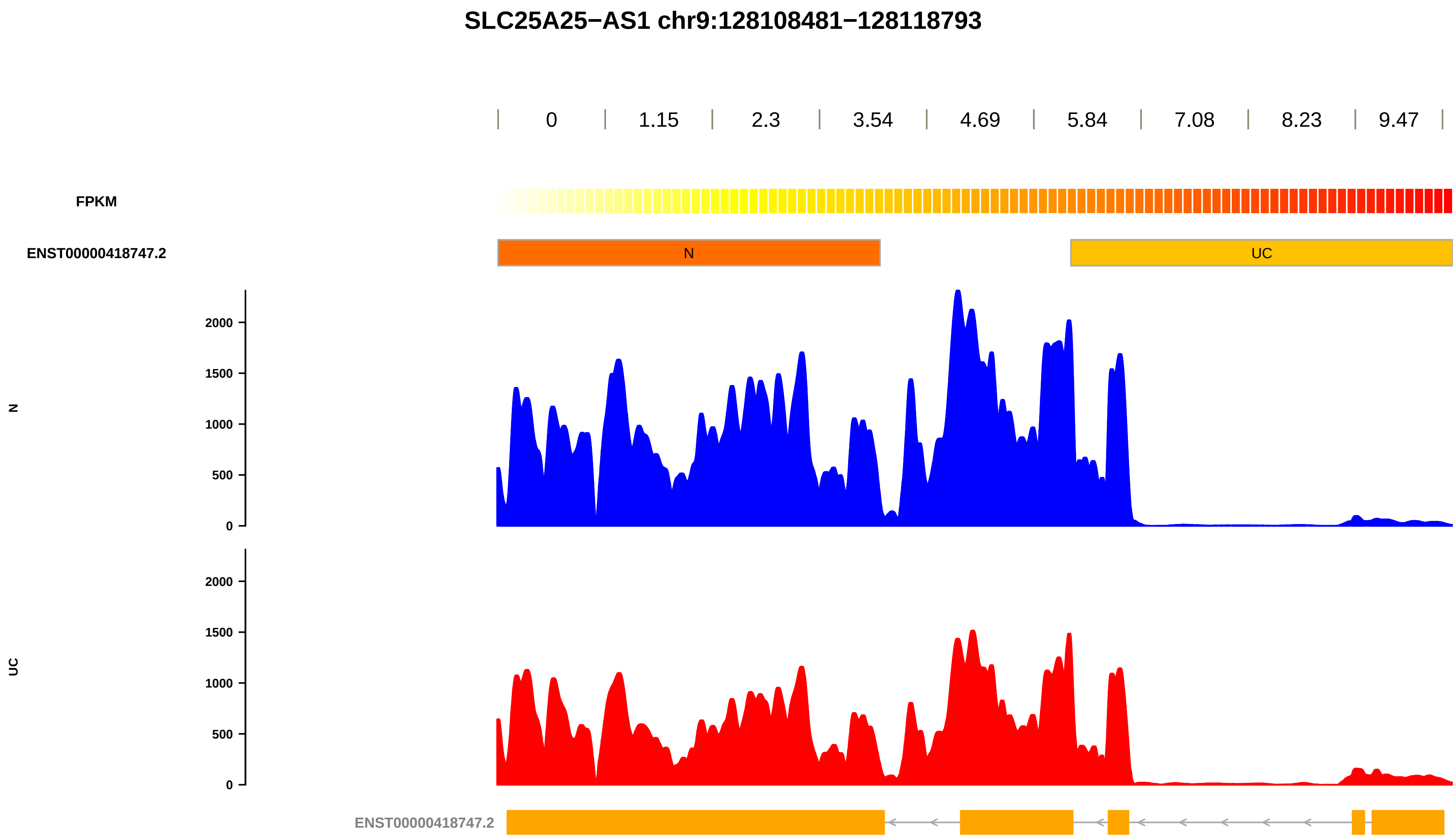
SLC25A25−AS1 chr9:128108481−128118793
0
1.15
2.3
3.54
4.69
5.84
7.08
8.23
9.47
FPKM
ENST00000418747.2
N
UC
2000
1500
1000
500
0
N
UC
ENST00000418747.2

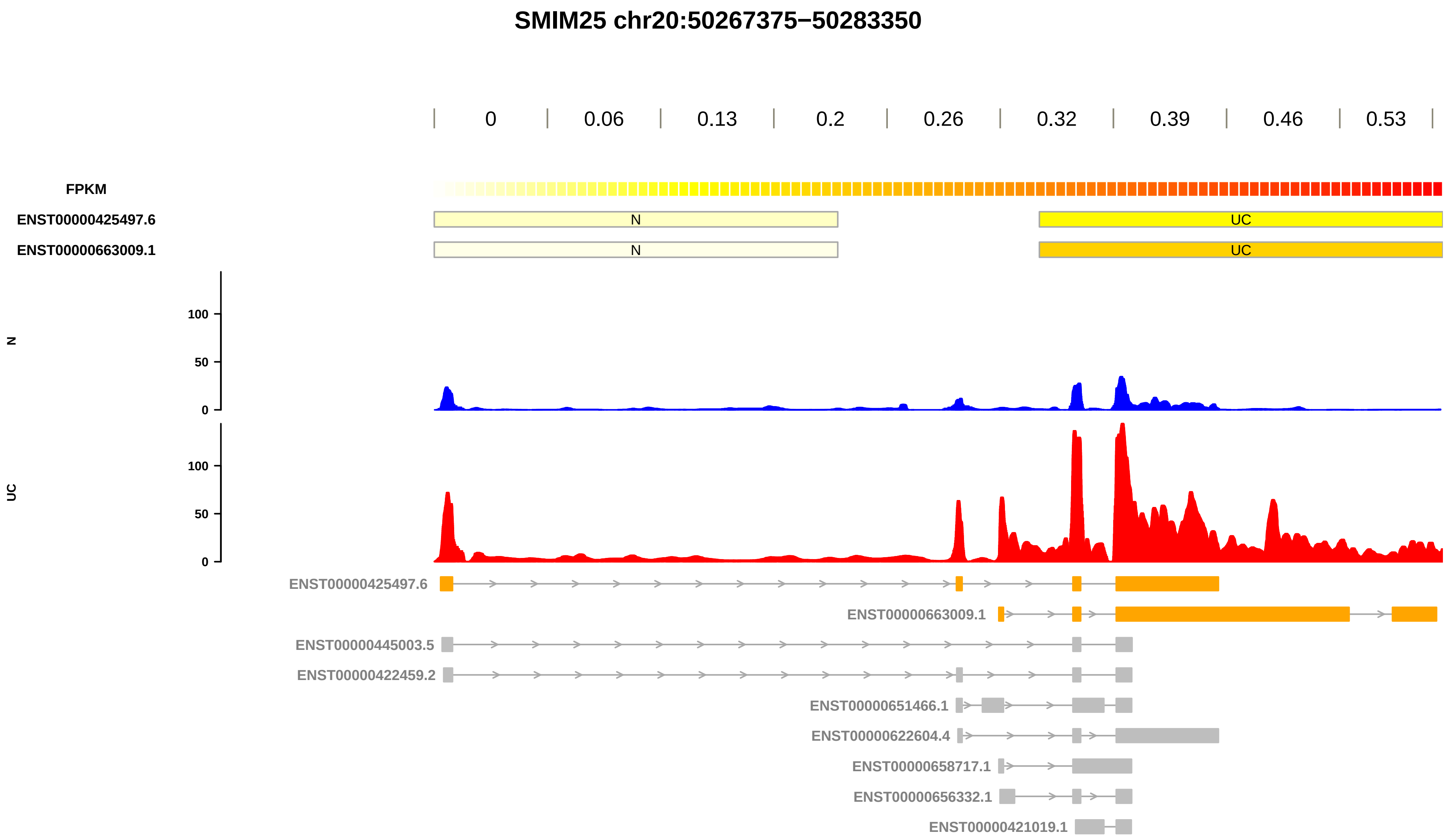
SMIM25 chr20:50267375−50283350
0
0.06
0.13
0.2
0.26
0.32
0.39
0.46
0.53
FPKM
ENST00000425497.6
ENST00000663009.1
N
UC
N
UC
N
100
50
0
UC
100
50
0
ENST00000425497.6
ENST00000663009.1
ENST00000445003.5
ENST00000422459.2
ENST00000651466.1
ENST00000622604.4
ENST00000658717.1
ENST00000656332.1
ENST00000421019.1

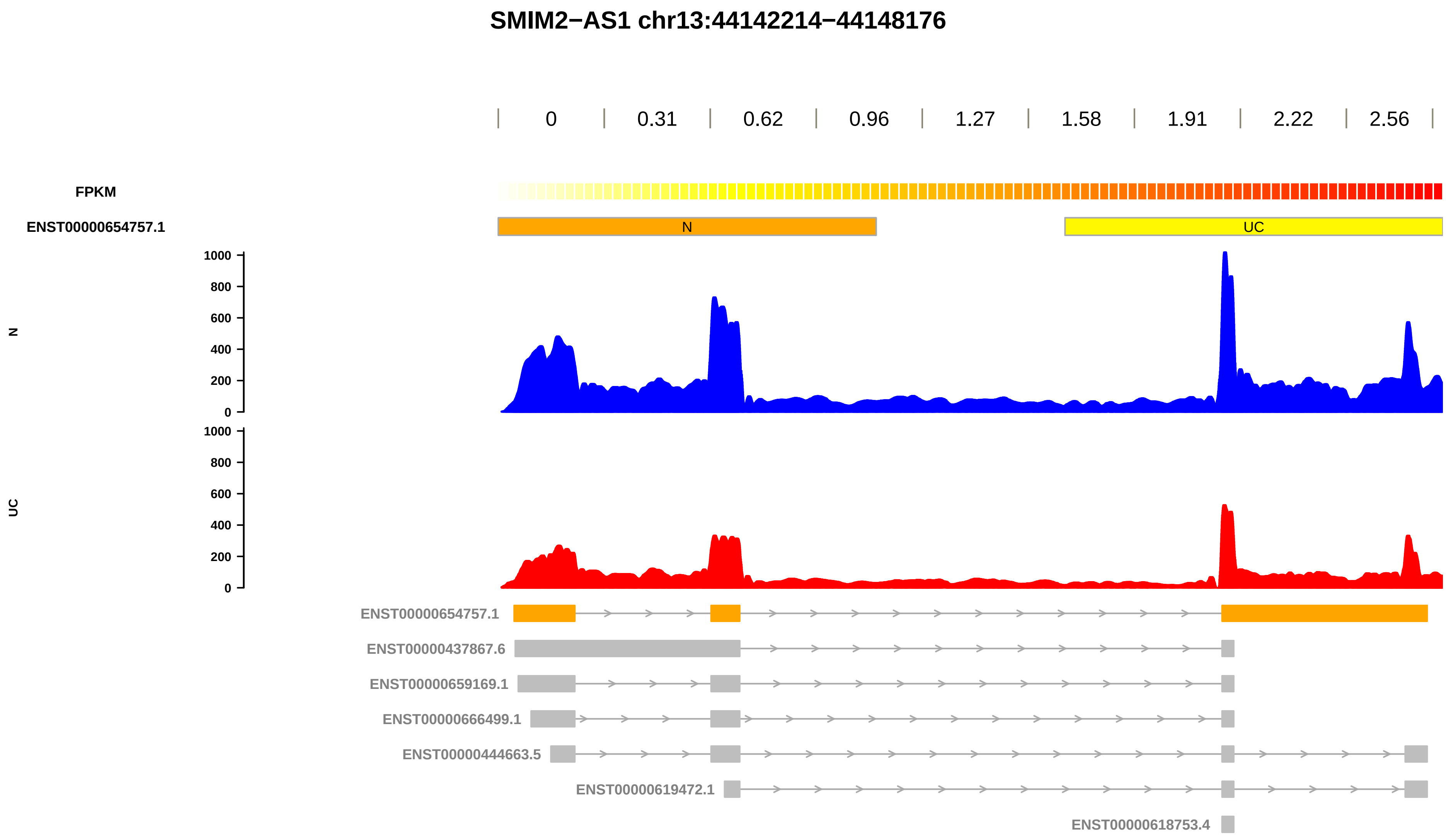
SMIM2−AS1 chr13:44142214−44148176
0
0.31
0.62
0.96
1.27
1.58
1.91
2.22
2.56
FPKM
ENST00000654757.1
N
UC
1000
800
600
400
200
0
N
1000
800
600
400
200
0
UC
ENST00000654757.1
ENST00000437867.6
ENST00000659169.1
ENST00000666499.1
ENST00000444663.5
ENST00000619472.1
ENST00000618753.4

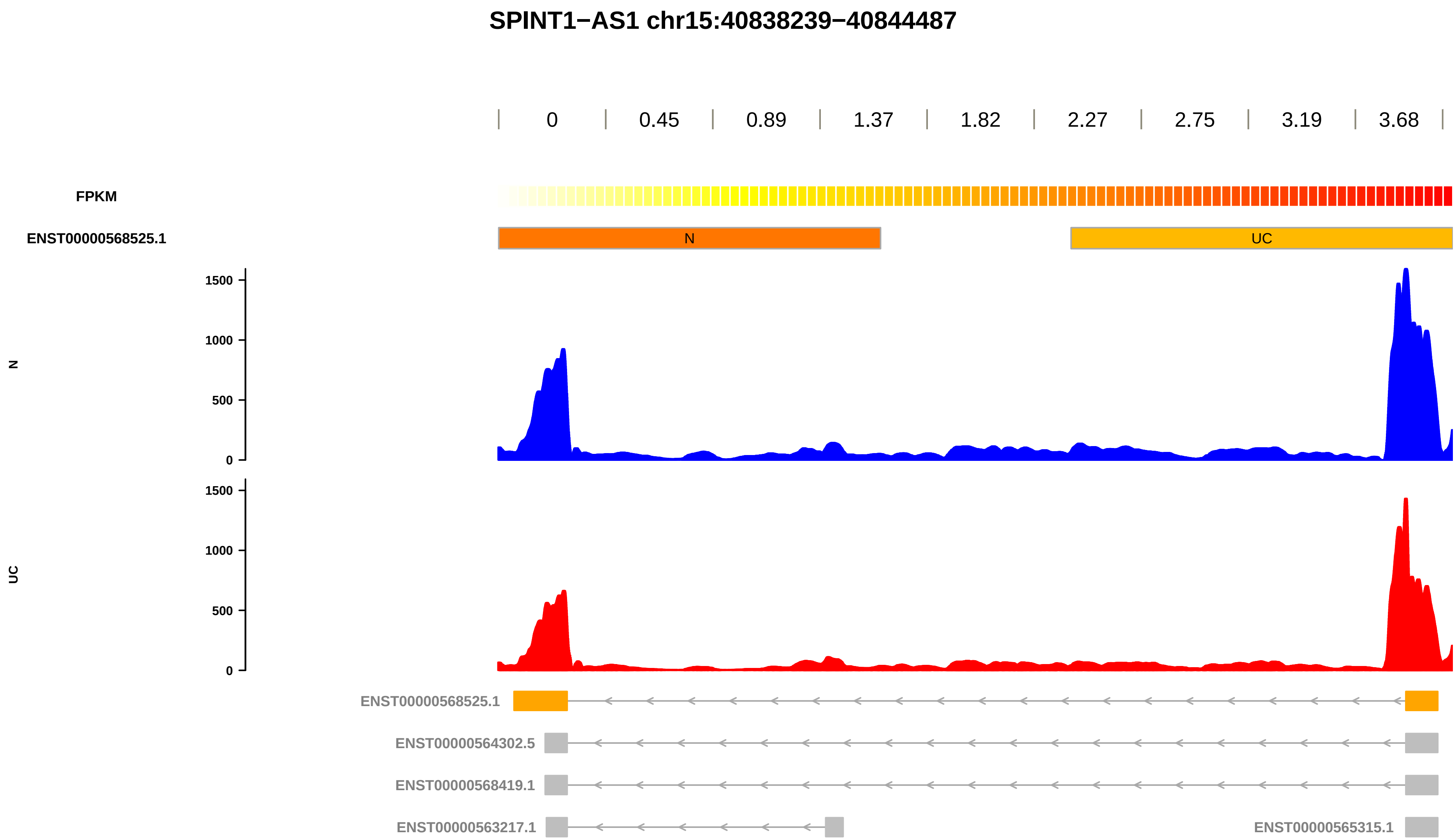

SPINT1−AS1 chr15:40838239−40844487
0
0.45
0.89
1.37
1.82
2.27
2.75
3.19
3.68
FPKM
ENST00000568525.1
N
UC
1500
1000
500
0
N
UC
ENST00000568525.1
ENST00000564302.5
ENST00000568419.1
ENST00000563217.1
ENST00000565315.1

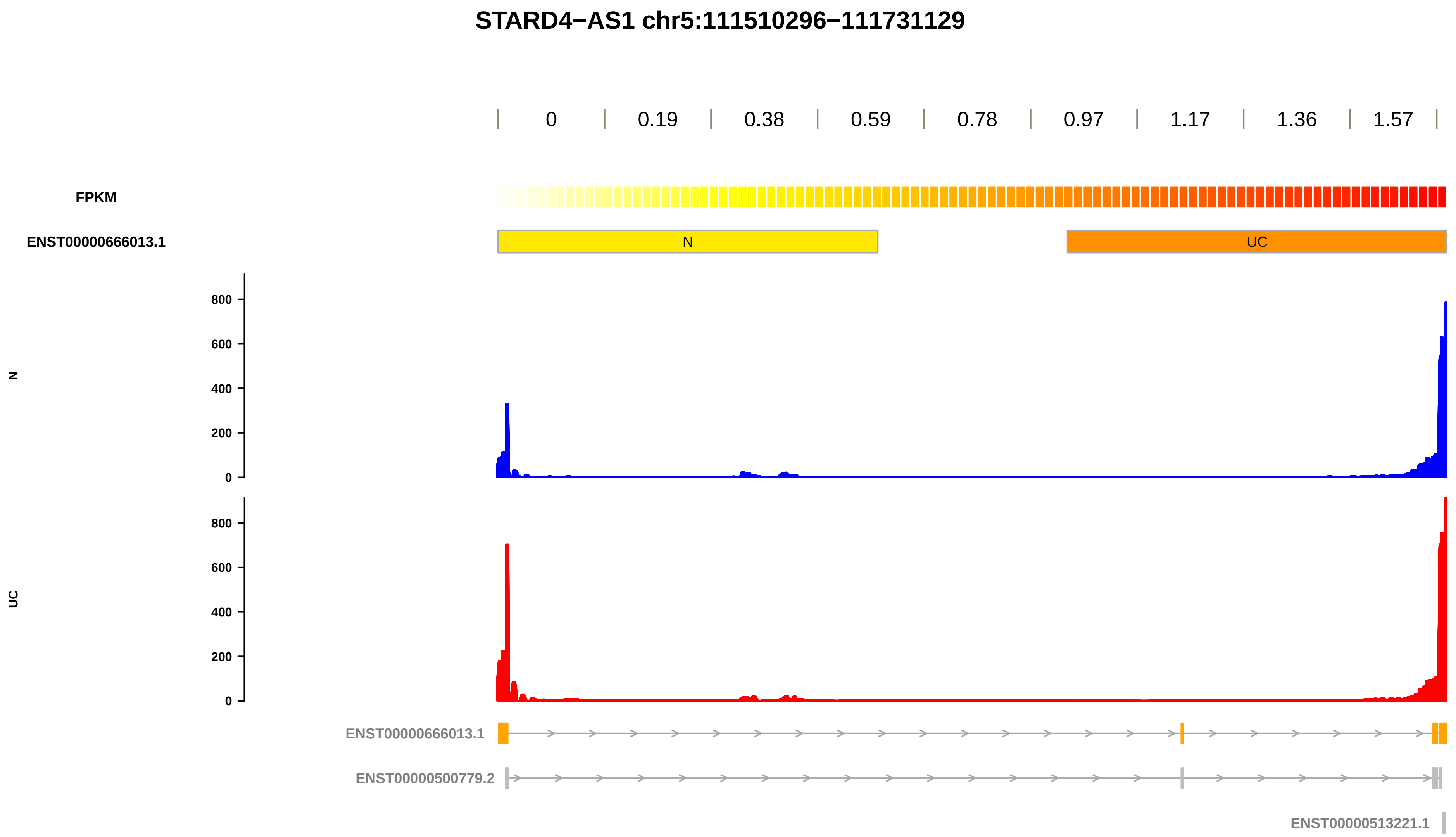

STARD4−AS1 chr5:111510296−111731129
0
0.19
0.38
0.59
0.78
0.97
1.17
1.36
1.57
FPKM
ENST00000666013.1
N
UC
N
800
600
400
200
0
UC
800
600
400
200
0
ENST00000666013.1
ENST00000500779.2
ENST00000513221.1

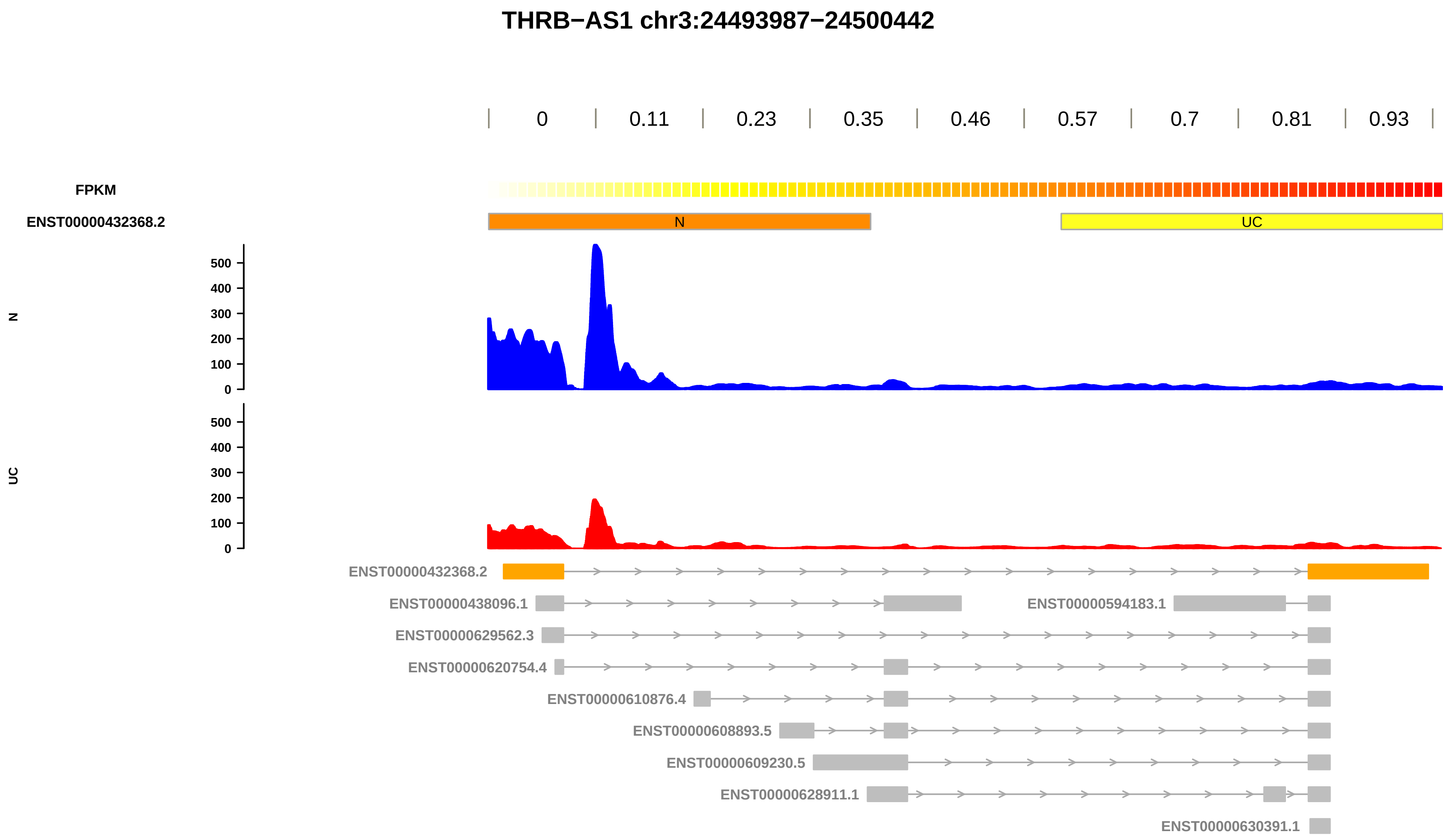

THRB−AS1 chr3:24493987−24500442
0
0.11
0.23
0.35
0.46
0.57
0.7
0.81
0.93
FPKM
ENST00000432368.2
N
UC
N
UC
500
400
300
200
100
0
ENST00000432368.2
ENST00000438096.1
ENST00000594183.1
ENST00000629562.3
ENST00000620754.4
ENST00000610876.4
ENST00000608893.5
ENST00000609230.5
ENST00000628911.1
ENST00000630391.1

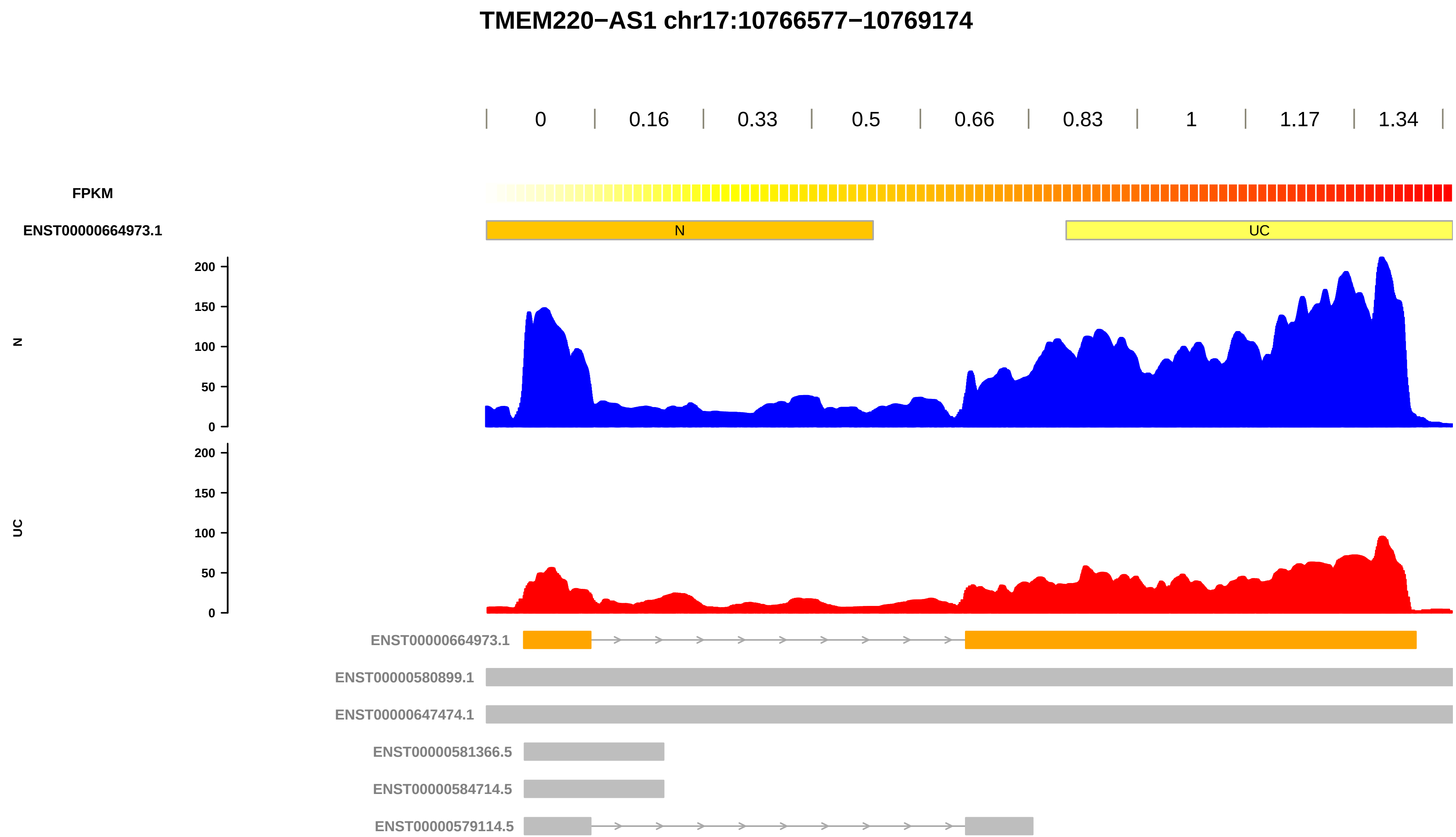
TMEM220−AS1 chr17:10766577−10769174
0
0.16
0.33
0.5
0.66
0.83
1
1.17
1.34
FPKM
ENST00000664973.1
N
UC
200
150
100
50
0
N
200
150
100
50
0
UC
ENST00000664973.1
ENST00000580899.1
ENST00000647474.1
ENST00000581366.5
ENST00000584714.5
ENST00000579114.5

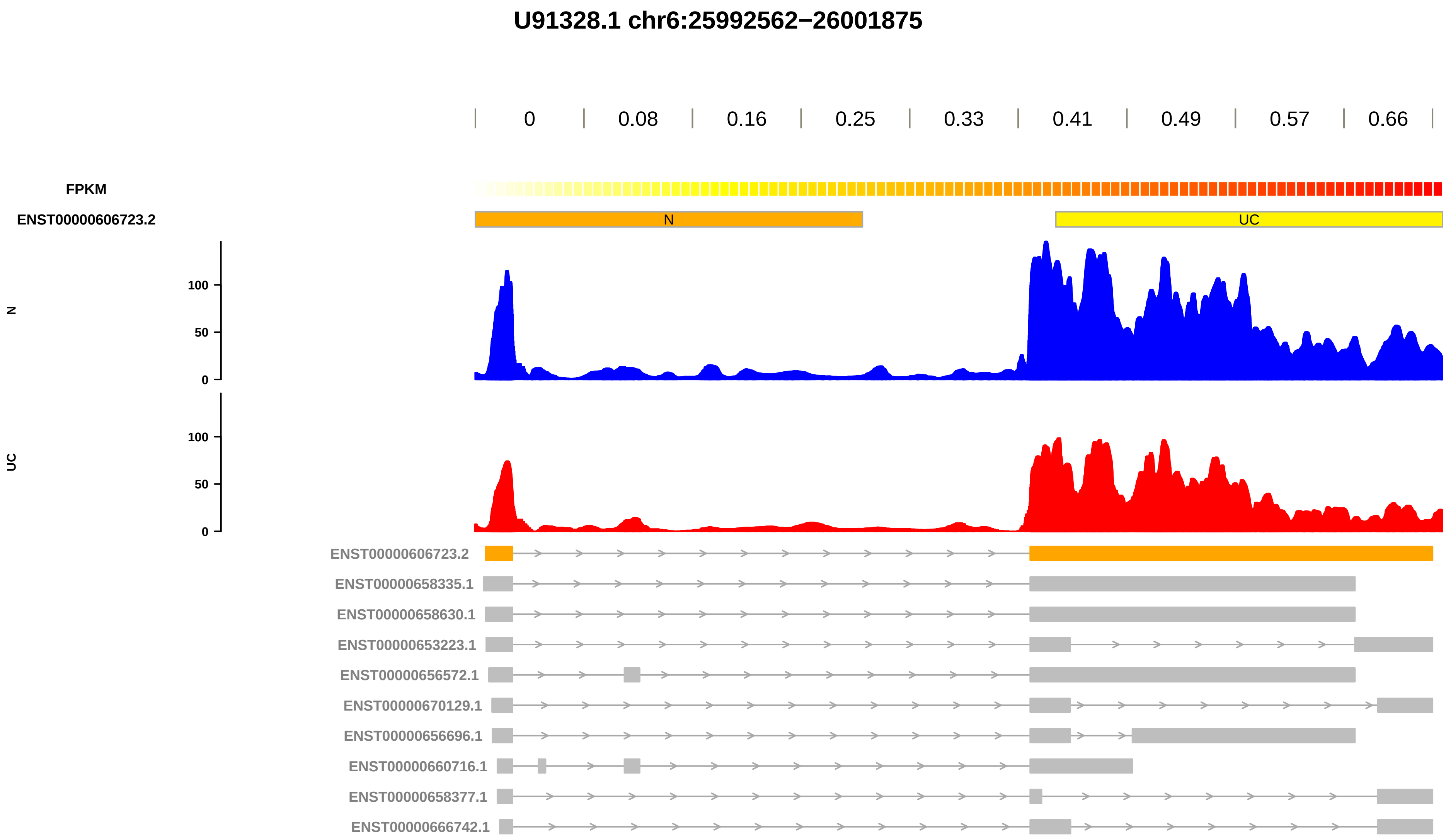

U91328.1 chr6:25992562−26001875
0
0.08
0.16
0.25
0.33
0.41
0.49
0.57
0.66
FPKM
ENST00000606723.2
N
UC
N
100
50
0
UC
100
50
0
ENST00000606723.2
ENST00000658335.1
ENST00000658630.1
ENST00000653223.1
ENST00000656572.1
ENST00000670129.1
ENST00000656696.1
ENST00000660716.1
ENST00000658377.1
ENST00000666742.1

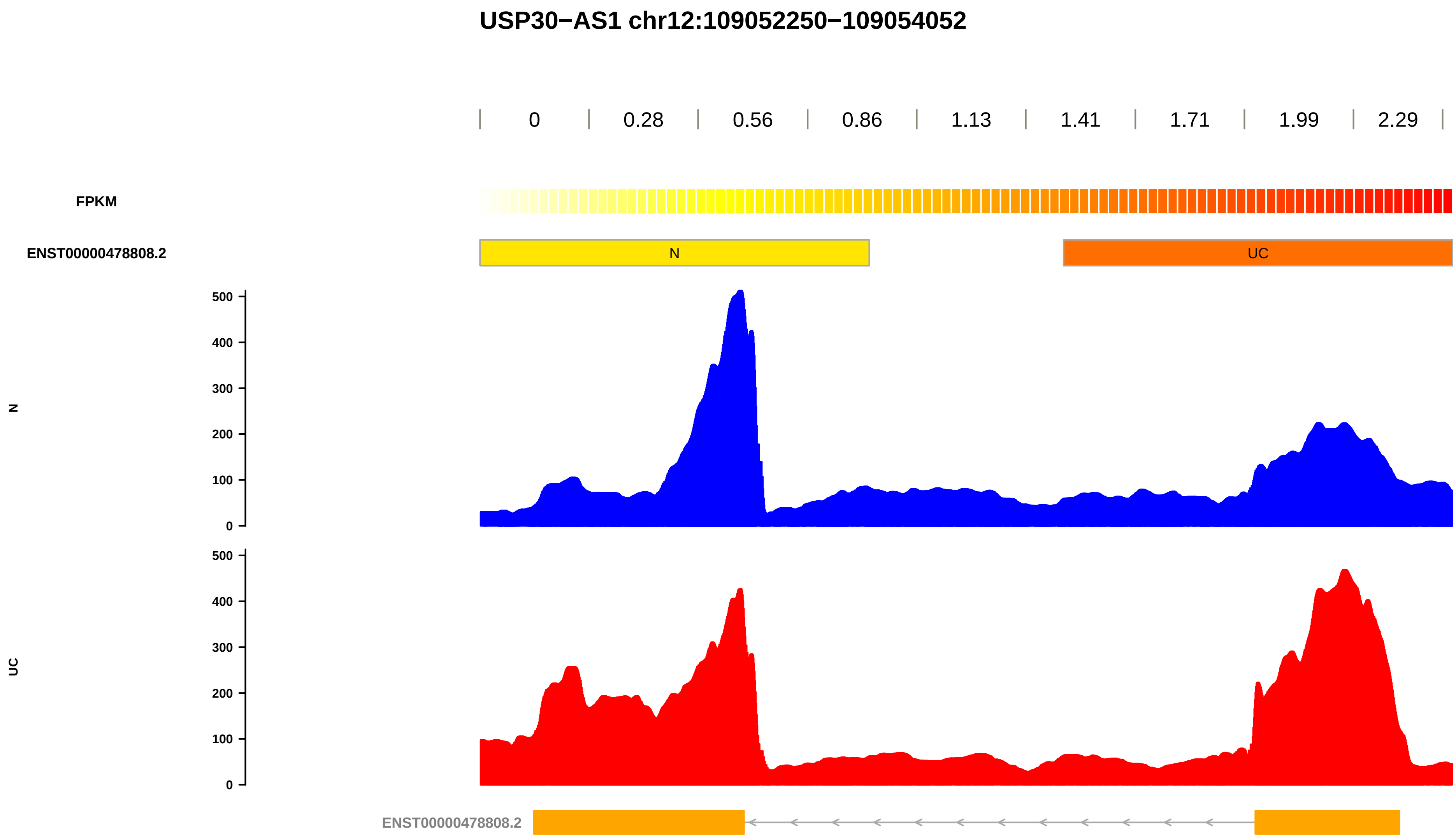

USP30−AS1 chr12:109052250−109054052
0
0.28
0.56
0.86
1.13
1.41
1.71
1.99
2.29
FPKM
ENST00000478808.2
N
UC
N
500
400
300
200
100
0
UC
500
400
300
200
100
0
ENST00000478808.2

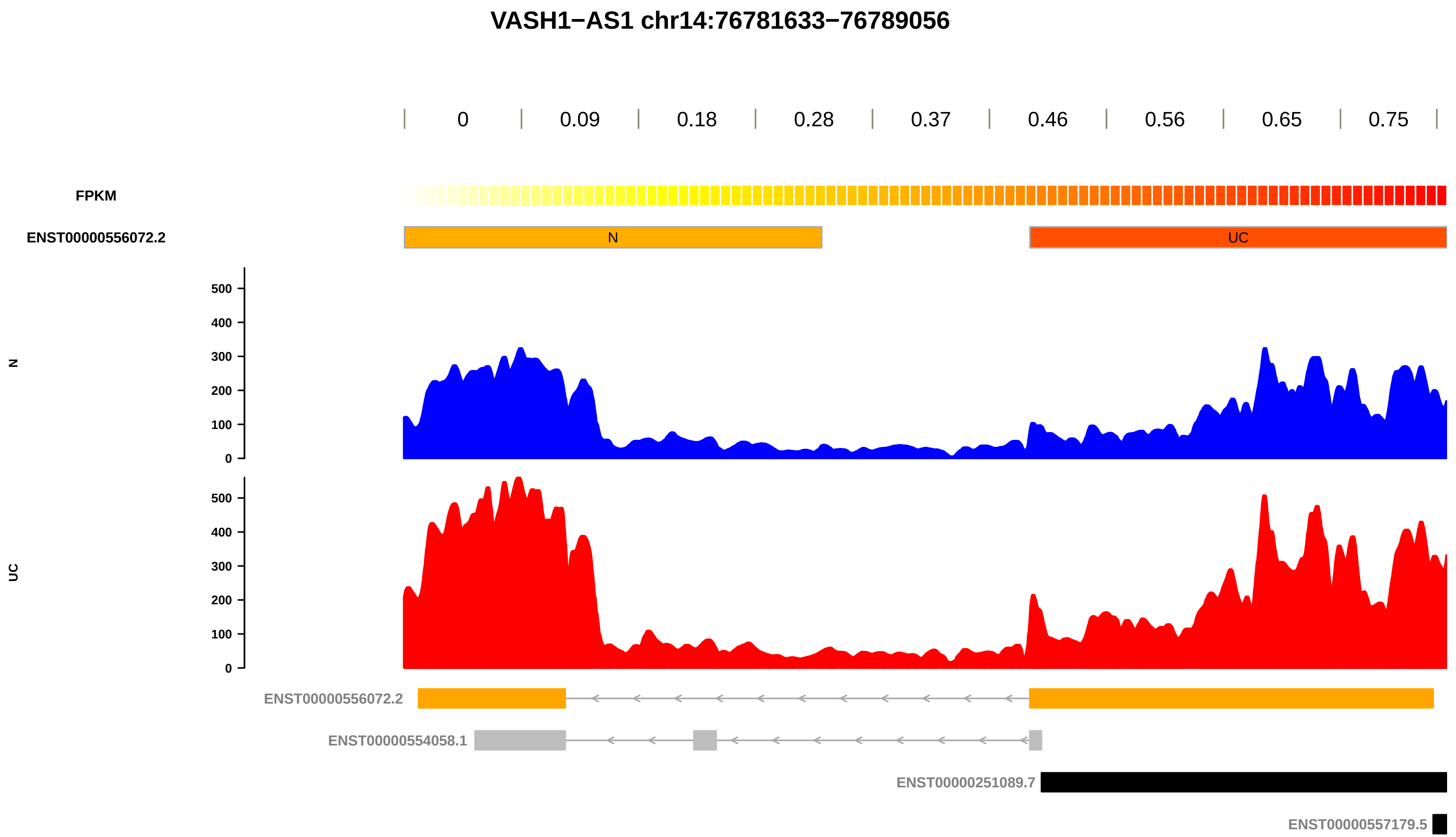
VASH1−AS1 chr14:76781633−76789056
0
0.09
0.18
0.28
0.37
0.46
0.56
0.65
0.75
FPKM
ENST00000556072.2
N
UC
500
400
300
200
100
0
N
UC
ENST00000556072.2
ENST00000554058.1
ENST00000251089.7
ENST00000557179.5

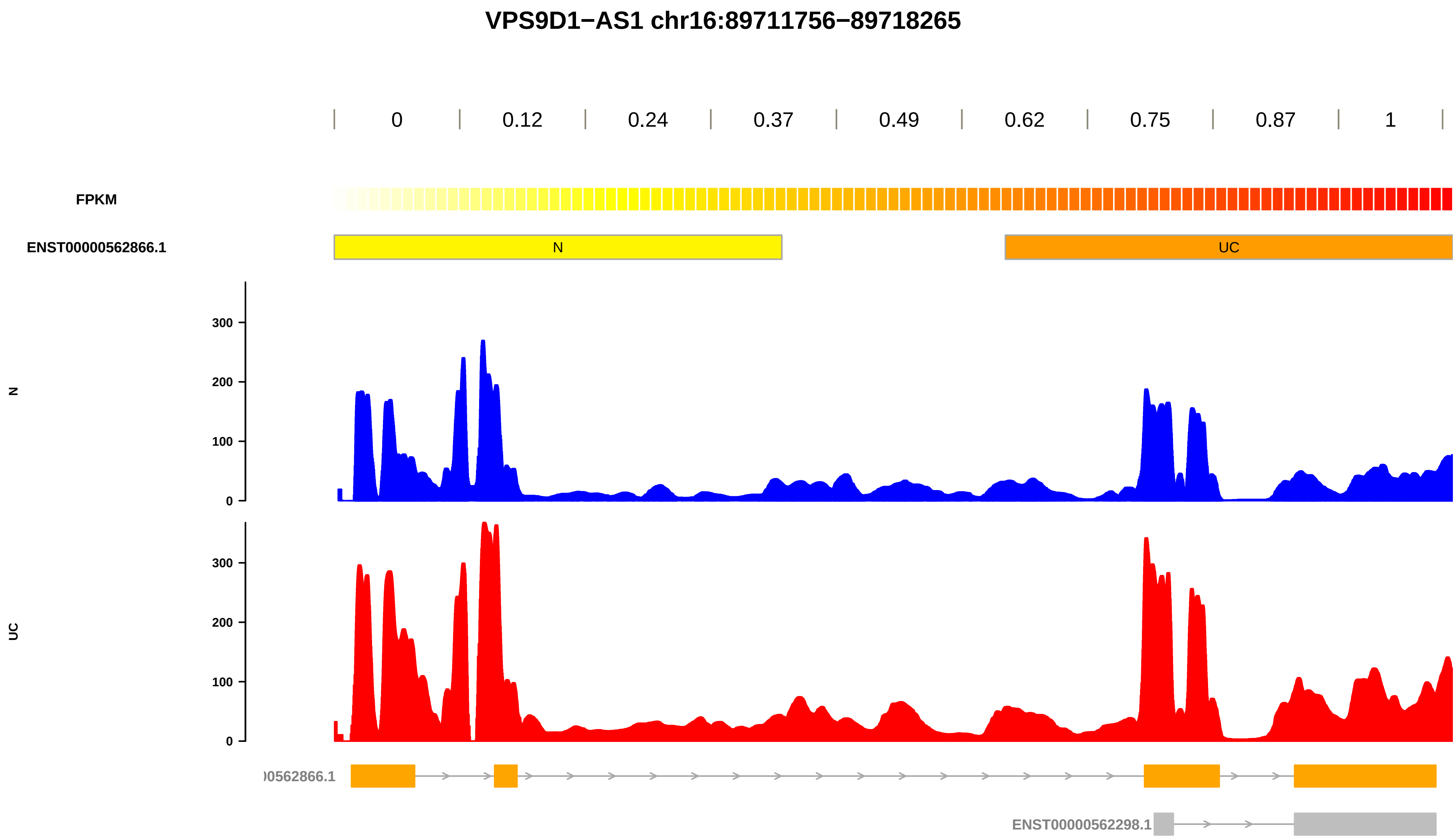

VPS9D1−AS1 chr16:89711756−89718265
0
0.12
0.24
0.37
0.49
0.62
0.75
0.87
1
FPKM
ENST00000562866.1
N
UC
N
UC
300
200
100
0
)0562866.1
ENST00000562298.1

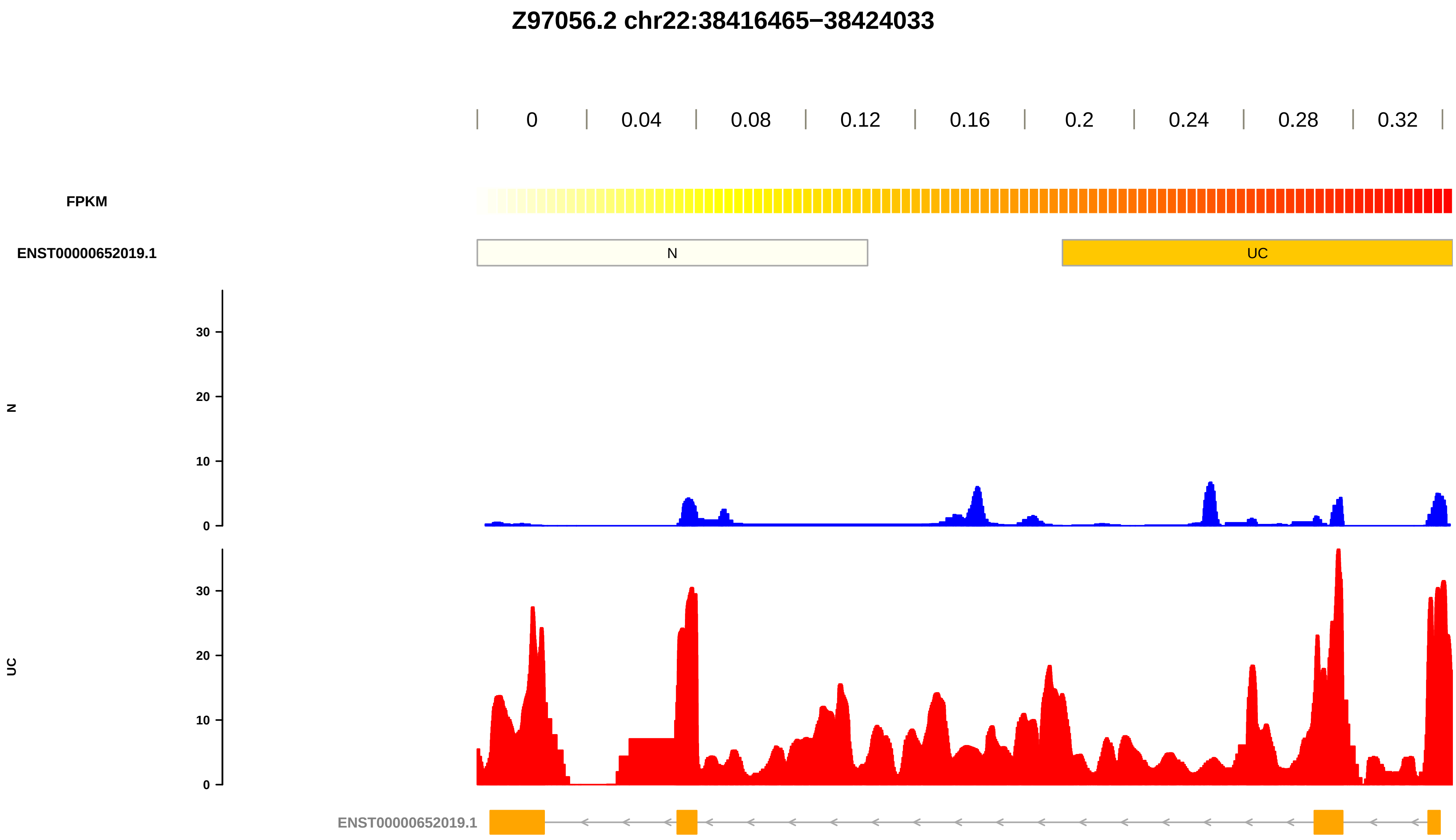
Z97056.2 chr22:38416465−38424033
0
0.04
0.08
0.12
0.16
0.2
0.24
0.28
0.32
FPKM
ENST00000652019.1
N
UC
N
30
20
10
0
UC
30
20
10
0
ENST00000652019.1
