# Supplementary material for: Novel long non-coding RNAs of relevance for ulcerative colitis pathogenesis
Source: Noncoding RNA Res. 2022 Feb 6;7(1):40–7. doi: 10.1016/j.ncrna.2022.02.001 (PMC8844606; doi:10.1016/j.ncrna.2022.02.001)
Supplement: Multimedia component 2 [file mmc2.pdf]

Supplementary figure 2. Heat map of expression profiles of lncRNAs in UC patients. Upregulated lncRNA transcript expression in UC is indicated in red. Down regulated lncRNA expression in UC is indicated in blue. No change in lncRNA transcript expression between UC and N is indicated in white. **(A)** 56 lncRNAs were significantly up-regulated, and (**B)** 44 were significantly down-regulated. LncRNAs transcripts that are uncharacterized are highlighted in grey.

Supplementary file 4

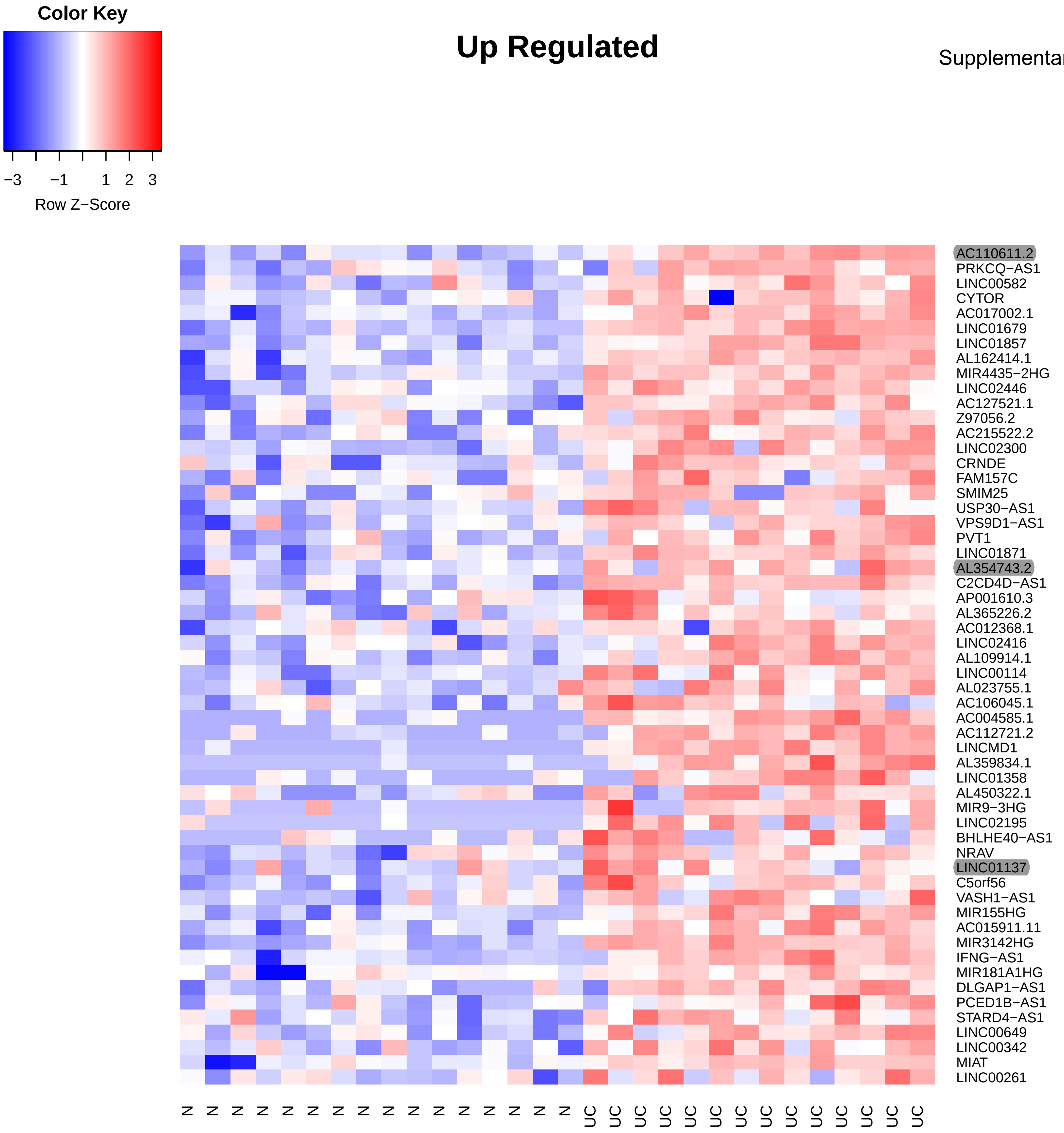

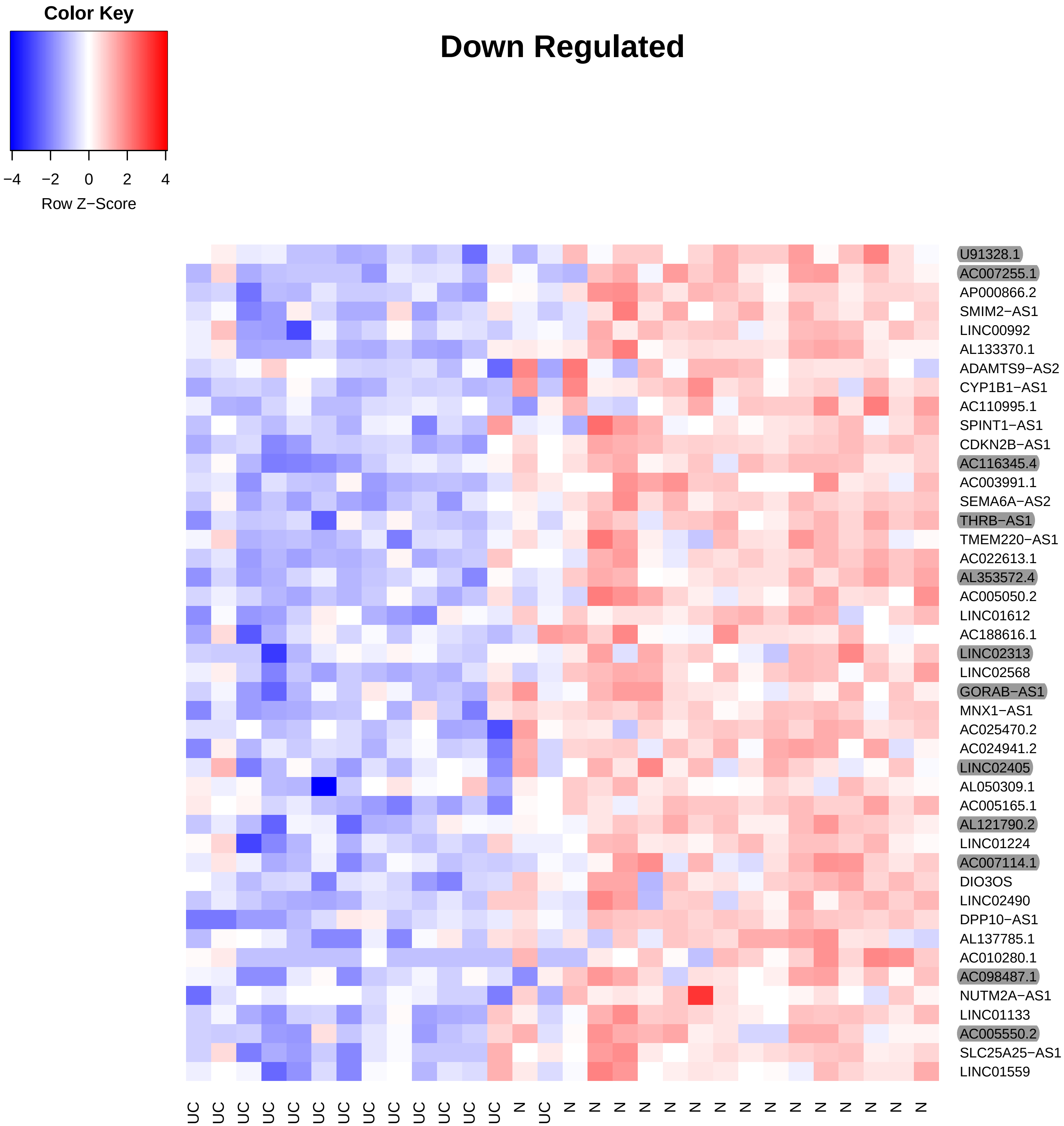

Color Key
−4 −2 0 2 4
Row Z−Score
Down Regulated
U91328.1
AC007255.1
AP000866.2
SMIM2−AS1
LINC00992
AL133370.1
ADAMTS9−AS2
CYP1B1−AS1
AC110995.1
SPINT1−AS1
CDKN2B−AS1
AC116345.4
AC003991.1
SEMA6A−AS2
THRB−AS1
TMEM220−AS1
AC022613.1
AL353572.4
AC005050.2
LINC01612
AC188616.1
LINC02313
LINC02568
GORAB−AS1
MNX1−AS1
AC025470.2
AC024941.2
LINC02405
AL050309.1
AC005165.1
AL121790.2
LINC01224
AC007114.1
DIO3OS
LINC02490
DPP10−AS1
AL137785.1
AC010280.1
AC098487.1
NUTM2A−AS1
LINC01133
AC005550.2
SLC25A25−AS1
LINC01559
UC UC UC UC UC UC UC UC UC UC UC UC UC N UC N N N N N N N N N N N N N N N
